# Measurement of schizophrenia symptoms through speech analysis from PANSS interview recordings

**DOI:** 10.3389/fpsyt.2025.1571647

**Published:** 2025-06-24

**Authors:** Michelle Worthington, Georgios Efstathiadis, Vijay Yadav, Isaac Galatzer-Levy, Alan Kott, Emanuel Pintilii, Tejendra Patel, Colin Sauder, Inder Kaul, Stephen Brannan, Anzar Abbas

**Affiliations:** ^1^ Brooklyn Health, Brooklyn, NY, United States; ^2^ Department of Psychiatry, Yale University School of Medicine, New Haven, CT, United States; ^3^ School of Psychology, University of New South Wales, Sydney, Australia; ^4^ Google LLC, Mountain View, CA, United States; ^5^ Department of Psychiatry, Grossman School of Medicine, New York, NY, United States; ^6^ Signant Health, Iasi, Romania; ^7^ Bristol Myers Squibb, New York, NY, United States

**Keywords:** digital phenotyping, digital health measures, natural language processing, schizophrenia spectrum disorders, speech characteristics, psychosis

## Abstract

**Introduction:**

Speech is considered a clinically meaningful indicator of schizophrenia symptom severity and the quantification of speech measures has the potential to improve the measurement of symptoms. Speech collection for digital phenotyping is often dependent on platforms built using closed-source code and associated with patient and clinician burden. Here, we evaluate recordings of clinical interviews conducted as part of standard clinical trial procedures as reliable sources of patient speech for symptom assessment using digital phenotyping. We hypothesize that speech will be associated with schizophrenia symptom severity as measured by PANSS scores using PANSS interview recordings as a data source, in line with existing research showing these associations using dedicated speech collection platforms and proprietary processing pipelines.

**Methods:**

Positive and Negative Syndrome Scale (PANSS) interview recordings, collected during a Phase 2 schizophrenia clinical trial, are used to calculate speech characteristics using open source code. A total of 825 PANSS recordings from 212 participants were used in this study. Mixed effects models accounting for demographic variables and time were conducted to assess the relationship between speech characteristics and PANSS scores.

**Results:**

Our findings show strong relationships between the calculated speech characteristics and schizophrenia symptom severity. Positive symptoms were associated with greater amount of speech, faster speech, and shorter, less varied pauses. By contrast, negative symptoms were associated with decreased amount of speech, slower speech, and longer, more varied pauses.

**Discussion:**

A large sample of PANSS recordings was successfully processed using open source methods for phenotyping and strong relationships between speech characteristics and symptoms from these recordings were observed. These observations, consistent with existing understandings of speech-based manifestations of schizophrenia, highlight the potential use of patient speech collected passively during clinical interactions for digital phenotyping and symptom assessment. Implications for clinical practice, drug development, and progress towards precision psychiatry are discussed.

## 1 Introduction

Accurate measurement of psychiatric symptom severity is important to both patient care and development of novel treatments. In the progress towards digital phenotyping of schizophrenia (1), speech has been demonstrated as a valuable objective measure of symptom severity in comparison to subjective clinician review (2).

Individuals with schizophrenia experience disordered thought and disorganized speech, perceptual abnormalities, unusual thought content, and negative symptoms, all of which may be associated with different aspects of speech (3–5). Positive symptoms such as disorganization and tangentiality are primarily associated with differences in linguistic features such as semantic coherence and speech complexity (6) as well as differences in acoustic features such as pitch variability (7). Negative symptoms such as alogia and blunted affect generally manifest in an underproduction of speech and are associated with features including greater pause length (8), differences in turn-taking behavior in clinical interviews (9), decreased amount and rate of speech (10), and decreased word connectedness (11). Despite promising findings in research settings, collection of patient speech and subsequent quantification of such speech characteristics outside of research settings can be challenging.

Several smartphone-, tablet-, and web-based platforms have been developed for targeted collection of elicited speech behavior and the subsequent analysis of speech characteristics (12–14). These platforms have the added benefit of allowing for remote, high-frequency assessment of speech, enabling ecologically valid assessment of symptom severity in comparison to in-clinic evaluations (15). However, such platforms are often founded on proprietary and closed-source code, limited in their context of use (e.g., early-stage clinical trials), and associated with significant burden to both patients and clinicians, leading to poor data quality and lack of adherence (16, 17).

Opportunities to passively record patient speech are growing in both clinical practice and drug development. In psychiatry clinical trials, interviews conducted during study visits are often recorded as part of standard practice (18). In care delivery, proliferation of telehealth (19) and adoption of digital scribing (20) has normalized collection of audio. If patient speech recorded in these contexts can reliably be used for digital phenotyping of schizophrenia (and psychiatric symptoms more broadly), it allows for a more practical and scalable implementation of digital phenotyping as part of both clinical practice and drug development.

Here, we evaluate recordings of Positive and Negative Syndrome Scale (PANSS) (21) interviews as viable sources of patient speech for digital phenotyping of schizophrenia. PANSS recordings collected as part of a Phase 2 clinical trial in schizophrenia (22) are used to calculate speech characteristics from study participant voice. The primary aim of this retrospective analysis is to determine whether PANSS recordings can serve as a reliable data source for speech phenotyping by examining the associations between speech features and symptoms in this dataset. To assess the scalability of using such interviews as a reliable data source, we leverage a fully automated processing pipeline, including speech transcription and diarization. The need for automated speech analysis methods has been outlined previously (8, 23–25) and the value in psychiatric research has been demonstrated (6, 26, 27), despite the expected error and subsequent noise in analysis introduced through automated methods. Here, we aim to demonstrate the possibility of these pathways identifying meaningful patterns related to symptom severity in line with previous research.

We hypothesize that speech will be associated with schizophrenia symptom severity as measured by PANSS scores, which would demonstrate feasibility of using speech characteristics to measure symptom severity. In line with the relationships found in the existing literature as described above, we expect to see negative symptoms associated with a reduction in amount of speech, greater pause duration, and slower speech rate, which may reflect symptom dimensions such as blunted affect and alogia (8–11, 28). The relationship between positive symptoms and speech features including amount of speech, pause characteristics, speech rate, and emotional sentiment is less well understood (11) but pause characteristics and amount of speech may play a specific role in differentiating positive and negative symptoms (29). Given this, observing differential associations between speech features and both positive and negative symptoms will demonstrate sensitivity and discriminant validity for characterizing distinct symptom presentations.

Our analysis shows a strong relationship between the calculated speech characteristics and symptom severity (10, 30, 31) as measured by the PANSS, demonstrating viability of PANSS recordings as a speech phenotyping data source for certain speech characteristics. The quality of these recordings allowed for the analysis of speech characteristics but not vocal acoustic features. Given the retrospective nature of this analysis that uses speech samples collected without dedicated protocols for speech collection or consistency in recording setup and equipment, factors that may influence the analysis of the timing, rate, amount, and sentiment of speech are expected to be less impacted than vocal acoustics. These findings have implications for how digital phenotyping from recordings collected as part of standard operations can be used in drug development. They also suggest the implementation of speech-based digital phenotyping as part of clinical practice, potentially enabling precision psychiatry (32) and measurement-based care (33).

Importantly, our study relies only on open source code. We use OpenWillis, an open source Python library for digital health measurement (www.github.com/bklynhlth/openwillis) (34), for audio processing and calculation of speech characteristics. This enables independent replication of our work, including using data collected from varying contexts. This allows for trust, standardization, and interoperability of methods in digital phenotyping.

## 2 Materials and methods

A Jupyter notebook containing the code for and findings from this study can be found here: www.github.com/anzarabbas/manuscripts.

### 2.1 Data and participants

Speech data was collected as part of a 5-week Phase 2 schizophrenia clinical trial that screened 250 participants and enrolled 182 participants, sponsored by Karuna Therapeutics, with ClinicalTrials.gov Identifier: NCT03697252 (22). As part of the study, the PANSS was administered at each of the 5 study visits to assess symptom severity (screening, baseline, days 14, 28, and 35). PANSS interviews were audio recorded as part of standard practice and informed consent was obtained to do so. Audio was recorded using a tablet through an electronic clinical outcome assessment (eCOA) platform developed by Signant Health (www.signanthealth.com).

### 2.2 Audio processing

To ensure participant privacy, audio recordings were transcribed and processed locally on Signant Health’s servers using a pipeline developed by Brooklyn Health (www.brooklyn.health) for calculation of participant speech characteristics. The HIPAA-compliant processing pipeline uses OpenWillis, an open source Python library for digital health measurement (www.github.com/bklynhlth/openwillis) (34). Once the audio files were processed locally, only the output variables reported here were transferred to Brooklyn Health for further analysis. The steps used by the pipeline are described below.

#### 2.2.1 Speech transcription and diarization

First, the speech transcription cloud v1.1 function in OpenWillis was used to convert speech into text. This step in the processing did not use open source methods as the function relies on Amazon Web Services’ (AWS) Amazon Transcribe (aws.amazon.com/pm/transcribe/) to generate transcripts. However, open source alternatives are available in OpenWillis and otherwise if researchers wish to replicate these methods without using AWS. The transcripts are diarized, which means they contain unique identifiers for each speaker in the audio file. Given the audio was of a PANSS interview between a clinician and a study participant, two unique speakers were expected. The transcripts also contain word-level timing information, necessary for calculation of the speech characteristics described below.

#### 2.2.2 Speaker identification

Next, the speakers were identified as either the clinician or the participant using the same OpenWillis function. PANSS interviews were administered in accordance with the Structured Clinical Interview – Positive and Negative Syndrome Scale (SCI-PANSS) (36) and the expected clinician prompts were known. Using this knowledge, the function compared each individual’s speech with the expected prompts and labeled the clinician based on similarity to the expected SCI-PANSS prompts. This comparison is done using a pre-trained sentence transformer model, which maps sentences into an embedding space based on their underlying meaning and compares them using cosine similarity (37). The closer the embeddings, the more similar the speech is in meaning.

#### 2.2.3 Calculation of speech characteristics

Finally, the speech characteristics v3.0 function in OpenWillis was used to derive speech characteristics from the participant’s transcript. Four categories of speech characteristics were calculated: amount of speech, rate of speech, pause characteristics, and emotional sentiment. Each of the speech characteristics are listed and described in **Table 1**. A more detailed description of the methods used to calculate them can be found in the documentation for the speech characteristics v3.0 function in OpenWillis. As mentioned previously, these characteristics were selected because of the existing evidence in the literature on their relationship with schizophrenia symptom severity (10, 30, 31). All features not directly related to speech length are adjusted for interview length given the potential confound of the interview context.

**Table 1:** Characteristics measured from participant speech during PANSS interviews.

Category	Characteristic	Description
Amount of speech	Speech length, minutes	Total time spent speaking by participant, in minutes
	Speech length, words	Total words spoken by the participant during the interview
	Speaker percentage	Percentage of file containing speech by identified speaker
	Mean turn length, minutes	Average duration of participant’s responses, in minutes
	Mean turn length, words	Average word count of participant’s responses
Rate of speech	Words per minute	Total words in a turn divided by turn length in minutes
	Syllables per minute	Total number of syllables divided by turn length in minutes
Pause characteristics	Word pause length, mean	Mean length of pauses between words in seconds
	Word pause variability	Variability in the length of pauses between words
	Mean pre-turn pause	Average time elapsed between questions and answers in seconds
Emotional sentiment*	Positive sentiment	Amount of positive sentiment detected in speech
	Negative sentiment	Amount of negative sentiment detected in speech

*Calculated using a pre-trained model that predicts emotional valence from speech (56)

A detailed description of the methods used can be found in the documentation for the speech characteristics v2.0 function in OpenWillis.

In our analysis pipeline, the automated process of diarization involves the exhaustive assignment of each segment of text to each speaker, which may preclude the measurement of other meaningful features related to conversation dynamics, including overlapping speech (35). In this study, conversation dynamics are reflected in pause characteristics, which are measured at both the word level and turn level. To demonstrate how the pipeline assigns speakers and calculates pause characteristics, we have provided a thorough methodological explanation and demonstration in the **Supplementary Data** (**Supplementary Methods 1**). Here, we also provide information for accessing sample data from a mock interview, including associated transcriptions, to facilitate review and replication of the pipeline and results.

### 2.3 Statistical analysis

#### 2.3.1 Distributions

The distribution of values for each of the speech characteristics across all recordings was reported using density plots. Mean, standard deviation, and kurtosis of each variable were measured.

#### 2.3.2 Effect of age, sex, and race

Each of the speech characteristics were evaluated for their relationship with age, sex, and race. These analyses were limited to the screening visit to avoid the use of repeated measures. For age, Pearson correlations were conducted between each speech characteristic and participant age. For sex and race, *t*-tests were conducted to compare the values of each speech characteristic across sex and race.

#### 2.3.3 Relationship with clinical scores

The relationship between speech characteristics and clinical scores was evaluated accounting for time point and demographic variables including age, sex, and race. Linear mixed effects models were conducted with the clinical score as the dependent variable and the speech characteristic along with age, sex, and race of the participant as fixed effects. Subject ID and visit were included as random effects. Visit was treated as a random effect due to the changing nature and length of conversations throughout the study, which also saw changes in behavior and symptoms due to the drug’s effect; the reported features, beyond total speech length, account for speech length, mitigating the influence of interview length on other linguistic variables. Model coefficients and partial eta-squared values were used to evaluate the effect size and variance explained by each speech characteristic in predicting clinical scores after accounting for demographics and visit. A total of 12 speech features were evaluated for their association with both positive and negative symptoms. To account for multiple comparisons, the false discovery rate (FDR) correction was implemented to ensure appropriate thresholds for significant relationships.

The clinical scores used were the PANSS Positive Scale (PANSS-P) and the PANSS Negative Scale (PANSS-N). The study did not focus on the overall PANSS score as it was hypothesized that many of the speech characteristics would have opposite relationships with positive and negative symptoms. Given the PANSS total score is partly a sum of PANSS-P and PANSS-N, results from such a comparison would be difficult to interpret.

To assess the relationship between speech characteristics and clinical scores independent of time, we also conducted ordinary least squares regressions predicting PANSS-P and PANSS-N scores with the averaged values of speech measures over the screening and baseline visits as predictor variables and age, biological sex, and race as covariates. As this analysis did not contain repeated measures, relationships between speech measures and clinical scores were not affected by the observed change in symptom severity over time. Averaging across screening and baseline was not considered problematic as it is assumed participants are stable in their symptom profile across the two visits prior to intervention.

## 3 Results

### 3.1 Study participants

Details on the study population can be found in Brannan et al. (2021) (22). PANSS recordings from 218 screened participants and 176 enrolled participants were used in this study. Of those screened, 165 were male and 53 were female. The mean age was 42.8 years, ranging from 19 to 60, with a median age of 44. 171 participants identified as Black, 47 identified as White, and 9 identified as Asian, Native American, or other. The majority of recordings were successfully processed for speech analysis, as is explained below. A breakdown of participant demographics for the data that was processed is provided in **Table 2**.

**Table 2:** Breakdown of the number of participants with whose PANSS recordings were successfully processed at each study visit along with demographic information.

Visit	Recordings (*n*)	Processed (*n*)	Length (µ)	Age (µ)	Female (%)	Black (%)
Screening	224	216	39.2	42.3	24.7	75.3
Baseline	174	173	39.2	42.5	22.8	75.5
Day 14	157	156	34.7	42.8	22.9	74.1
Day 28	152	143	31.8	42.9	22.4	76.3
Day 35	143	137	30.7	42.9	23.5	76.6
All visits	850	825	35.7	42.6	23.4	75.62

The unit for length is minutes.

### 3.2 PANSS interview processing

Of the 850 PANSS interviews conducted across all study visits, 825 were available for analysis and were successfully processed by the pipeline. A total of 25 interviews did not have corresponding clinical data available for analysis. On average, the length of each processed recording was 33.5 minutes. The total number of interviews processed and analyzed for each visit are described in **Table 2**.

### 3.3 Speech characteristics distributions

The distributions of each of the speech characteristics across all processed recordings in the dataset are presented in **Supplementary Figure 1**. All measures exhibited kurtosis within the range -1 to 1. In alignment with the guidance in DeCarlo (1997) (38), parametric statistical tests were employed across all analyses.

### 3.4 Effect of age, sex, and race

A subset of speech characteristics showed a significant relationship with age at screening, including between-word pause variability (R^2^ = 0.034; *p* = 0.01; *n* = 189) and positive sentiment (R^2^ = 0.03; *p* = 0.01; *n* = 209), both of which decreased with age. Female participants exhibited greater speech length in words (*p* < 0.01), longer turn length in words (*p* = 0.01), greater speaker percentage (*p* < 0.01), faster rate of speech in words (*p* < 0.01) and syllables (*p* < 0.01), and reduced pause length between words (*p* < 0.01) compared to male participants. White participants exhibited greater speaker percentage (*p* = 0.03), greater syllables per minute (*p* = 0.03), reduced word pause length mean (*p* = 0.02), and reduced mean pre-turn pause (*p* = 0.01) as compared to black participants. Graphs are presented in **Supplementary Figure 2**.

### 3.5 Relationship with positive symptoms

**Figure 1:**
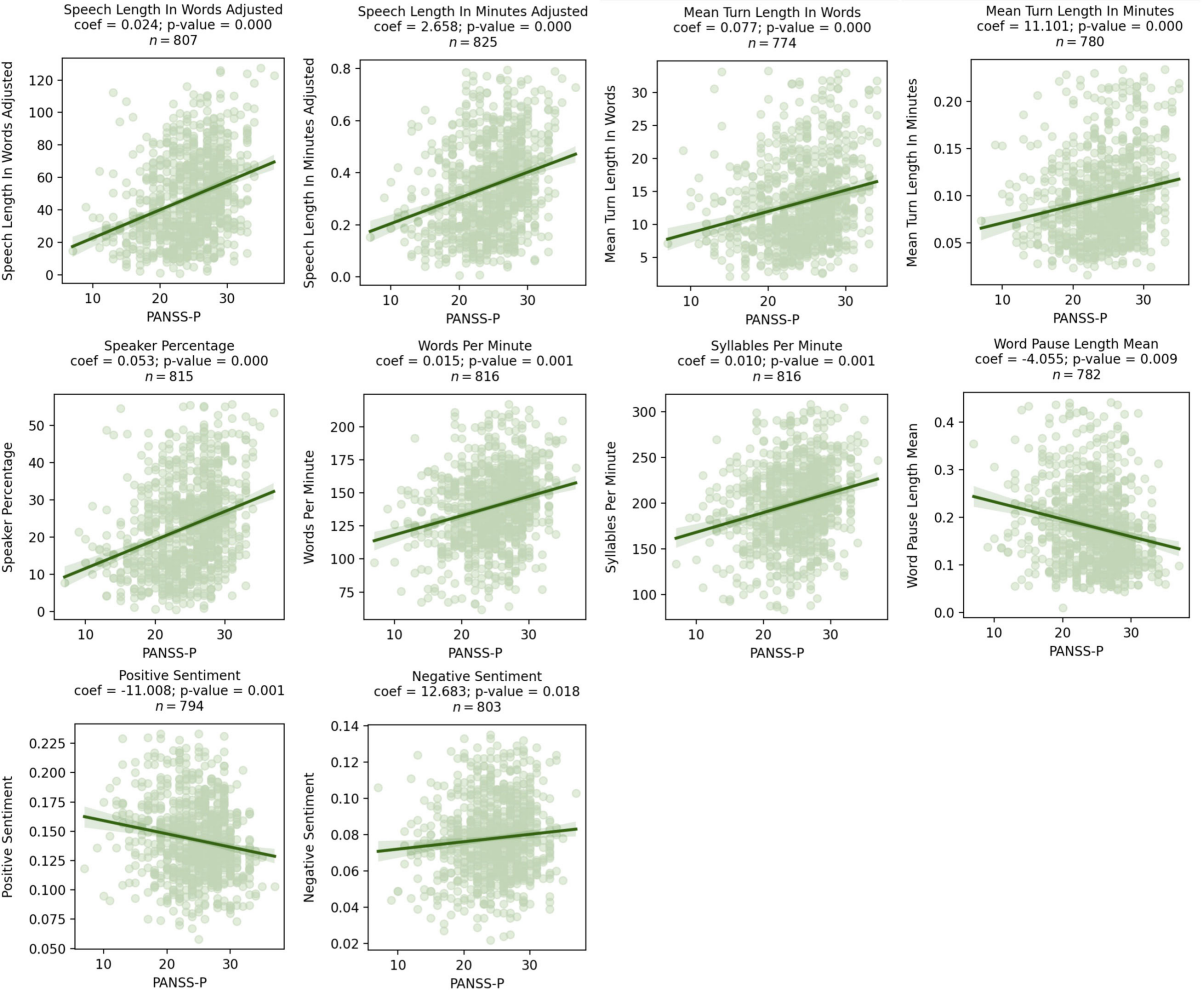
Scatter plots showing significant relationships between PANSS-P and speech characteristics as assessed by mixed effects models. Coefficients for the speech characteristics from the mixed effects model are shown above the graph, where visit, age, sex, and race were included as covariates.

Across all visits, higher PANSS-P scores were associated with a greater amount of speech (mean turn length in words β = 0.08, *p* = 0.02*)*, shorter pauses (mean pause length in seconds β = -4.06, *p* = 0.01), and decreased positive emotional sentiment (β = -11.01, *p* = 0.01). Results from mixed effects models that included data from the entire study and had coefficients with a *p*-value < 0.05 are presented in **Figure 1**. In these figures, coefficients represent the increase/decrease in speech features per 1 point increase/decrease in PANSS-P. Detailed statistical results are presented in **Table 3**. All associations survived the FDR correction.

**Table 3:** Statistics of mixed effects models assessing the relationship between speech characteristics and clinical scores for the entire study.

Category	Speech characteristic	PANSS-P				PANSS-N			
		Direction of association	β	η_p_^2^	*p*-value	Direction of association	β	η_p_^2^	*p*-value
Amount of speech	Speaker percentage	+	0.05	0.036	**<0.001**	–	-0.05	0.029	**<0.001**
	Speech length, minutes	+	2.7	0.024	**<0.001**	–	-1.91	0.013	**0.004**
	Speech length, words	+	0.02	0.036	**<0.001**	–	-0.02	0.025	**<0.001**
	Mean turn length, minutes	+	11.1	0.028	**<0.001**	–	-10.6	0.025	**<0.001**
	Mean turn length, words	+	0.08	0.028	**<0.001**	–	-0.09	0.034	**<0.001**
Rate of speech	Words per minute	+	0.02	0.016	**0.001**	–	-0.02	0.017	**0.001**
	Syllables per minute	+	0.01	0.015	**0.001**	–	-0.01	0.015	**0.001**
Pause characteristics	Word pause length, mean	–	-4.1	0.010	**0.01**	+	7.3	0.032	**<0.001**
	Word pause variability		-0.71	0.006	0.05	+	1.00	0.011	**0.008**
	Mean pre-turn pause		-0.38	0.005	0.06	+	0.48	0.008	**0.02**
Emotional sentiment	Positive sentiment	–	-11.01	0.018	**0.001**		-3.03	0.001	0.37
	Negative sentiment	–	12.9	0.009	**0.01**	+	11.6	0.008	**0.03**

Statistics from mixed effects models are shown for speech characteristics after accounting for visit, age, sex, and race including coefficients (β), partial eta-squared (η_p_^2^), and *p*-values. Results are shown separately for predicting positive symptoms (PANSS-P) and negative symptoms (PANSS-N). Significant relationships are bolded and direction of associations are shown for significant associations.

Results from the regression models predicting positive symptoms with speech characteristics that limited the analysis to averaged values from the screening and baseline visits showed similar patterns. Detailed results of these regression models are presented in **Supplementary Table 1**.

### 3.6 Relationship with negative symptoms

**Figure 2:**
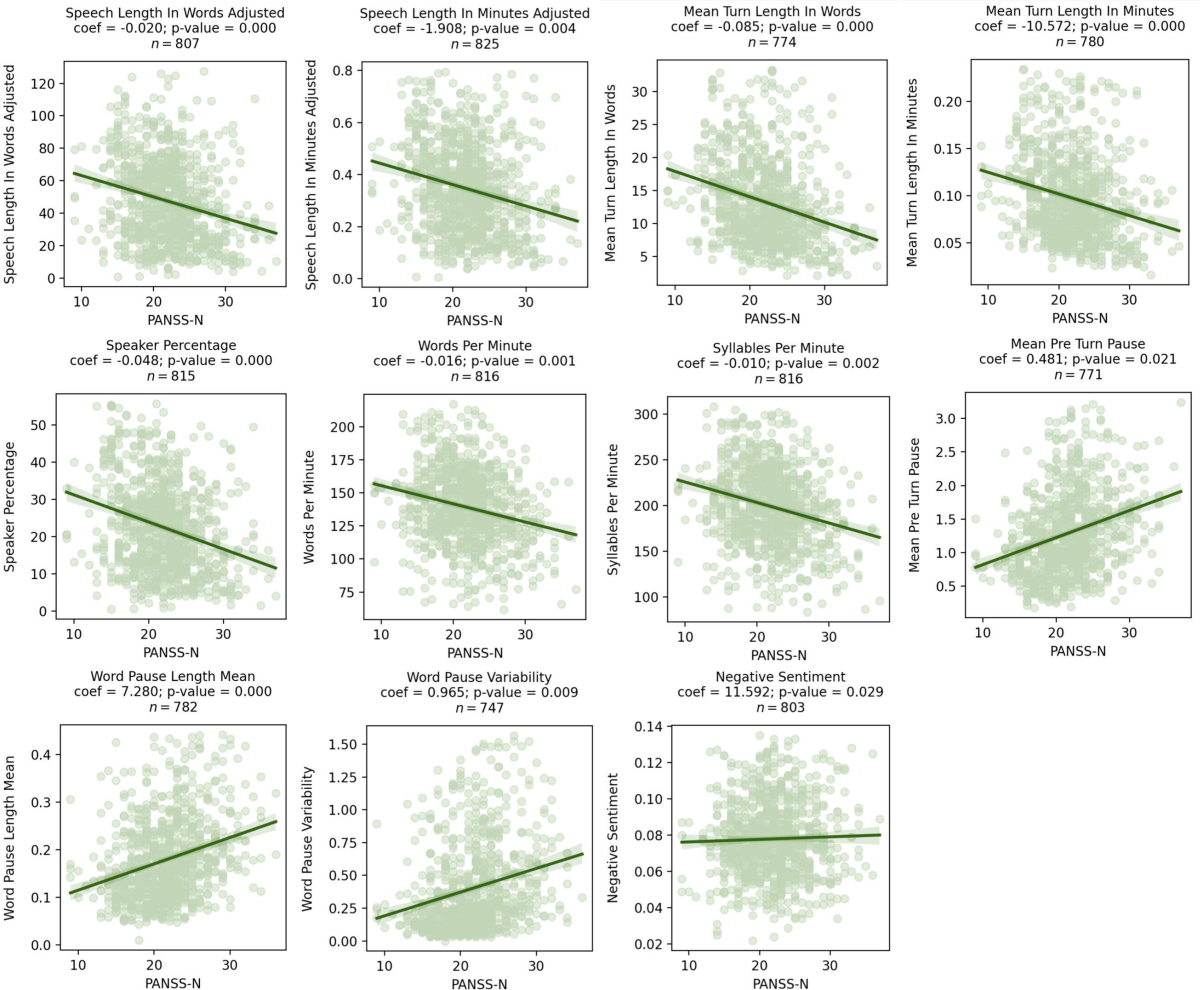
Scatter plots showing significant relationships between PANSS-N and speech characteristics as assessed by mixed effects models. Coefficients for the speech characteristics from the mixed effects model are shown above the graph, where visit, age, sex, and race were included as covariates.

Higher PANSS-N scores were associated with a decreased amount of speech (mean turn length in words β = -0.09, *p* = 0.01) and longer and more variable pauses (mean pause length in seconds β = 7.28, *p* = 0.02, pause variability β = 0.97, *p* = 0.01). Results from comparisons that included data from repeated measures and had a *p*-value < 0.05 are presented in **Figure 2**. In these figures, coefficients represent the increase/decrease in speech features per 1 point increase/decrease in PANSS-N. Detailed statistical results are presented in **Table 3**. All associations survived the FDR correction.

Results from the regression models predicting negative symptoms with speech characteristics that limited the analysis to averaged values from the screening and baseline visits showed similar patterns. Detailed results of these regression models are presented in **Supplementary Table 1**.

Notably, many speech characteristics with a significant relationship with PANSS-P showed significant inverse relationships with PANSS-N. See **Table 3** for summary of the directional relationships for all speech characteristics and clinical scores.

## 4 Discussion

### 4.1 Sample size

This study presents one of the largest attempts to evaluate speech in schizophrenia. With 825 PANSS interviews processed, we isolated 490 hours of participant speech for analysis. Of note, this study did not use dedicated recording equipment to collect speech, and yet nearly all of the total recordings available were evaluable. In doing so, we highlight the potential that recordings of clinical interactions hold for the study of speech-based manifestations of psychiatric illness. This applies to clinical trials, where such recordings are collected as part of standard practice. However, it extends to other contexts, such as phone calls or telehealth visits conducted during clinical practice, or in-person clinic visits where a microphone may already be present for digital scribing (20). If such datasets can be used to better understand behavioral manifestations of psychiatric illness, it addresses a major challenge in digital phenotyping, i.e., the collection of sufficient data for higher level model training.

### 4.2 Robustness of observations

We demonstrate a robust set of relationships between basic speech characteristics and schizophrenia symptom severity. Higher-order features involving more complex analysis of natural language, computational modeling, and the combination of acoustic and speech features, which may also robustly describe clinical features of schizophrenia (39–41), could be further explored in future studies. All relationships observed were in the hypothesized direction based on current understandings of the behavioral manifestations of positive and negative symptoms as well as existing literature on speech in schizophrenia (3, 7, 10, 29). Consistent with past research, we found associations between positive symptoms and increased amount of speech, shorter pauses and increased negative emotional sentiment, and between negative symptoms and decreased amount of speech, longer and more variable pauses, and increased negative emotional sentiment. In addition, although our models included race, age, and biological sex as covariates, the sample was predominantly black and male. Future research with similar interview data should address questions of specificity, reliability, and validity in larger datasets that contain robust clinical data across groups to account for any potential methodological bias.

### 4.3 Open source methods

The study calculated speech characteristics using open source code (www.github.com/bklynhlth/openwillis). Independent researchers may calculate speech characteristics from their own datasets using the same code library. Standardization of methods used to calculate speech characteristics allows for direct comparison of observations, critical to achieving replicability across studies. In addition to using open source code, we also published all code used to conduct the analysis presented (www.github.com/anzarabbas/manuscripts). This allows independent researchers to compare downstream analysis methods used to produce the observed results.

### 4.4 Operational simplicity

In the context of clinical trials, analyses of participant speech often rely on dedicated speech collection platforms (13). Such platforms may allow for at-home or more frequent data collection (42). However, they increase burden on study participants, who may already be overwhelmed (43), and require additional effort from clinical sites, tasked with executing sophisticated study protocols (44). Collectively, dedicated platforms add risk and operational complexity to a clinical trial, which may deter sponsors from including them in the study design despite their advantages. Our study demonstrates how existing data can be leveraged for additional measures, challenging the need for a dedicated speech collection platform if remote, high-frequency assessments are not critical.

### 4.5 Potential in drug development

Though our analysis was retrospective, it brings to light the potential of including such measures prospectively in clinical trial designs for improvement in the drug development process. Patient speech collected during pre-screening visits may help triage individuals brought in for an in-person screening visit to improve screen-failure rates (45). Screening itself may benefit from the use of speech to stratify individuals into subclasses, enabling precision drug development (7). The capacity for speech to predict scores on standard assessments such as the PANSS could be relied upon to identify potential problems, such as biased ratings (46). Speech may also be valuable in assessing treatment efficacy through analysis of specific behaviors difficult to quantify objectively using traditional scales (47), e.g., psychomotor slowing, semantic complexity, emotional sentiment.

### 4.6 Study limitations

Our study analyzed audio recordings collected between 2018 and 2019 with no special considerations towards their eventual processing for speech characteristics. Despite this, nearly all recordings were able to be processed for analysis of speech characteristics in our pipeline. Available recordings were not included in our analysis (n = 25) if corresponding PANSS scores were unavailable. A limitation remains regarding audio quality. Perhaps the most consequential impact of inconsistent audio quality was the inability to reliably measure acoustic properties of participant voice, known to be relevant in assessment of psychiatric functioning (48), particularly in schizophrenia (10). Recordings analyzed in this study varied greatly in the distance between the microphone and the study participant and relied on a relatively low-quality microphone. Hence, analysis of acoustic properties of voice could not be conducted. Future studies could introduce standardization in how clinical interviews are recorded and use higher quality microphones, which would allow for inclusion of vocal acoustics into the overall analysis of speech.

Second, the speech characteristics analyzed in this study (**Table 1**) were intentionally straightforward. The purpose of this study was to demonstrate that speech during a PANSS interview was sufficient in replicating known relationships between speech characteristics and schizophrenia symptom severity. Having demonstrated as such, future studies will include more novel measures, including higher order linguistic variables that have been reported more recently in the literature (40, 49). We propose such work in the section below on future directions.

Relatedly, we acknowledge the potential for errors in using automated transcription and diarization methods. Analyzing a large sample of speech requires scalable methods and a move away from manualized transcription methods. Existing automated speech recognition (ASR)-based transcriptions are known to produce some errors; however, a systematic comparison between ASR tools showed that the methods used in this paper (i.e., Amazon Transcribe) perform similarly or better than other popular ASR tools in both transcription and diarization tasks (50). To further assess the performance of automated transcription and diarization methods, we manually and then automatically transcribed excerpts from mock clinical interviews. Results of this test, which are reported in **Supplementary Figure 5**, show minimal errors between these two methods.

An advantage of dedicated speech collection platforms is elicitations of standardized forms of speech, such as sustained vowel phonation (51), reading of predefined passages (52), etc. This allows for targeted calculation of context-dependent acoustic or linguistic variables. Though the approach presented in this study does not target such variables, prospective deployments could implement elicitations of standardized speech, as is typical to some clinical assessments, e.g., those that evaluate motor functioning (53).

Finally, the statistical analysis was purposefully limited to mixed effects models to demonstrate associations between speech characteristics and clinical scores. Additional analyses of interest, including longitudinal and prediction-based analyses, are intentionally not reported. This includes use of machine learning to train higher level models that predict PANSS scores (54), the ability of such models to identify biased ratings on the PANSS, evaluating the capacity of speech characteristics to stratify individuals (28) as potential responders to drug/placebo, and their ability to serve as endpoints. We believe such analyses will benefit from training and testing models on independent datasets, work that is planned and forthcoming. This study achieves its central purpose, which was to demonstrate that PANSS interview recordings can be used to reliably observe speech characteristics indicative of symptom manifestations in schizophrenia.

### 4.7 Future directions

Though this study utilized PANSS recordings, PANSS are often not the only recorded clinical interviews conducted during a schizophrenia clinical trial. Future studies can include analysis of speech from additional recordings, such as those of the Mini International Neuropsychiatric Interview (MINI) (55). This would allow for an evaluation of speech collected from independent conversational contexts for its ability to predict schizophrenia symptom severity as measured by the PANSS. Such work could expand into evaluation of speech in less structured conversations, such as phone calls conducted as part of pre-screening efforts.

Future work planned will explore a broader set of speech characteristics. This includes higher order linguistic variables more recently reported on in the literature, such as syntactic complexity, semantic coherence, and word connectedness (11, 39, 40, 56). This work will be conducted on data from multiple clinical trials conducted as part of the same drug development program by Karuna Therapeutics, allowing for a significantly larger sample size and model training/testing on independent datasets, enabling analyses mentioned in the previous section that were excluded from this study.

Finally, future work should also continue to evaluate and improve automated methods for analysis given error inherent in these methods, which we know to affect fine-grained analysis of dialogue such as evaluating overlapping speech or turn-taking dynamics, which have been shown to characterize speech in schizophrenia samples (9, 35). The methods described in this paper detect instances of overlapping speech as measured by overlapping timestamps of turns for each speaker. The methods do not quantify the amount of overlapping speech as ASR methods exhaustively assign speaker labels for detected speech. Notably, when an interruption is detected, the pause timing is not included in the calculation of turn-level or overall pause characteristics described here.

Recent work shows promising results with novel automated methods for correcting transcription and speaker diarization (50, 57, 58), which will continue improving the scalability and validity of using these methods for a wide range of tasks related to speech analysis.

### 4.8 Conclusion

We demonstrate that PANSS recordings, collected as standard practice in psychiatry clinical trials, contain sufficiently rich information for digital phenotyping of speech in schizophrenia. Speech characteristics were calculated using OpenWillis, an open source Python library, and did not require the use of a dedicated speech collection and analysis platform. Our findings highlight the value of existing clinical interactions towards the advancement of digital phenotyping in psychiatry. Future work can utilize such large datasets that already exist. In doing so, it may overcome dataset size limitations that often restrict studies in the field from reporting more conclusive observations.

## Data Availability

The datasets presented in this article are not readily available because the raw audio files analyzed in this article are considered protected health information. Requests to access the datasets should be directed to Anzar Abbas, anzar@brooklyn.health.
