# Supplementary material for: Measurement of schizophrenia symptoms through speech analysis from PANSS interview recordings
Source: Front Psychiatry. 2025 Jun 24;16:1571647. doi: 10.3389/fpsyt.2025.1571647 (PMC12235459; doi:10.3389/fpsyt.2025.1571647)
Supplement: Supplementary file 1 [file DataSheet1.docx]

# Supplementary Data

**Supplemental Methods 1.**

In order to facilitate readers’ evaluation of the pipeline used in this paper, we have provided audio files for mock PANSS interviews with accompanying JSON transcript files to assess performance of transcription, diarization, and feature extraction, including calculating timings of pause characteristics. These files can be accessed and downloaded from the following GitHub repository: <https://github.com/bklynhlth/sample_data/tree/main/mock_interview>.

Below, we also provide a detailed description of how pauses are labeled handled, given the importance of distinguishing between inter-turn pauses and intra-turn pauses.

**Calculation of pauses**

In the following excerpt, we examine an exchange where the participant responds to a question about how troublesome their anxiety symptoms are (not depicted here). The participant says “I’ll say maybe 7 out of 10” followed by the clinician acknowledging with “Mm, OK.”

{

  "utterances": [

    {

      "speaker": "participant",

      "words": [

        { "word": "I'll", "start_time": 66.9, "end_time": 67.26 },

        { "word": "say", "start_time": 67.26, "end_time": 67.849 },

        { "word": "maybe", "start_time": 67.94, "end_time": 68.459 },

        { "word": "7", "start_time": 68.459, "end_time": 68.86 },

        { "word": "out", "start_time": 68.86, "end_time": 68.98 },

        { "word": "of", "start_time": 68.98, "end_time": 69.099 },

        { "word": "10", "start_time": 69.099, "end_time": 69.569 },

        { "word": ".", "start_time": null, "end_time": null }

      ]

    {

  "utterances": [

    {

      "speaker": "clinician",

      "words": [

        { "word": "Mm", "start_time": 70.48, "end_time": 70.489 },

        { "word": ",", "start_time": null, "end_time": null },

        { "word": "OK", "start_time": 70.5, "end_time": 70.819 }

      ]

    }

  ]

}

Within these two utterances, there are start and end times for each word specified by the automated speech recognition (ASR) tool. In the analysis presented in the manuscript, the focus is only on the participant’s speech and not the clinician’s speech

| Start time | End time | Speaker | Word |
| --- | --- | --- | --- |
| 66.9s | 67.26s | Participant | I’ll |
| 67.26s | 67.849s | Participant | say |
| 67.849s | 67.94s | n/a | [between-word pause] |
| 67.94s | 68.459s | Participant | maybe |
| 68.459s | 68.86s | Participant | 7 |
| 68.86s | 68.98s | Participant | out |
| 68.98s | 69.099s | Participant | of |
| 69.099s | 69.569s | Participant | 10 |
| 69.569s | 70.48s | n/a | [pre-turn pause] |
| 70.48s | 70.489s | Clinician | Mm |
| 70.489 | 70.5s | n/a | [between-word pause] |
| 70.5s | 70.819s | Clinician | OK |

These two utterances, labeled by speaker (clinician vs. participant), demonstrate the way in which pauses are treated between words and between turns. The blue rows indicate inter-word pauses within a turn. These are averaged at the turn level and then across the audio file, resulting in the between-word pause rate described in the paper. The yellow row demonstrates a pre-turn pause, which is calculated between turns and averaged across the interview, for each speaker, to determine the average pre-turn pause rate for each speaker, which is reported just for participants in the manuscript. In the full JSON files, linked above, turns, utterances, and words are tabulated separately. Pre-turn pauses are calculated only on turns, which may consist of multiple utterances. Between-word pauses are only calculated on words within the same utterance. Full methods are described in detail in the OpenWillis documentation^1^.

**Speech overlap and interruptions**

As mentioned above, the pipeline distinguishes between word-level pauses, which are shown in the table above, and inter-turn pauses / pre-turn pauses, which are discussed more in this section and shown in the table below. In the above section, there is no overlap between turns. In the example below, we observe overlapping turns:

{

  "utterances": [

    {

      "speaker": "participant",

      "words": [

        { "word": "I", "start_time": 21.55, "end_time": 21.68 },

        { "word": "haven't", "start_time": 21.68, "end_time": 22.01 },

        { "word": "really", "start_time": 22.01, "end_time": 22.28 },

        { "word": "had", "start_time": 22.28, "end_time": 22.54 },

        { "word": "that", "start_time": 22.54, "end_time": 22.71 }

      ]

    },

    {

      "speaker": "clinician",

      "words": [

        { "word": "Right", "start_time": 22.65, "end_time": 23.05 }

      ]

    },

    {

      "speaker": "participant",

      "words": [

        { "word": "experience", "start_time": 22.91, "end_time": 23.21 },

        { "word": "this", "start_time": 23.21, "end_time": 23.31 },

        { "word": "week", "start_time": 23.31, "end_time": 23.89 },

        { "word": ".", "start_time": null, "end_time": null }

      ]

    },

    {

      "speaker": "clinician",

      "words": [

        { "word": "Right", "start_time": 24.75, "end_time": 25.15 },

        { "word": ".", "start_time": null, "end_time": null },

        { "word": "And", "start_time": 25.45, "end_time": 25.65 },

        { "word": "how", "start_time": 25.65, "end_time": 25.87 },

        { "word": "has", "start_time": 25.87, "end_time": 26.06 },

        { "word": "your", "start_time": 26.06, "end_time": 26.27 },

        { "word": "mood", "start_time": 26.27, "end_time": 26.57 },

        { "word": "been", "start_time": 26.57, "end_time": 27.26 },

        { "word": ",", "start_time": null, "end_time": null },

        { "word": "this", "start_time": 27.63, "end_time": 27.84 },

        { "word": "past", "start_time": 27.84, "end_time": 28.13 },

        { "word": "week?", "start_time": 28.13, "end_time": 28.49 },

        { "word": "?", "start_time": null, "end_time": null }

      ]

    }

  ]

}

Here are the timings of the turns:

| Start time | End time | Speaker | Transcription |
| --- | --- | --- | --- |
| 21.55s | 23.89s | Participant | I haven’t really had that experience this past week. |
| 22.65s | 23.05s | Clinician | Right. |
| 23.89s | 24.75s | n/a | [pause] |
| 24.75s | 28.49 | Clinician | Right. And how has your mood been, this past week? |

The clinician interjects “Right” before the participant is finished speaking, resulting in a short period of overlapping speech, as indicated by overlapping timestamps highlighted in red. The clinician pre-turn pause period is indicated in blue (although, because this pause precedes a clinician’s turn, it would not be counted toward the metrics used to evaluate participant pre-turn pause). The instances highlighted in red are flagged as *interruptions* and are not counted toward pre-turn pause calculations.

*The first type of diarization error*

Diarization errors can happen when there is a simple misattribution of a word between speakers within a turn. We know this happens often, as we have reported^2^. Future work will benefit from the implementation of a diarization correction model to improve the accuracy and confidence of attributing words to speakers. This model was not yet developed at the time of data analysis for this study, and thus was not implemented here.

When these errors do occur, an inter-word pause may be mistaken for an inter-turn pause. This type of error is a problem because we expect inter-word pauses to be shorter than inter-turn pauses and it would make the average inter-turn pause seem lower than it actually was.

Despite this, our hypothesis on inter-turn pauses based on previous literature was confirmed, which suggests that the signal with this feature was detectable despite the noise introduced from this type of error.

*The second type of diarization error*

Diarization errors can happen when there is a misattribution of words during turns. We know this happens often, as we have reported^2^, and will also be able to improve this error using the diarization correction model described here in future work.

If this does indeed happen, an inter-turn pause may be mistaken for an inter-word pause, and inter-word pauses for the rater may be confused as inter-word pauses for the participant.

This is problematic as it would make inter-word pauses longer than they actually are and sometimes not be reflective of participant behavior at all. It would also make inter-turn pauses shorter than they actually are.

Despite this, our hypothesis on inter-turn and inter-word pauses based on previous literature was confirmed, indicating that the signal of the association was greater than the noise introduced from this step.

*The third type of diarization error*

Diarization errors can also happen because there is speech overlap and the ASR is not sensitive enough to detect these instances, and it can lead to multiple types of errors. Our pipeline will flag instances of interruptions, but does not tabulate the amount of overlapping speech, as other studies using manual transcription methods have done. As opposed to spontaneous conversations, PANSS interviews are administered by trained raters in clinical trials who are instructed not to interrupt unless necessary for redirection. In addition, the ASR may exhaustively assign speaker labels for detected speech, as opposed to indicating overlap, which contributes to a low tabulation of interruptions, but, importantly, does not falsely assign inter-word or inter-turn pauses where none should be assigned. This could introduce some noise or difference as compared to existing literature on turn-taking dynamics using manualized methods, which we acknowledge. However, the signal we observed in pause measurements despite this noise aligns with previous research, suggesting that the error introduced with this source of noise may have been minimal compared to the strength of the signal.

1. <https://openwillis.brooklyn.health/15883a8fe04780739400c1d8ad94bb39?v=1c883a8fe04780e8ad8e000cf4950c5a>
2. Efstathiadis, G., Yadav, V., & Abbas, A. (2025). LLM-based speaker diarization correction: A generalizable approach. Speech Communication, 103224.

**Supplementary Figure 1.** Density plots for the values observed for each speech characteristic analyzed. Within each plot, the mean (µ), standard deviation (σ), and kurtosis for the distribution are displayed.

| 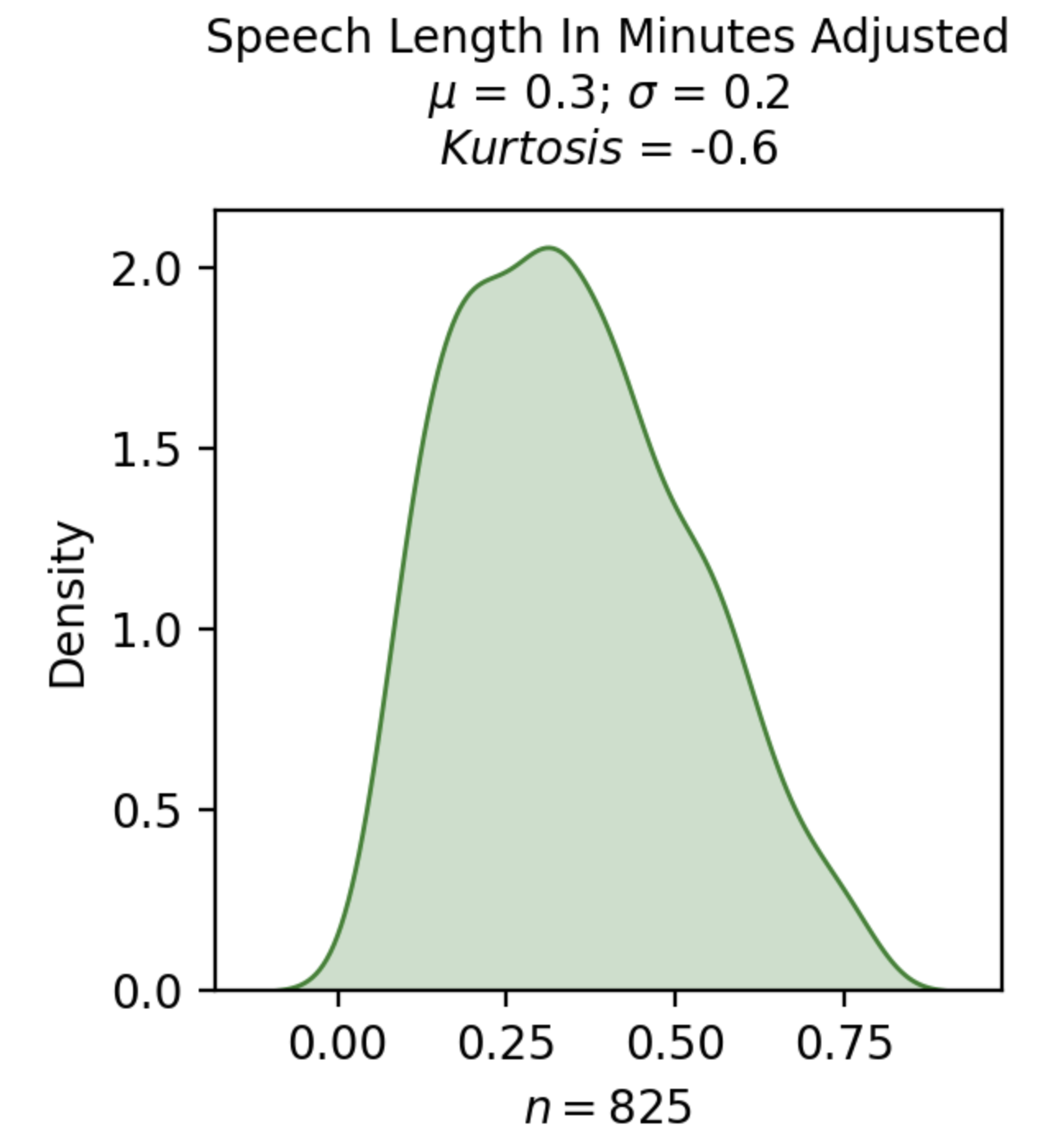 | 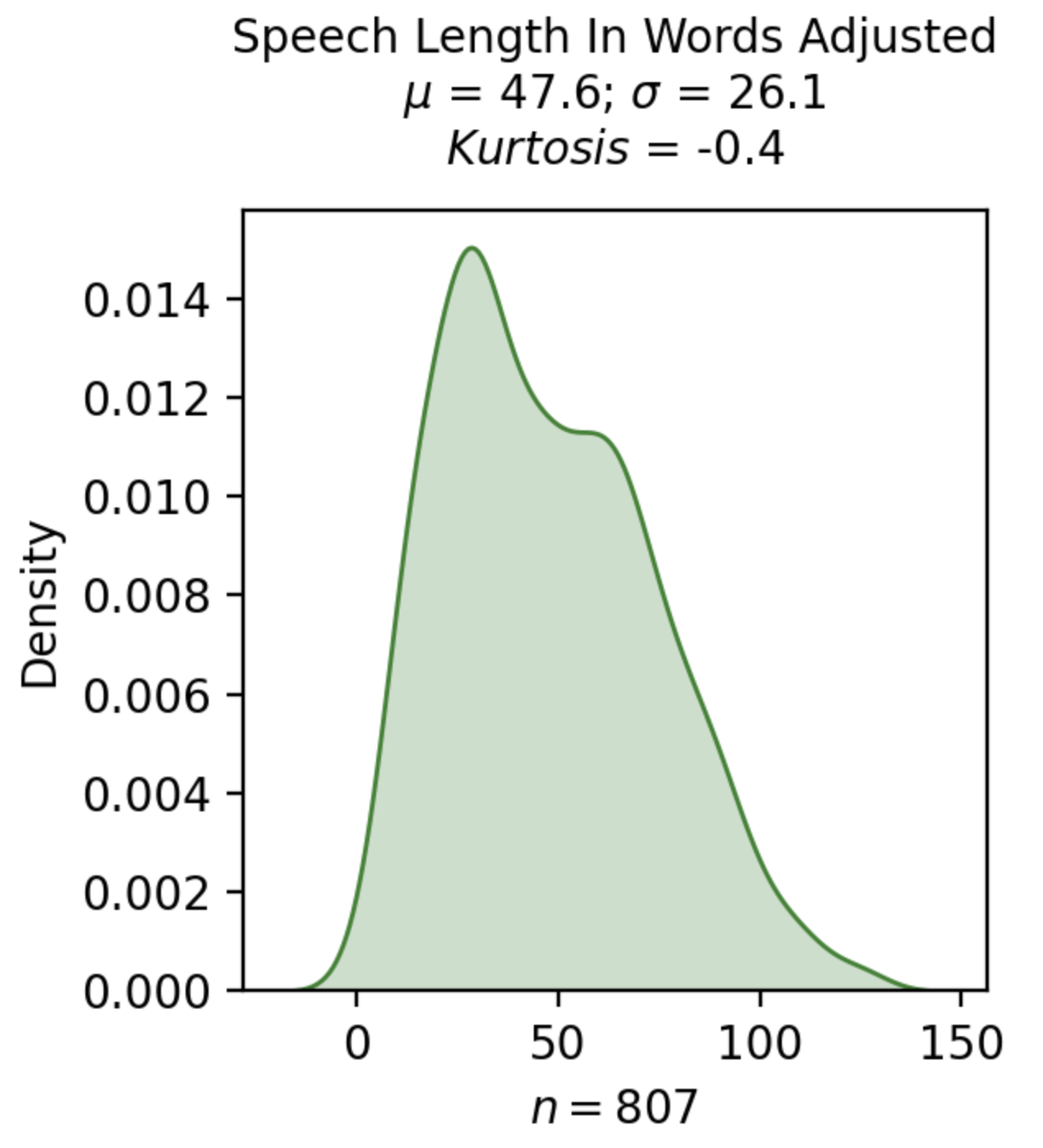 | 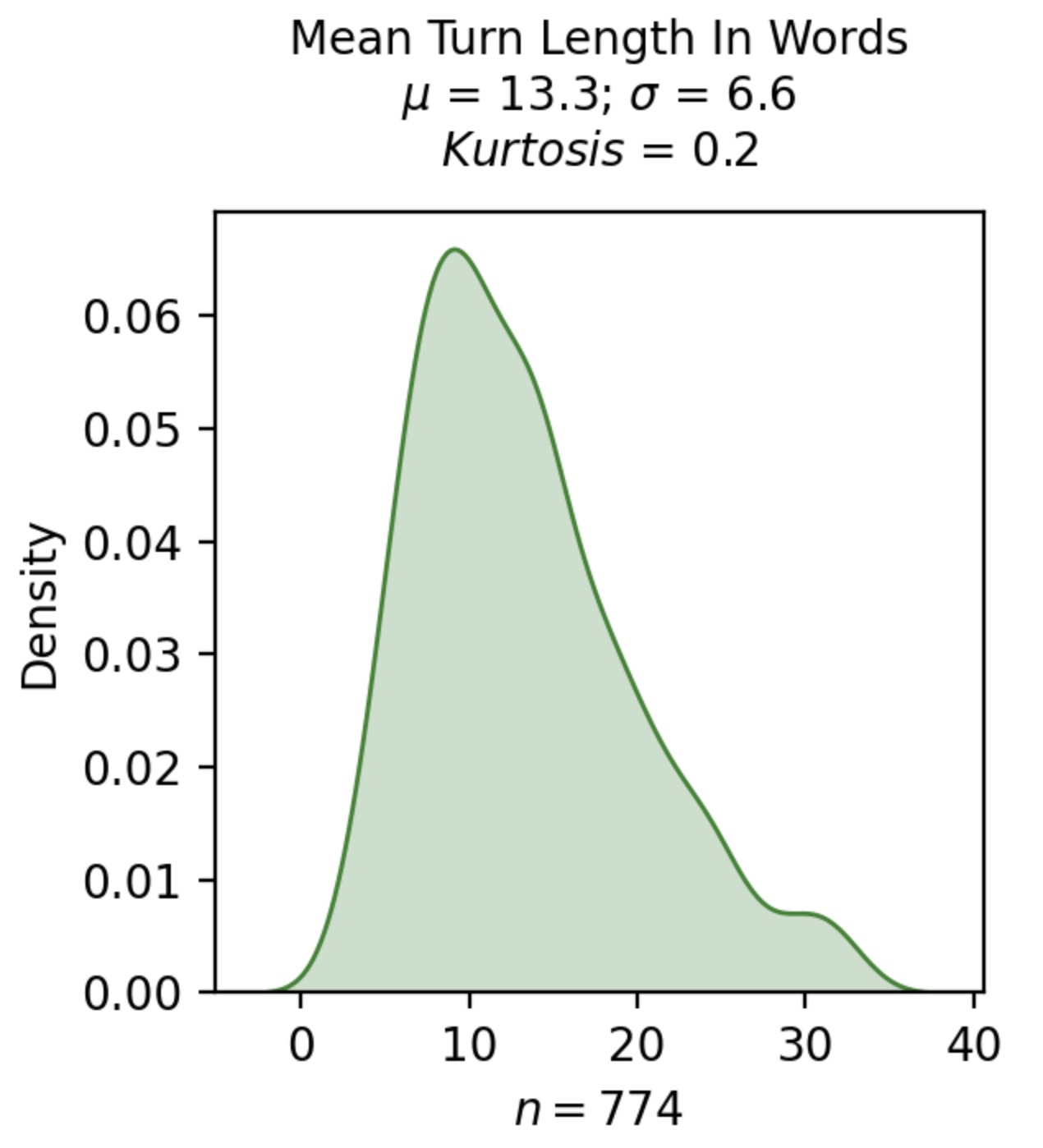 | 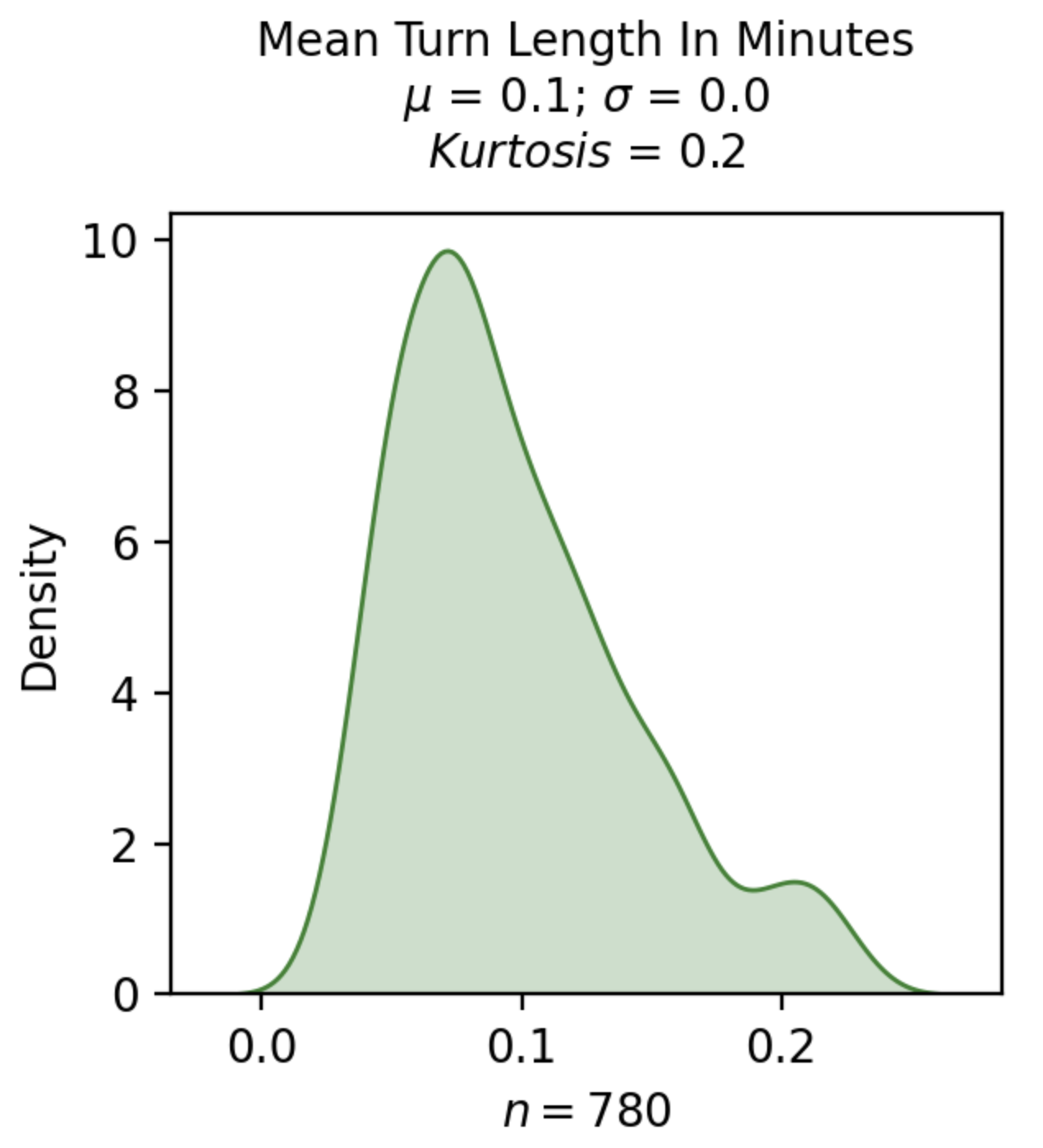 |
| --- | --- | --- | --- |
| 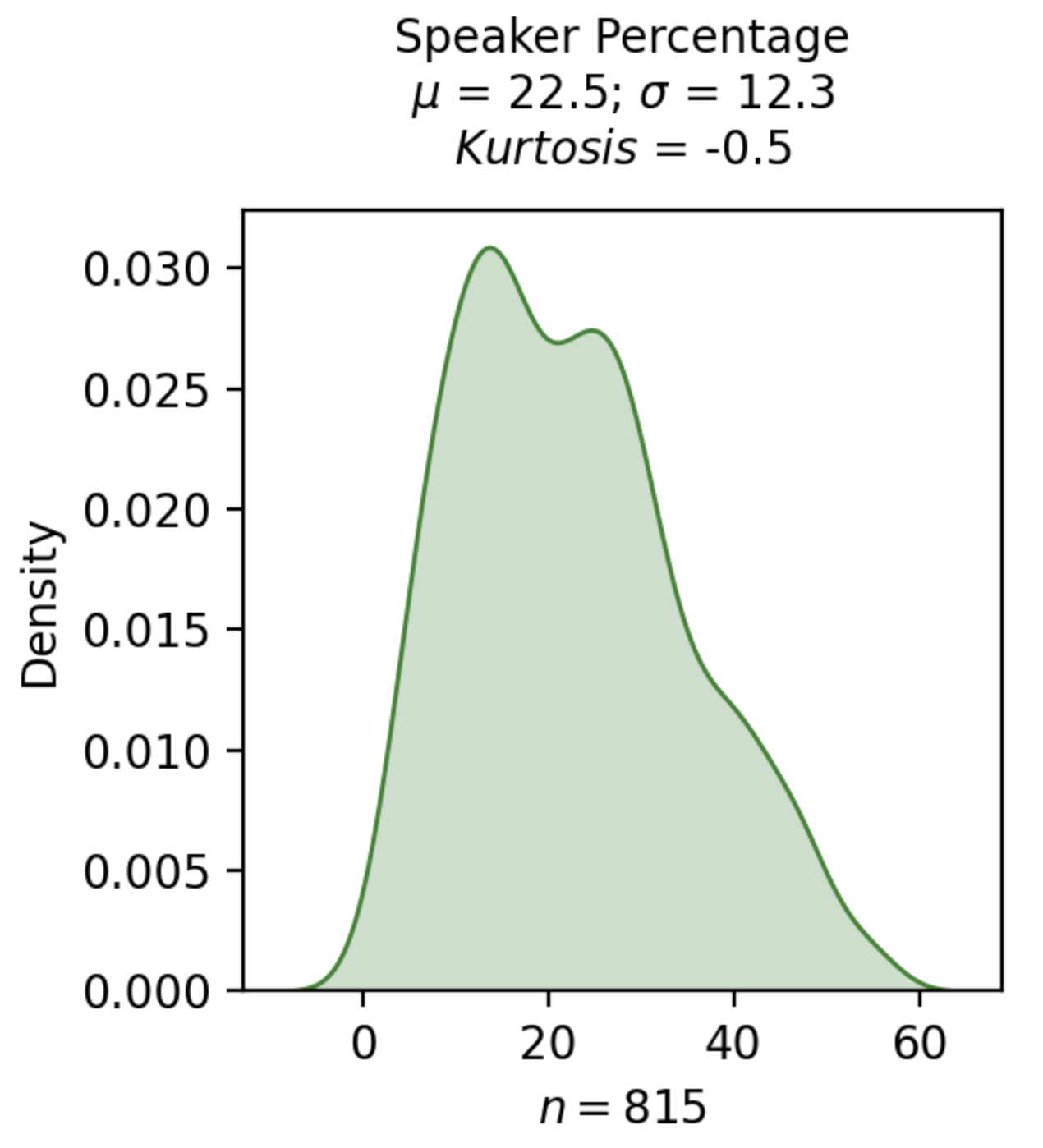 | 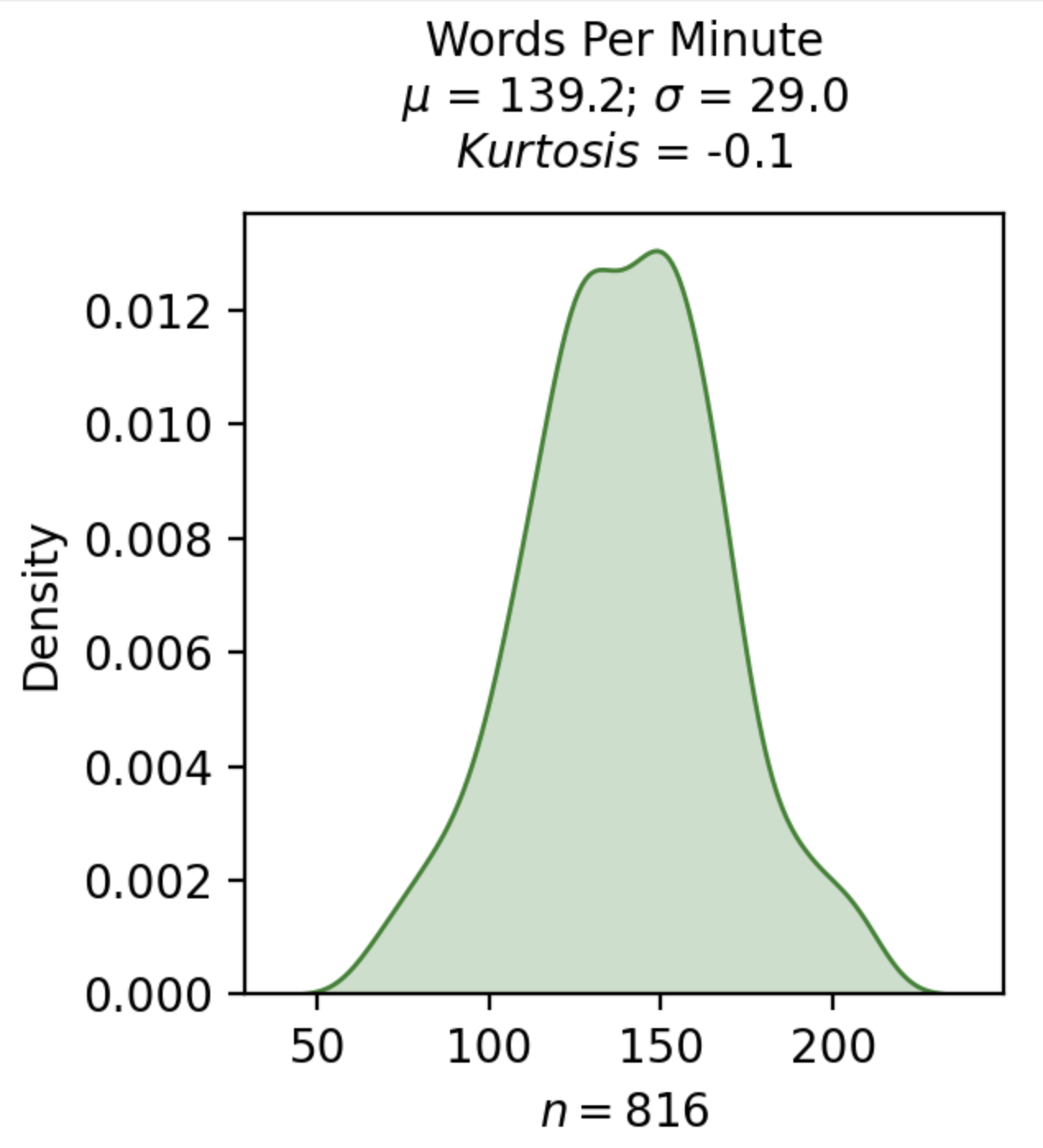 | 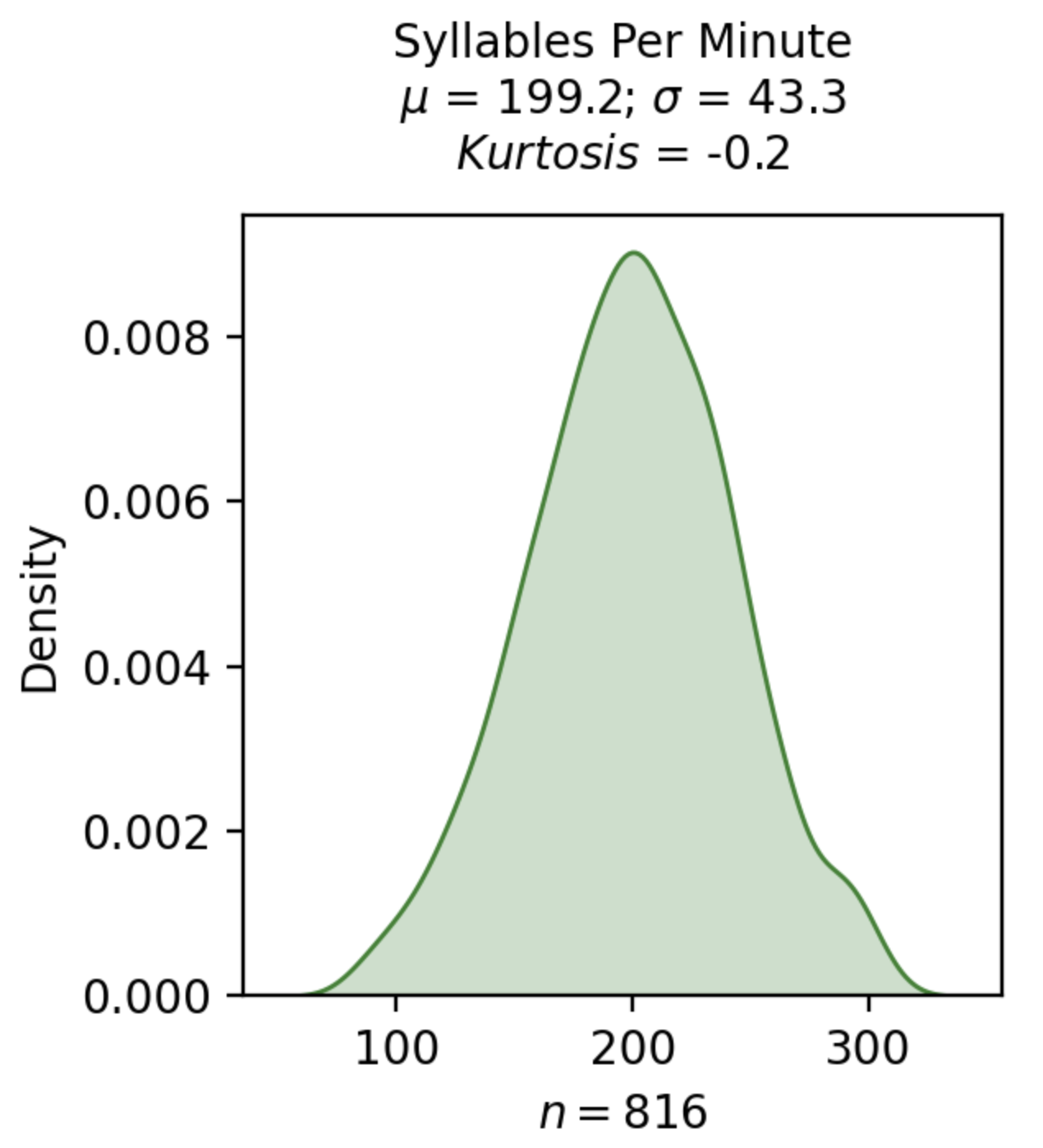 | 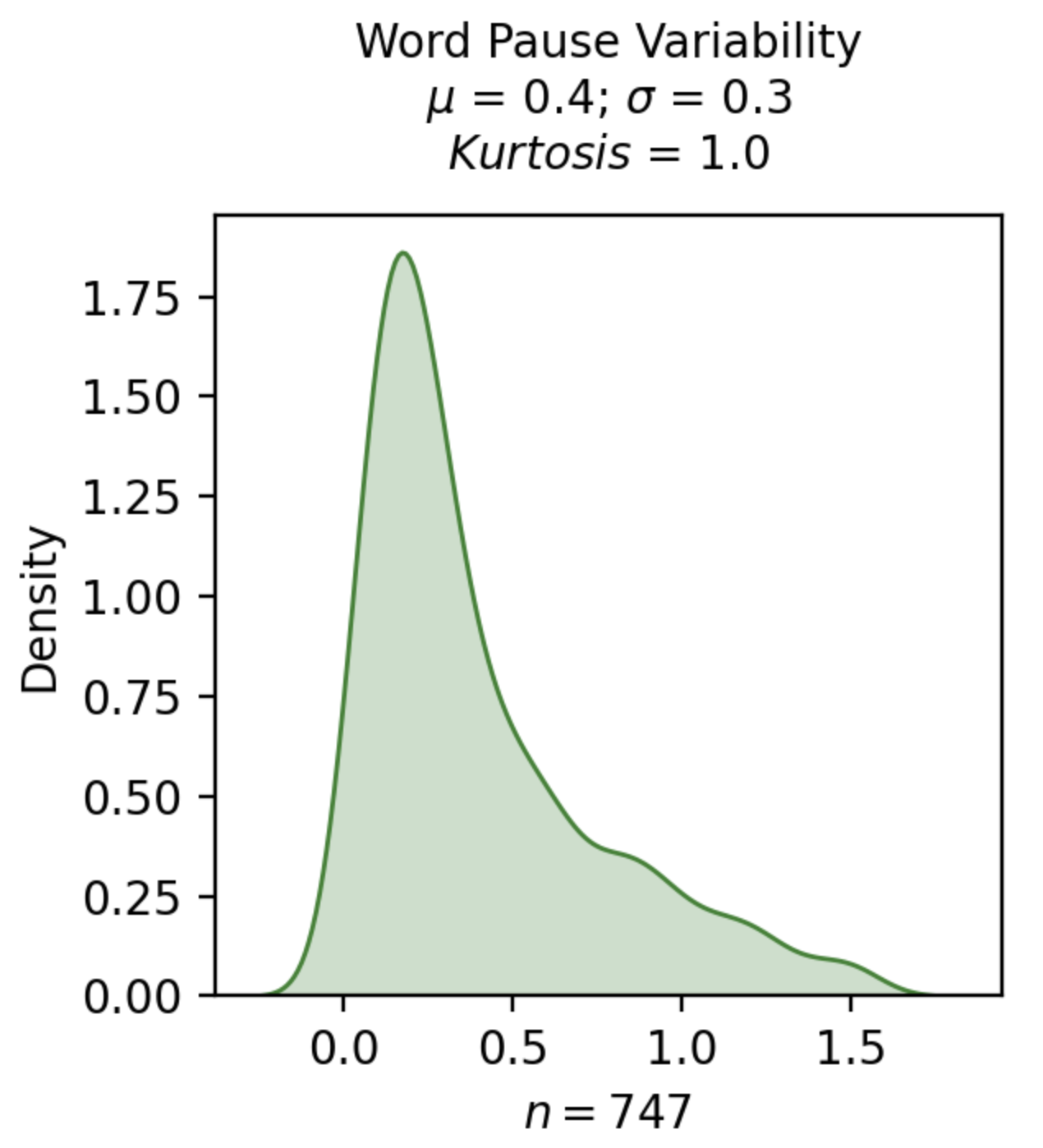 |
| 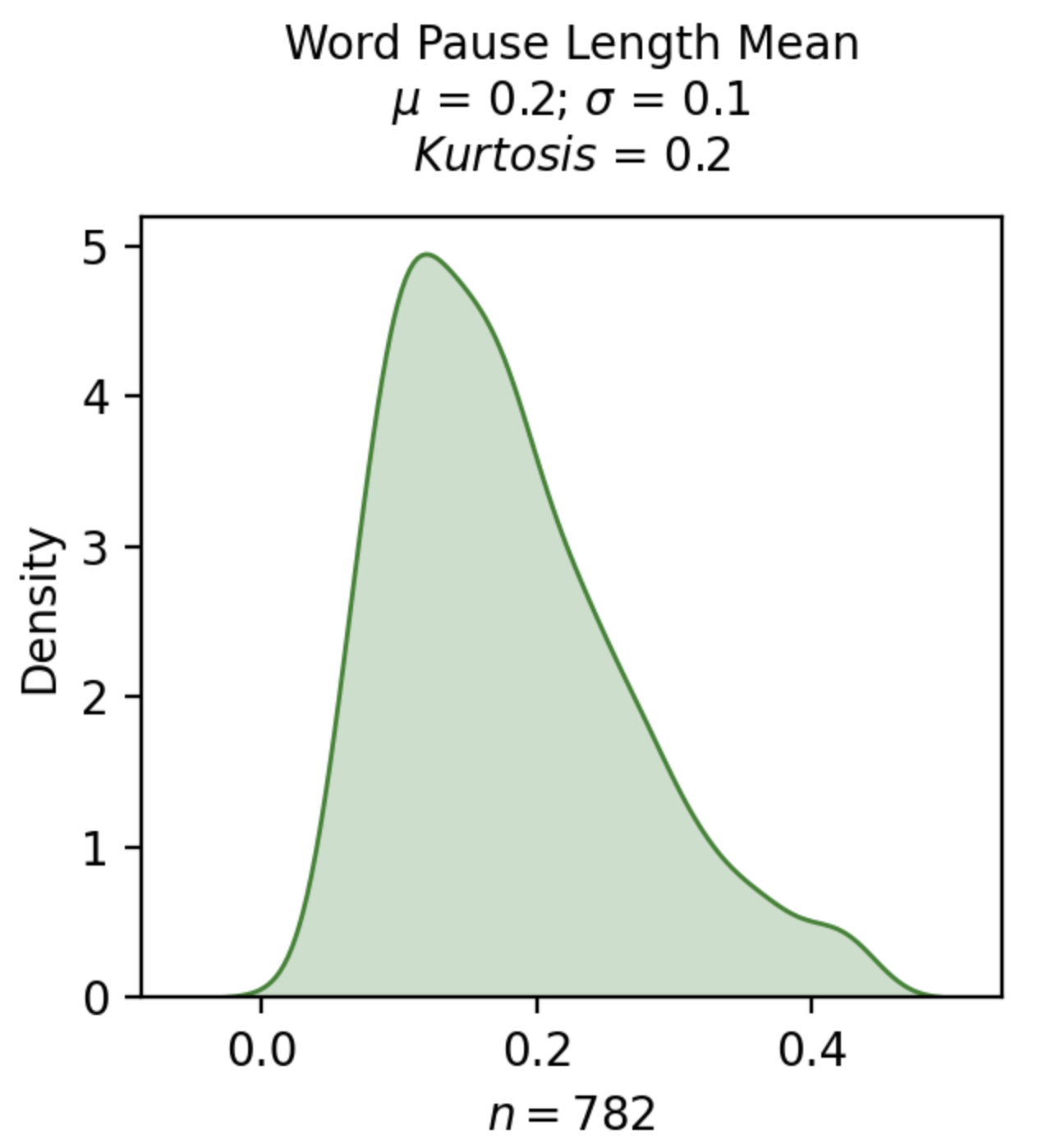 | 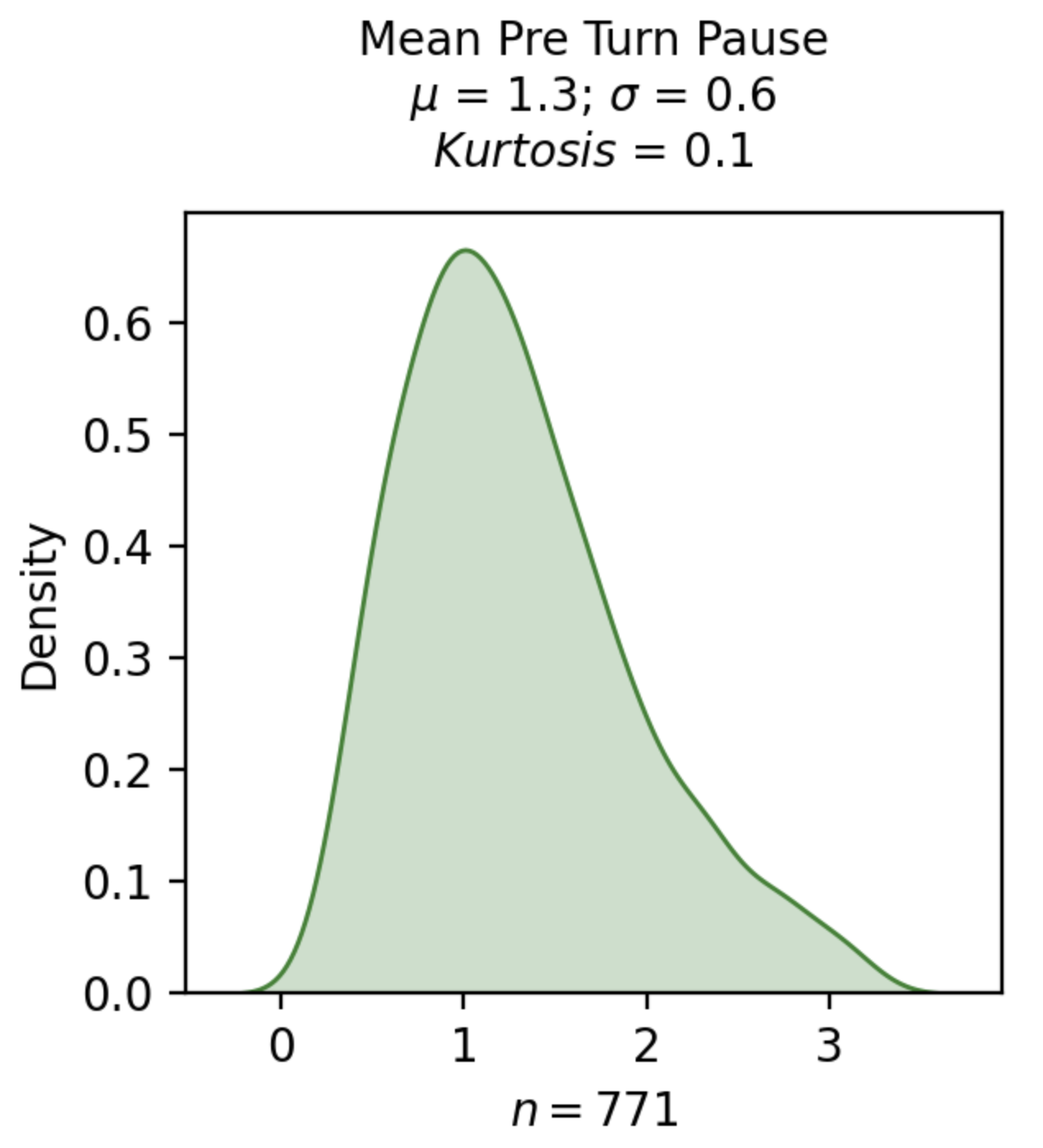 | 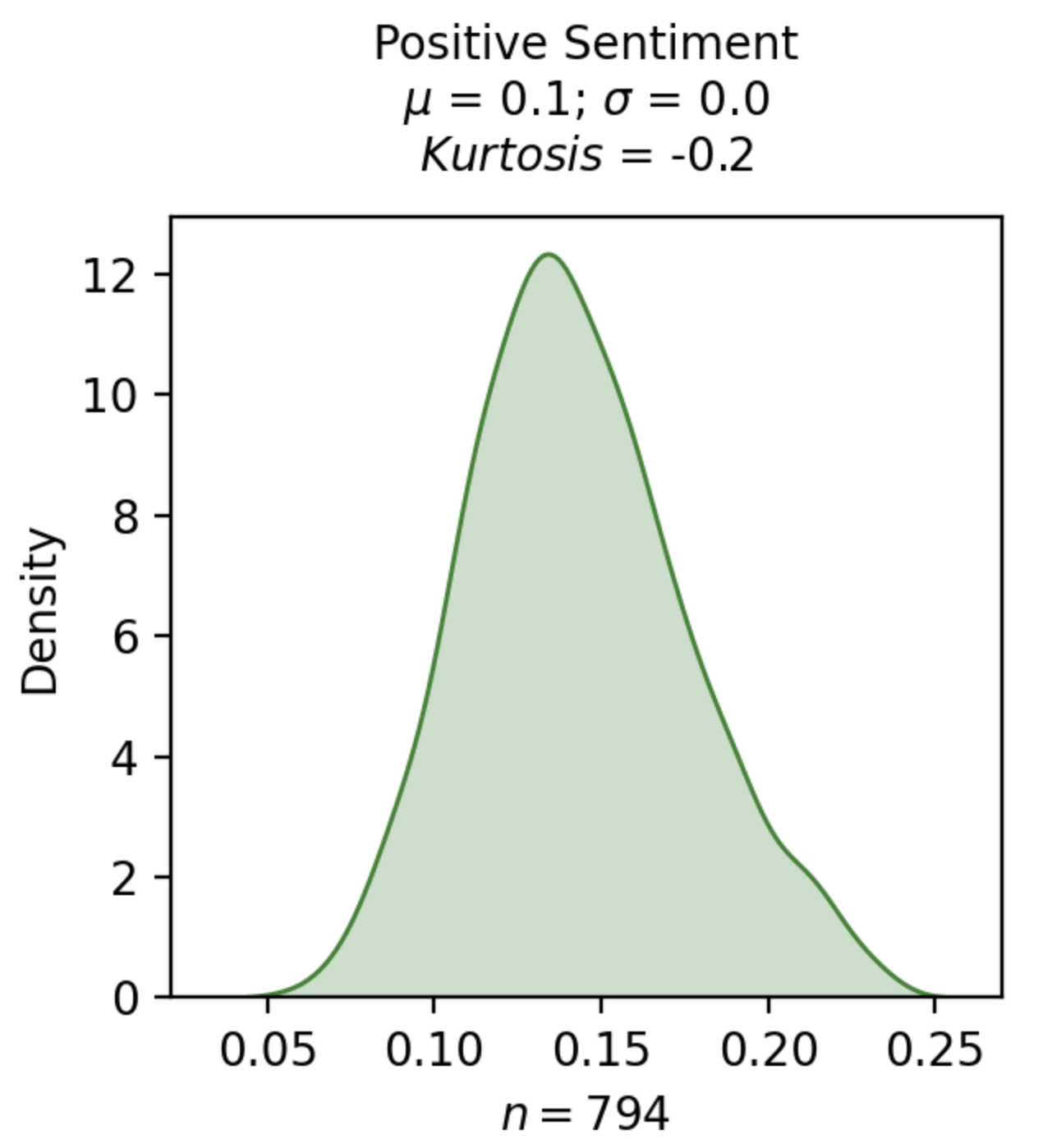 | 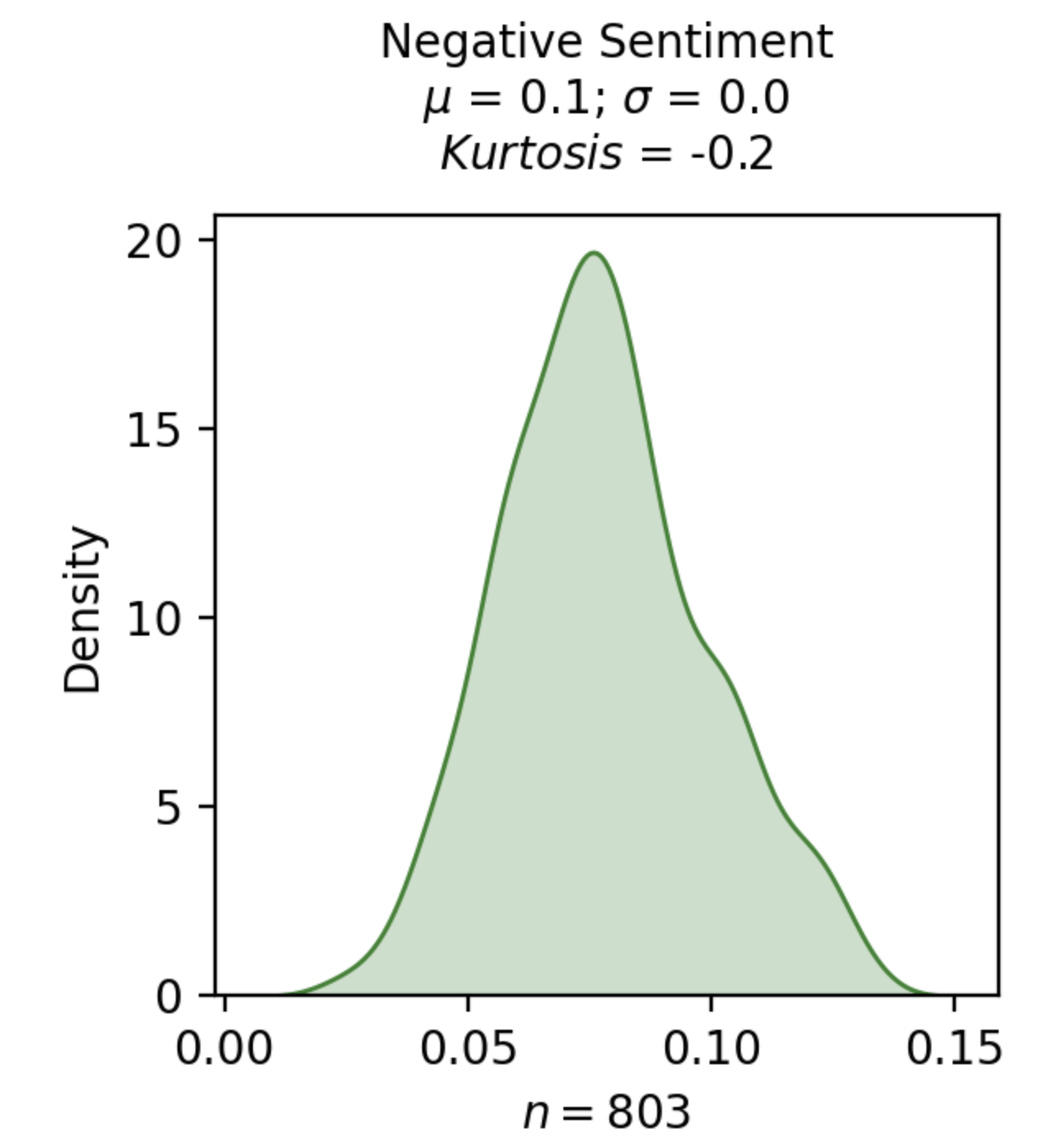 |
|  |  |  |  |

**Supplementary Figure 2.** Effect of **(a)** age, **(b)** sex, and **(c)** race on speech characteristics. Only comparisons that showed significant differences based on age, sex, or race are shown.

| **(a)** | 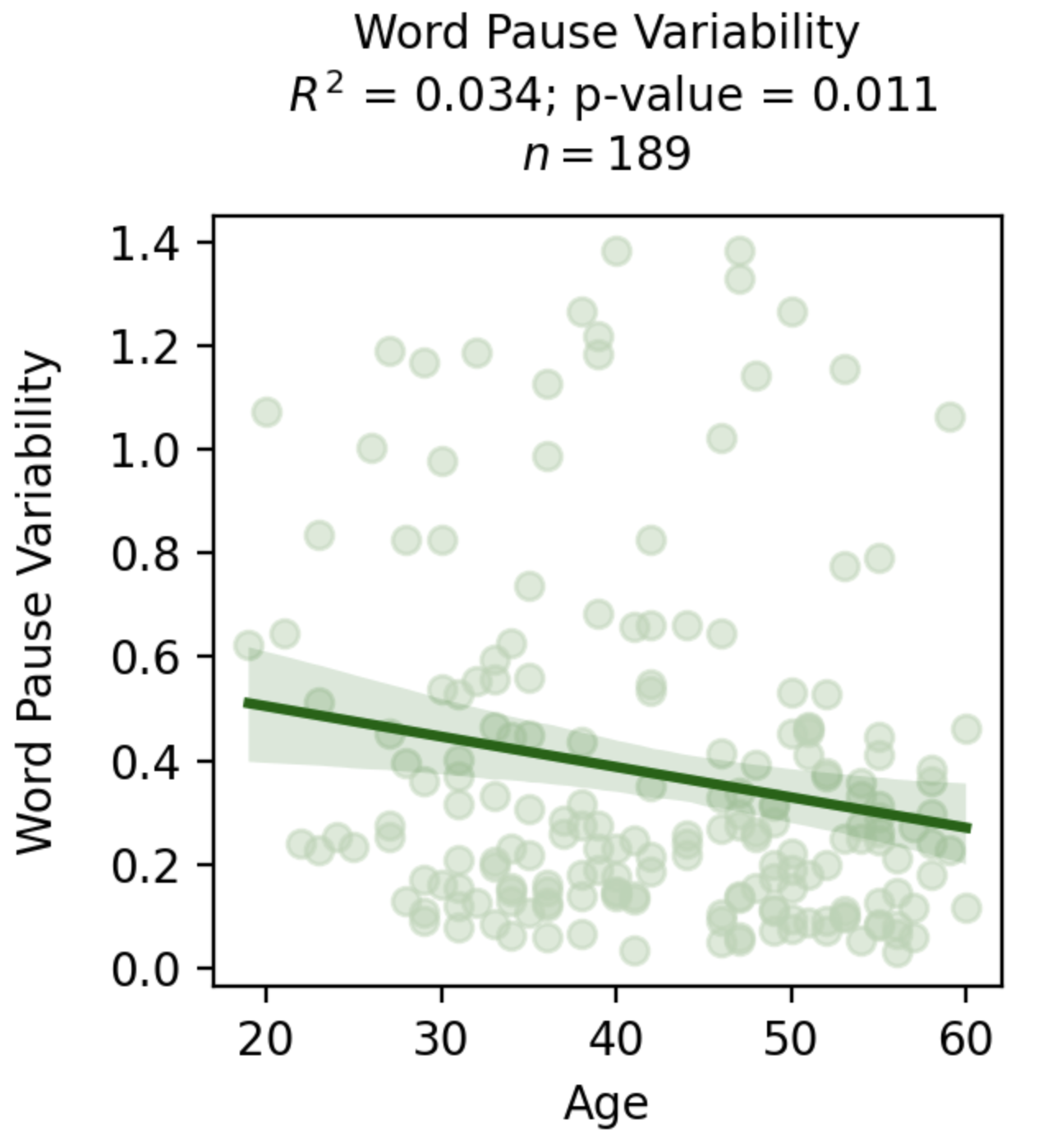 | 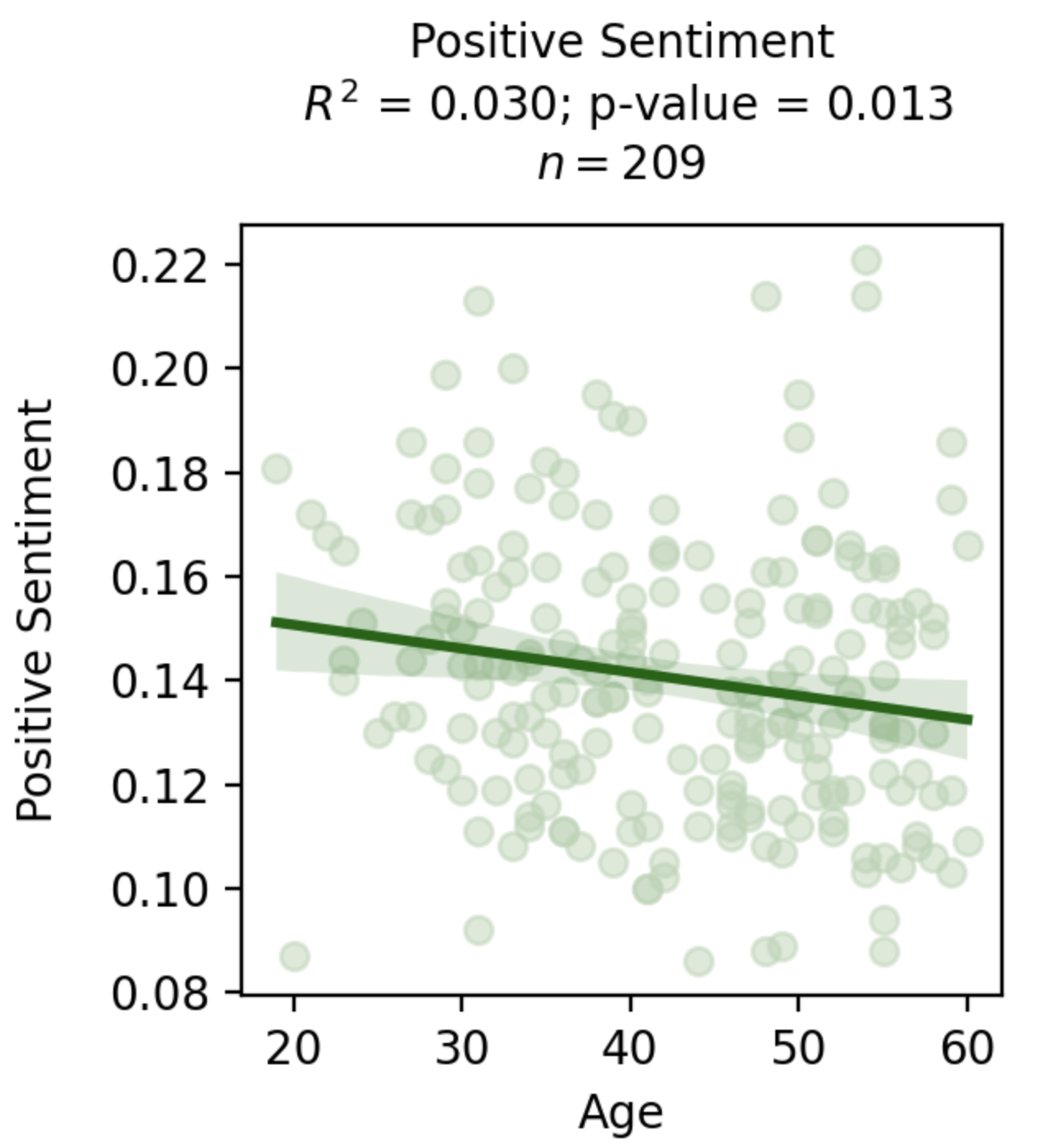 |  |
| --- | --- | --- | --- |
| **(b)** |  |  |  |
|  | 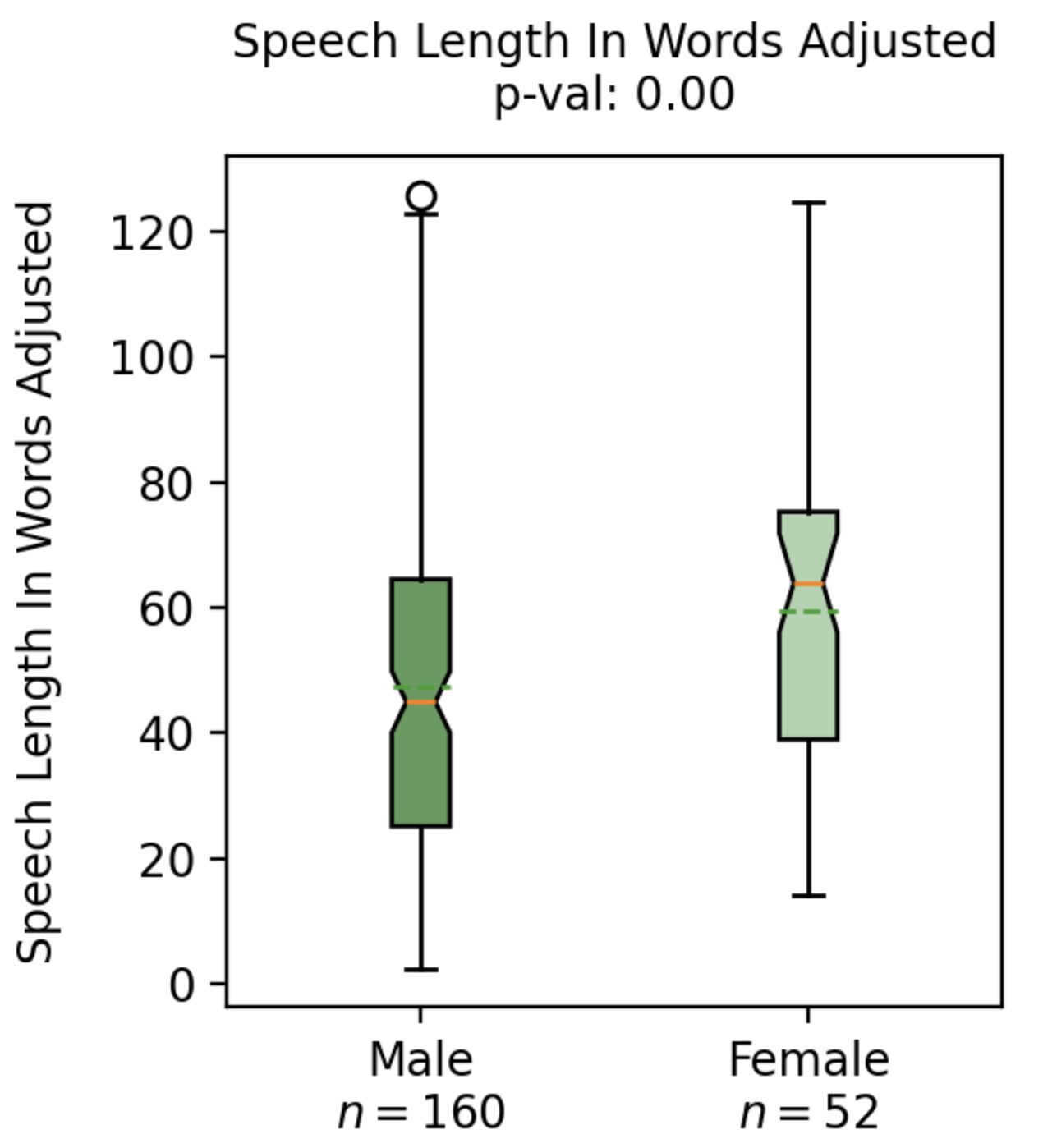  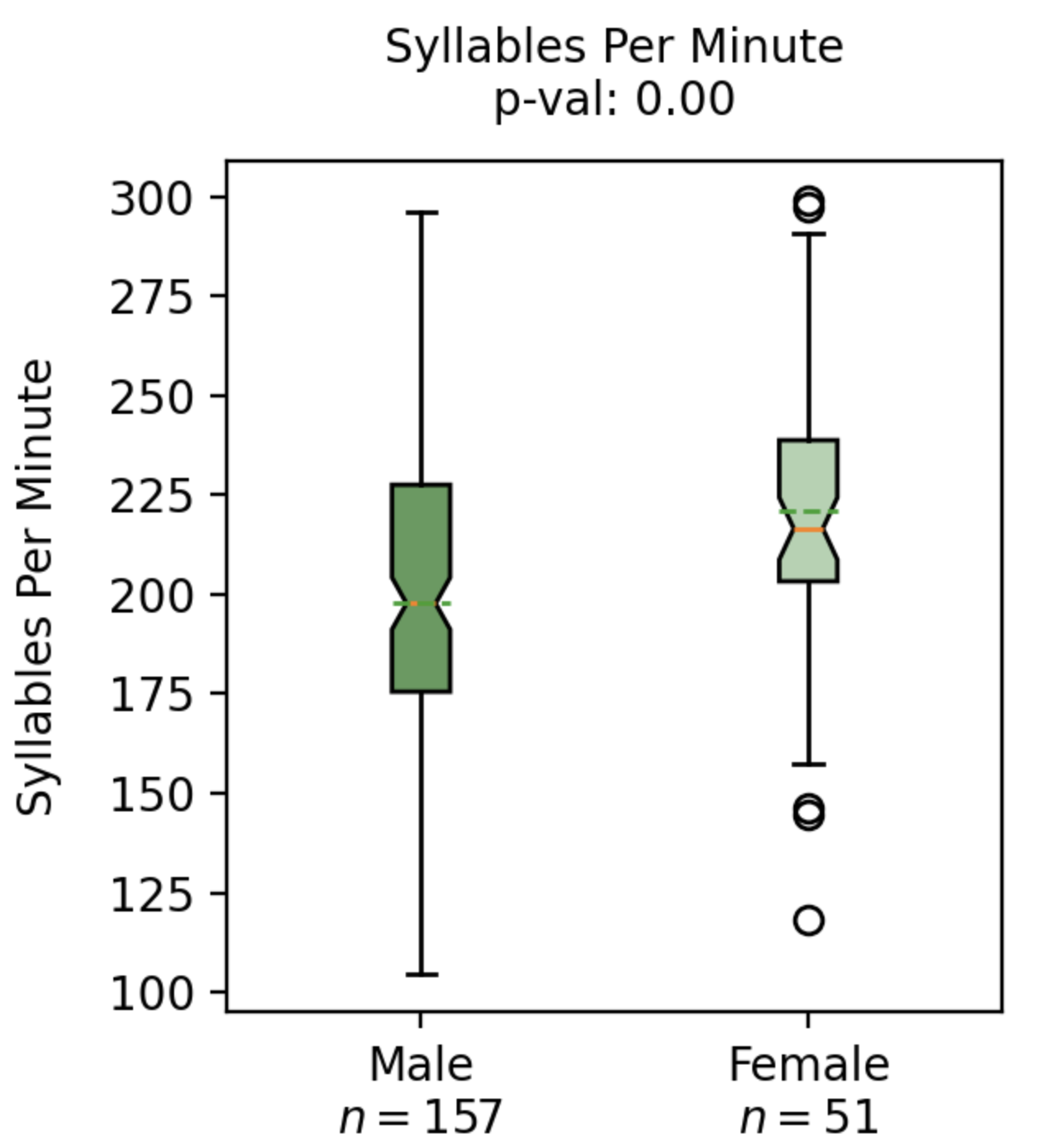 | 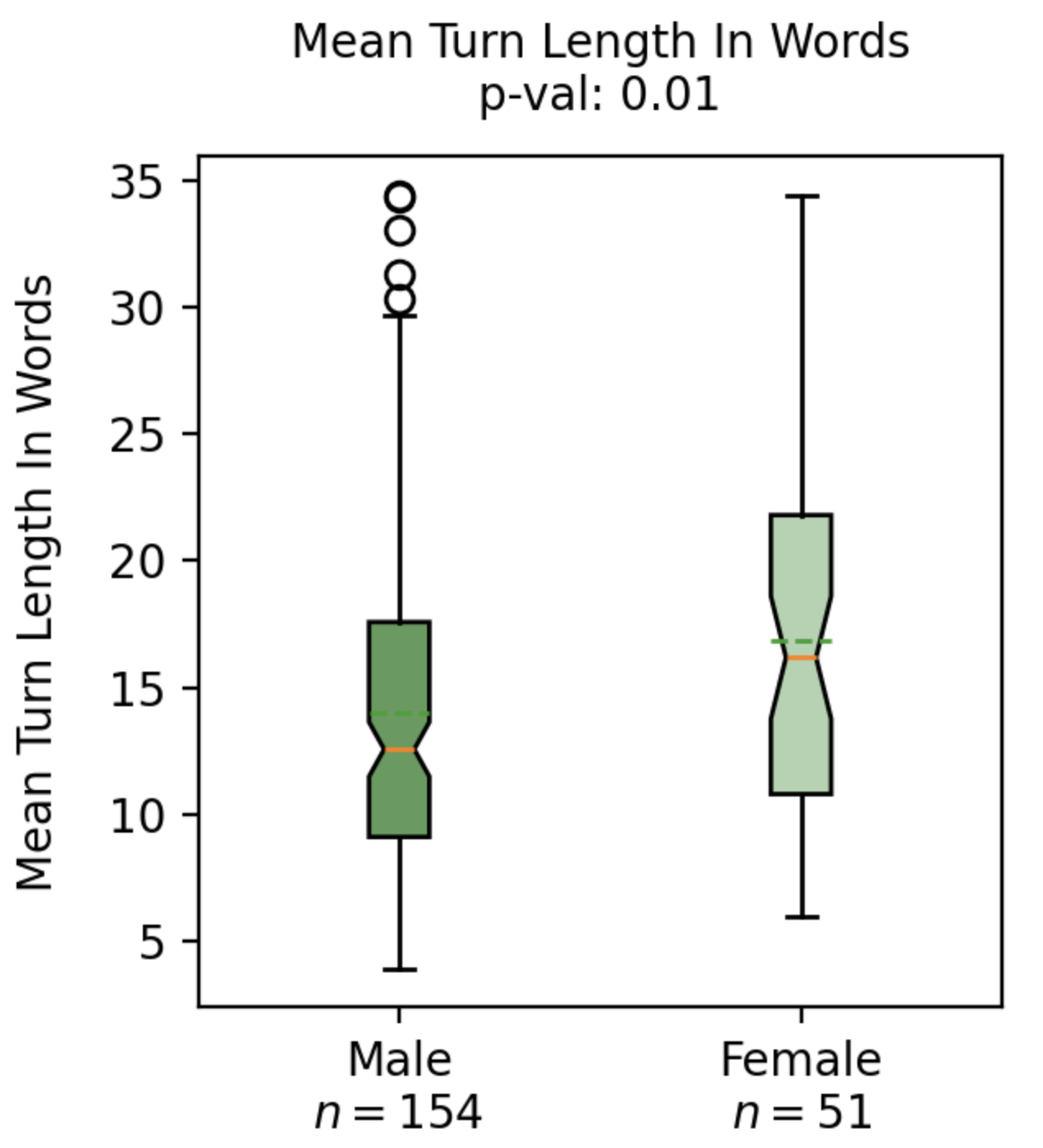  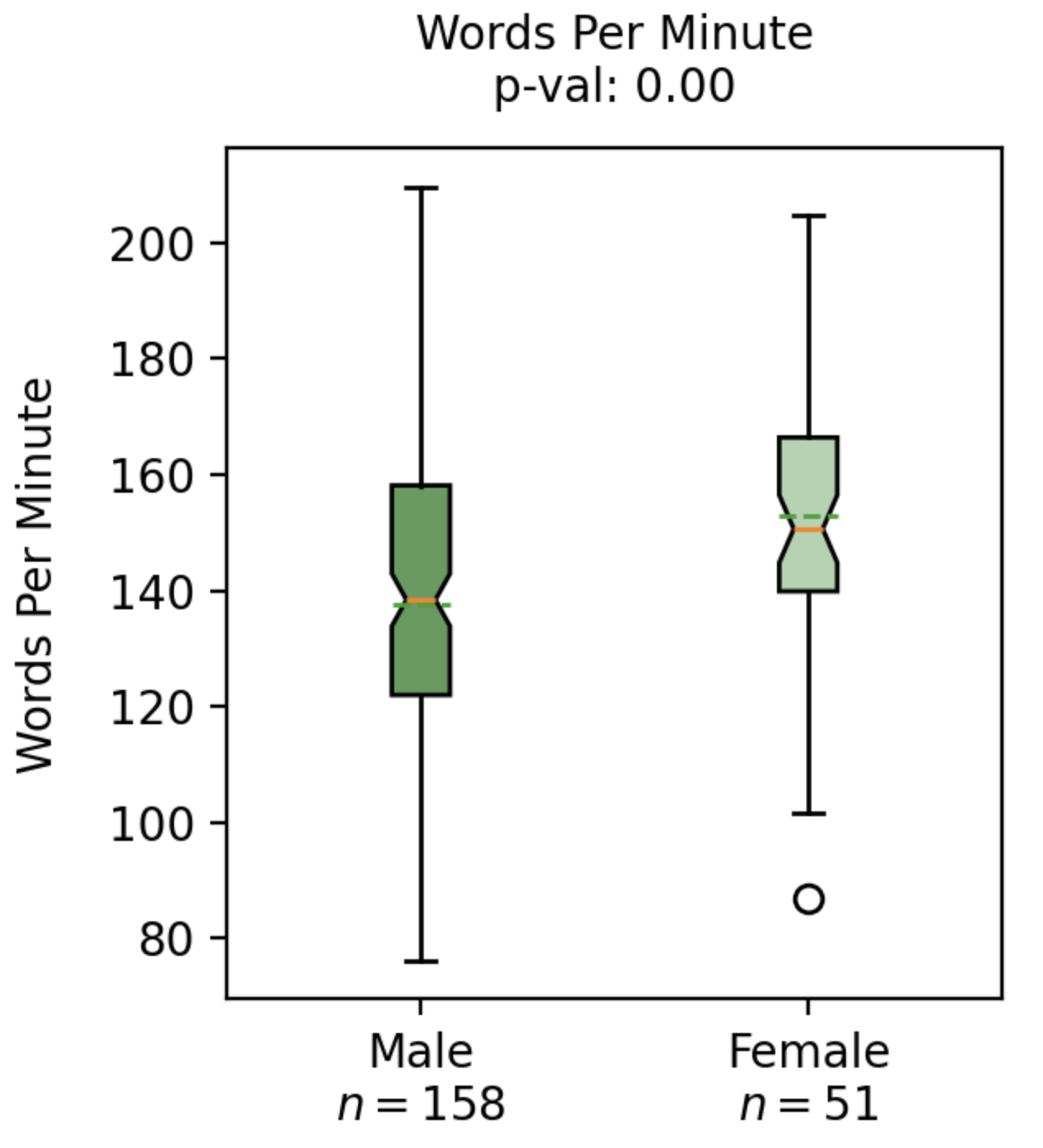 | 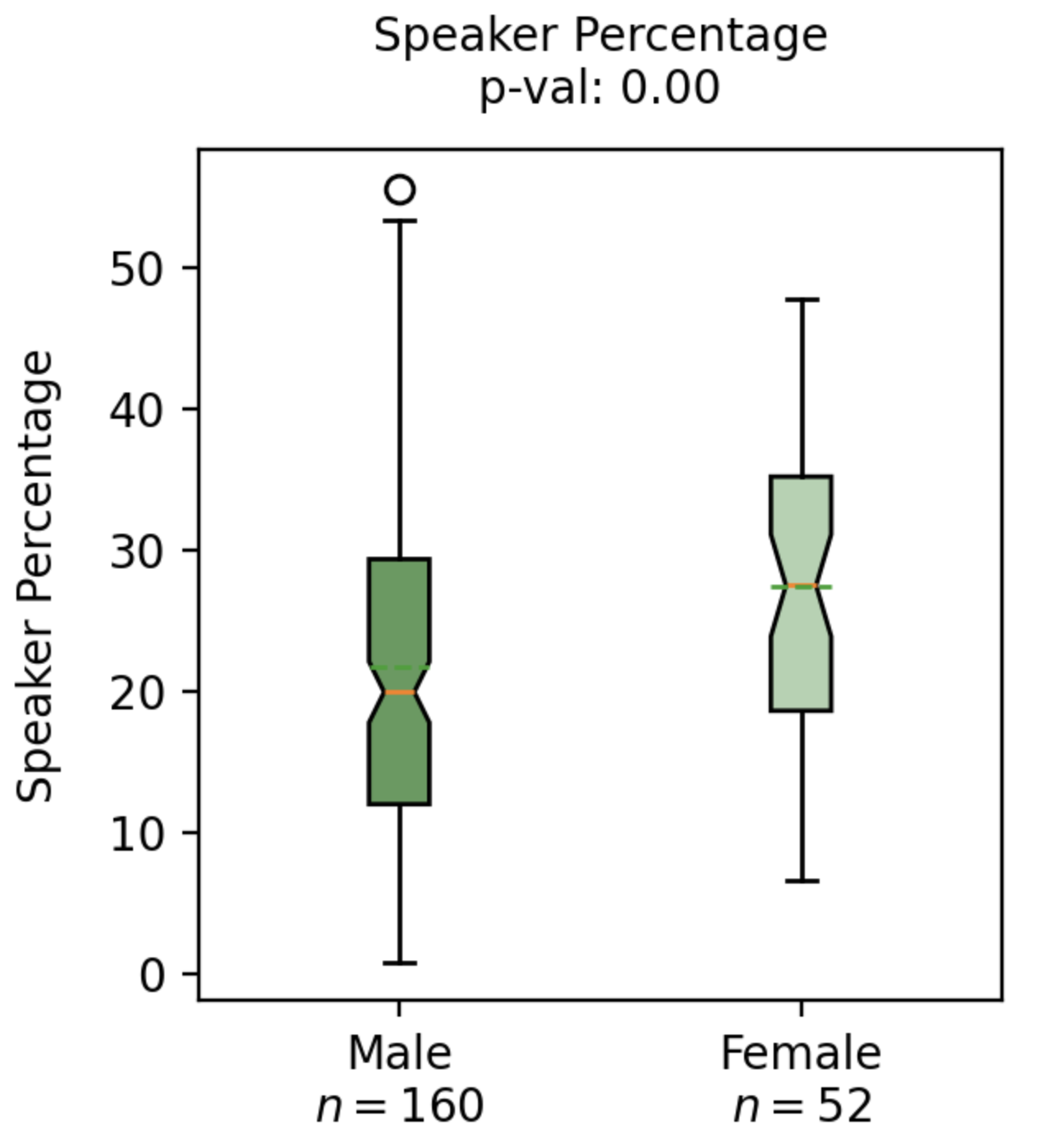 |
| **(c)** | 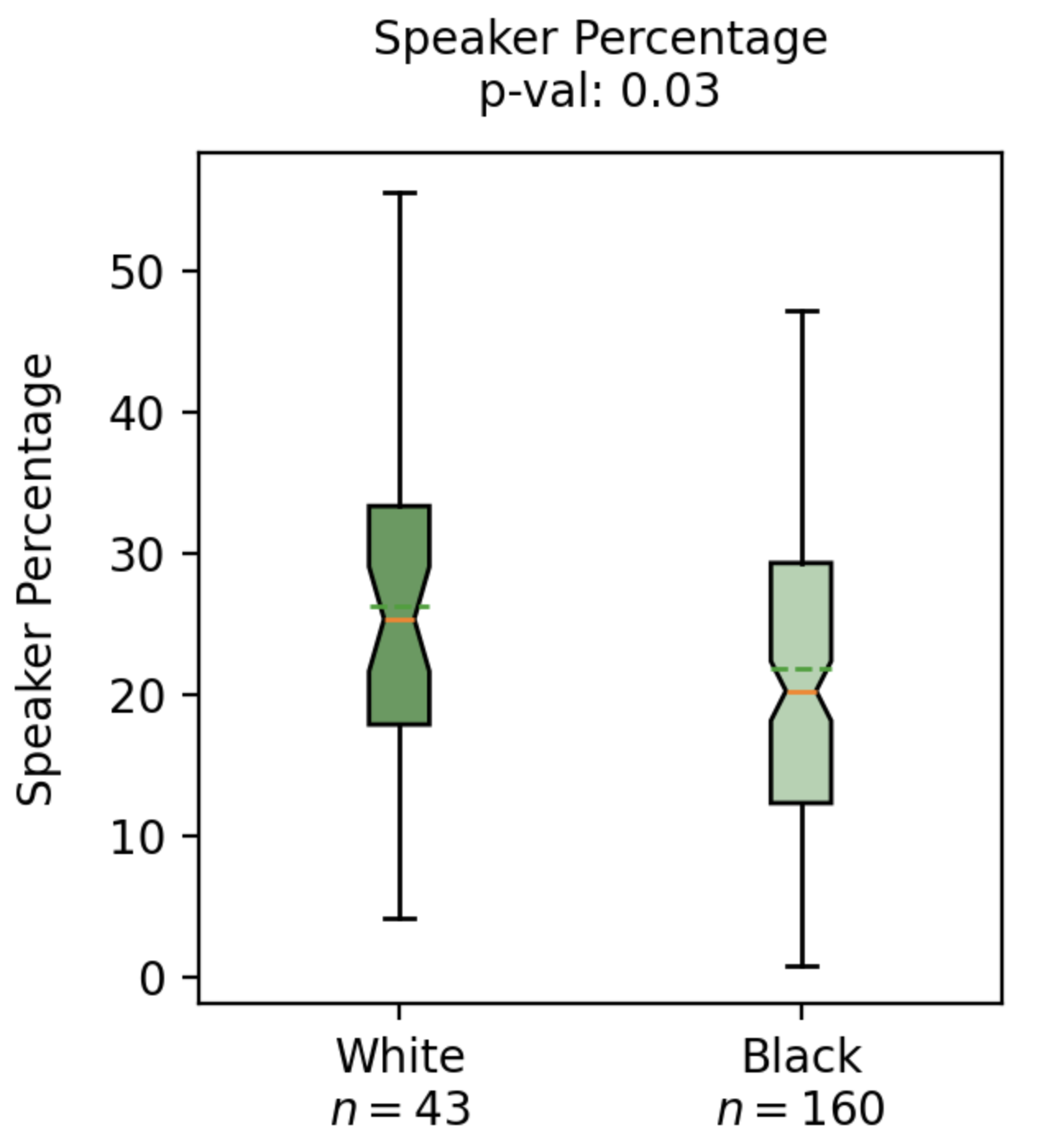 | 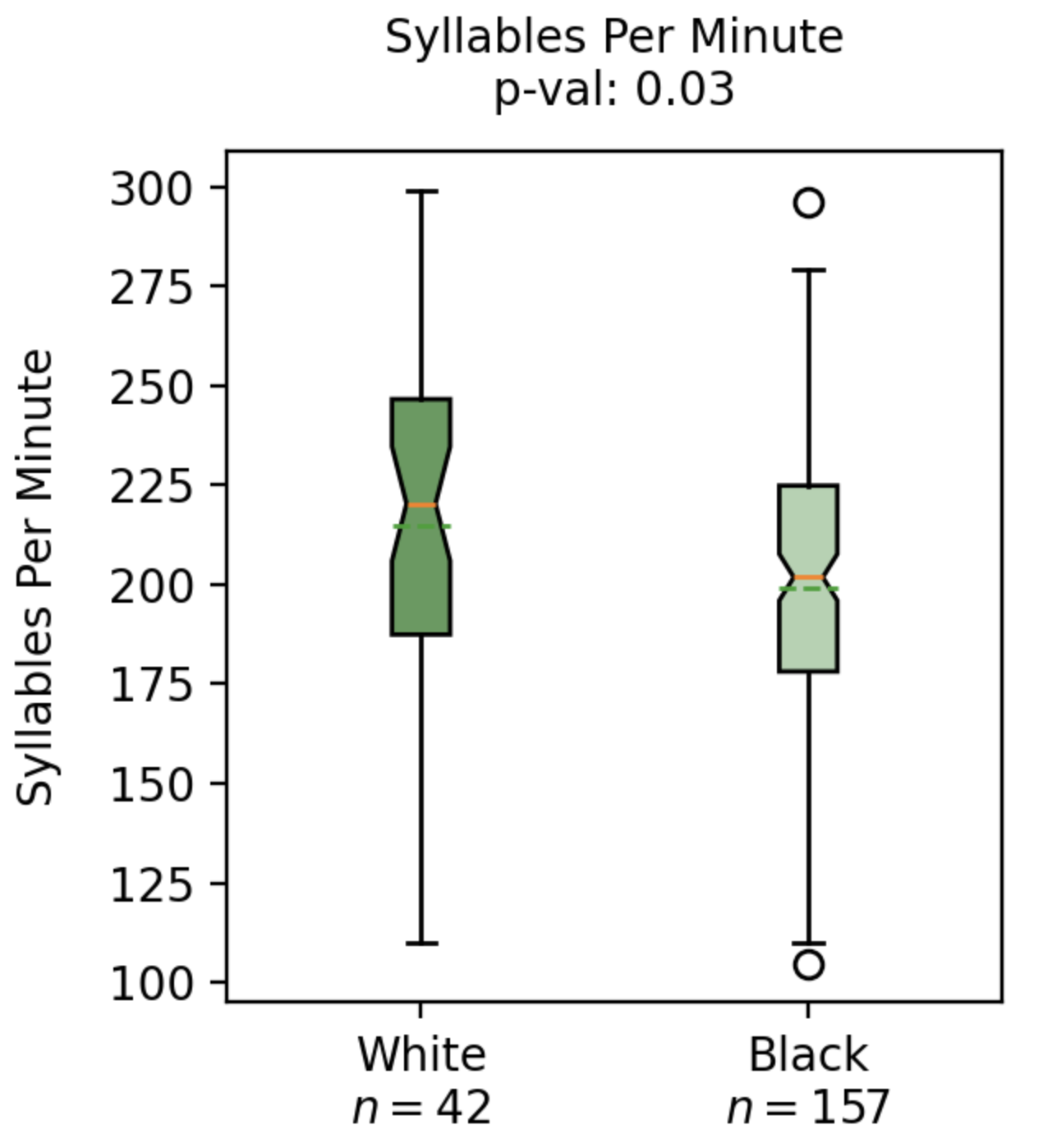 | 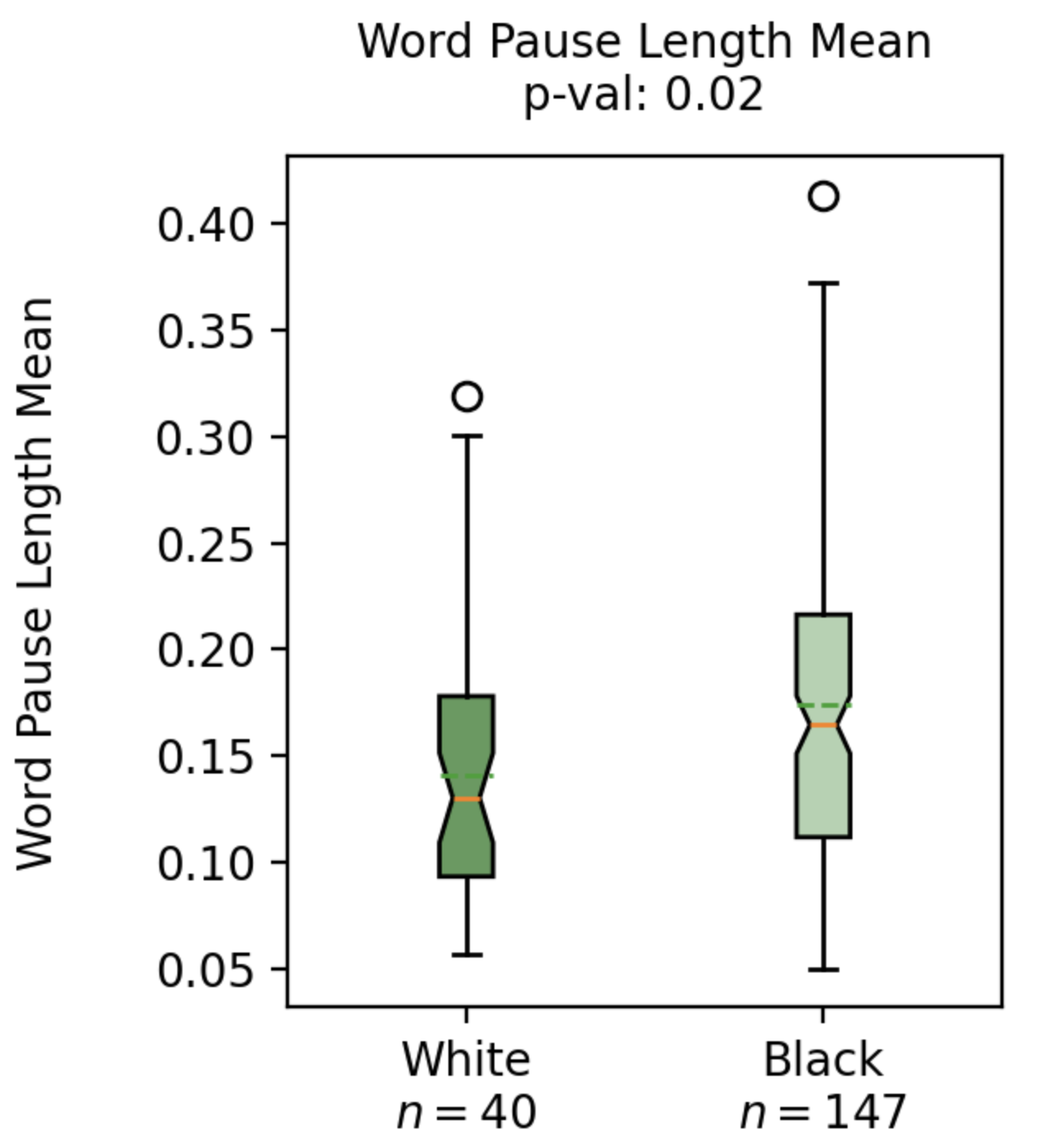 |

**Supplementary Figure 3.** Scatter plots showing significant relationships between PANSS-P and speech characteristics as assessed by multiple linear regressions. Statistics specifically for the speech characteristics from the multiple linear regressions are shown above the graph, where age, sex, and race were included as covariates. This analysis used averaged values from the screening and baseline visits.

| 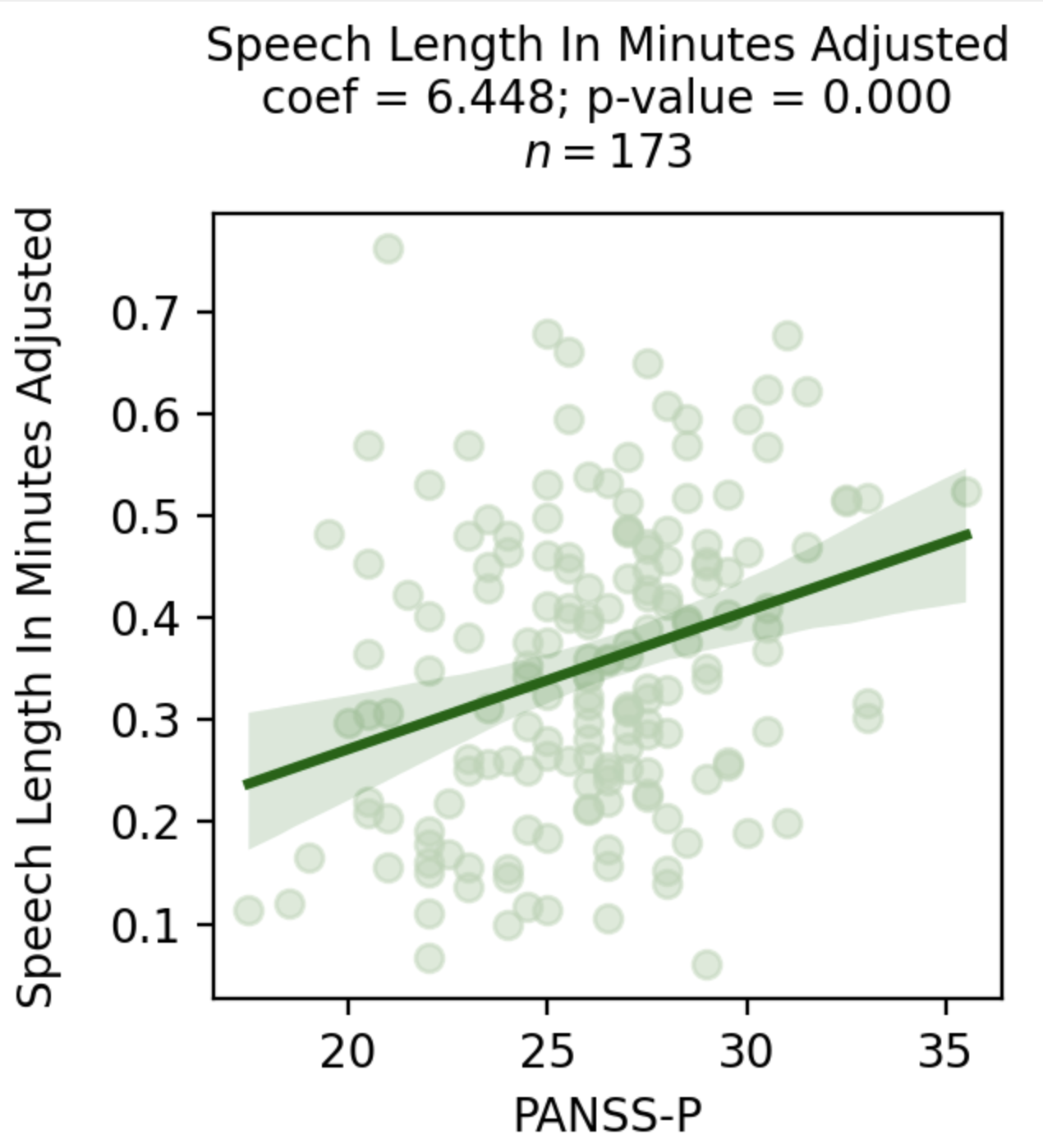 | 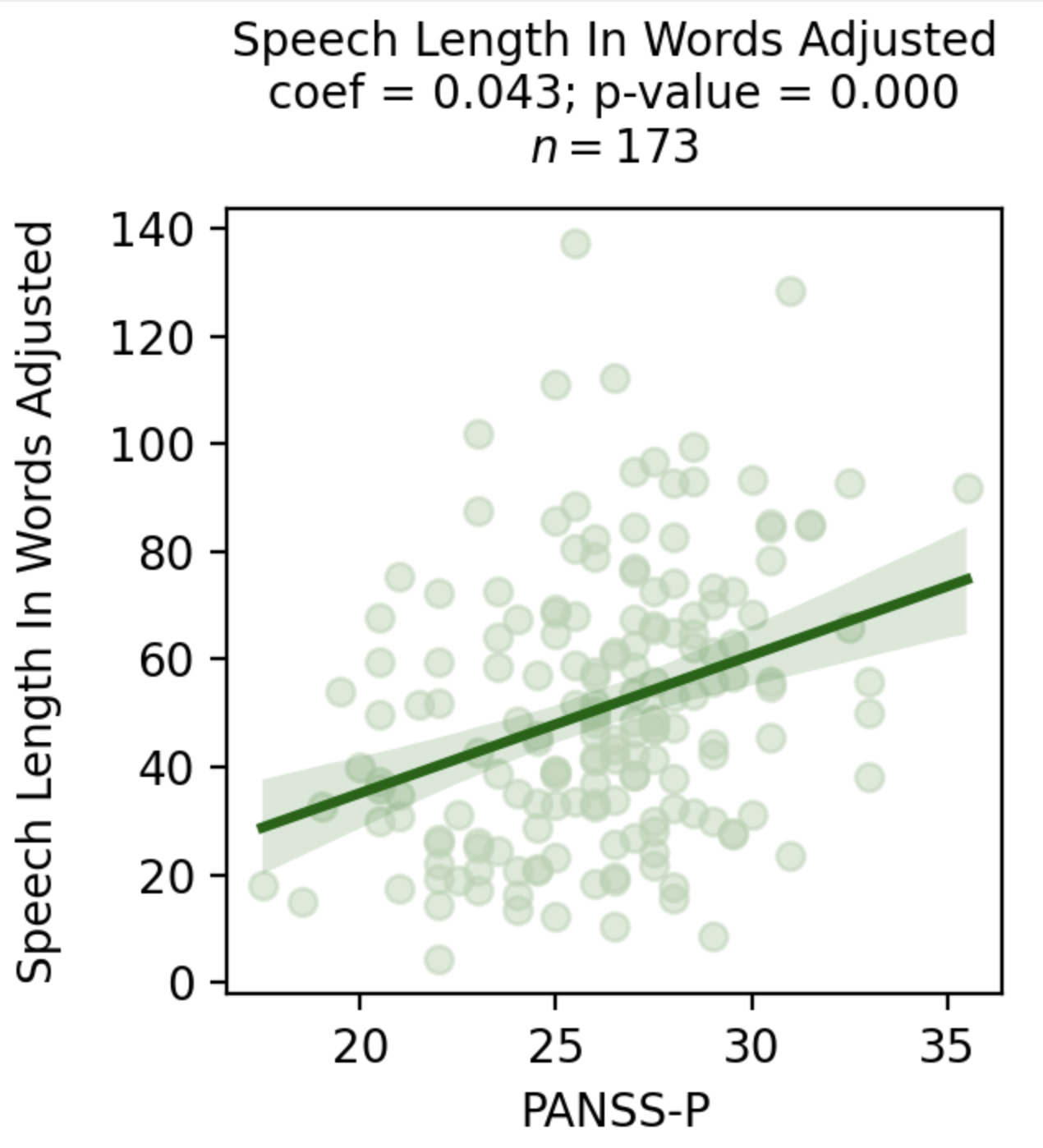 | 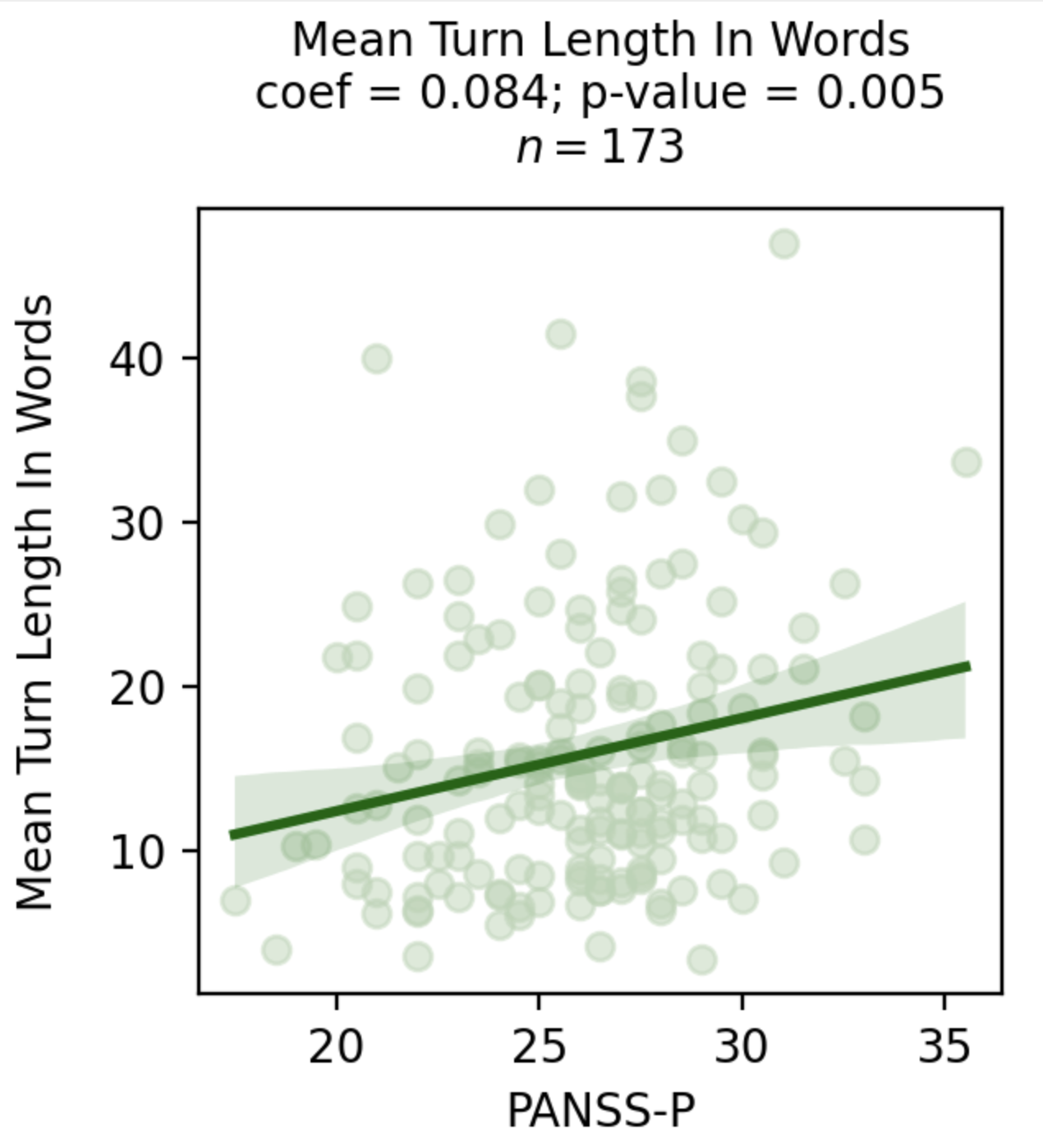 | 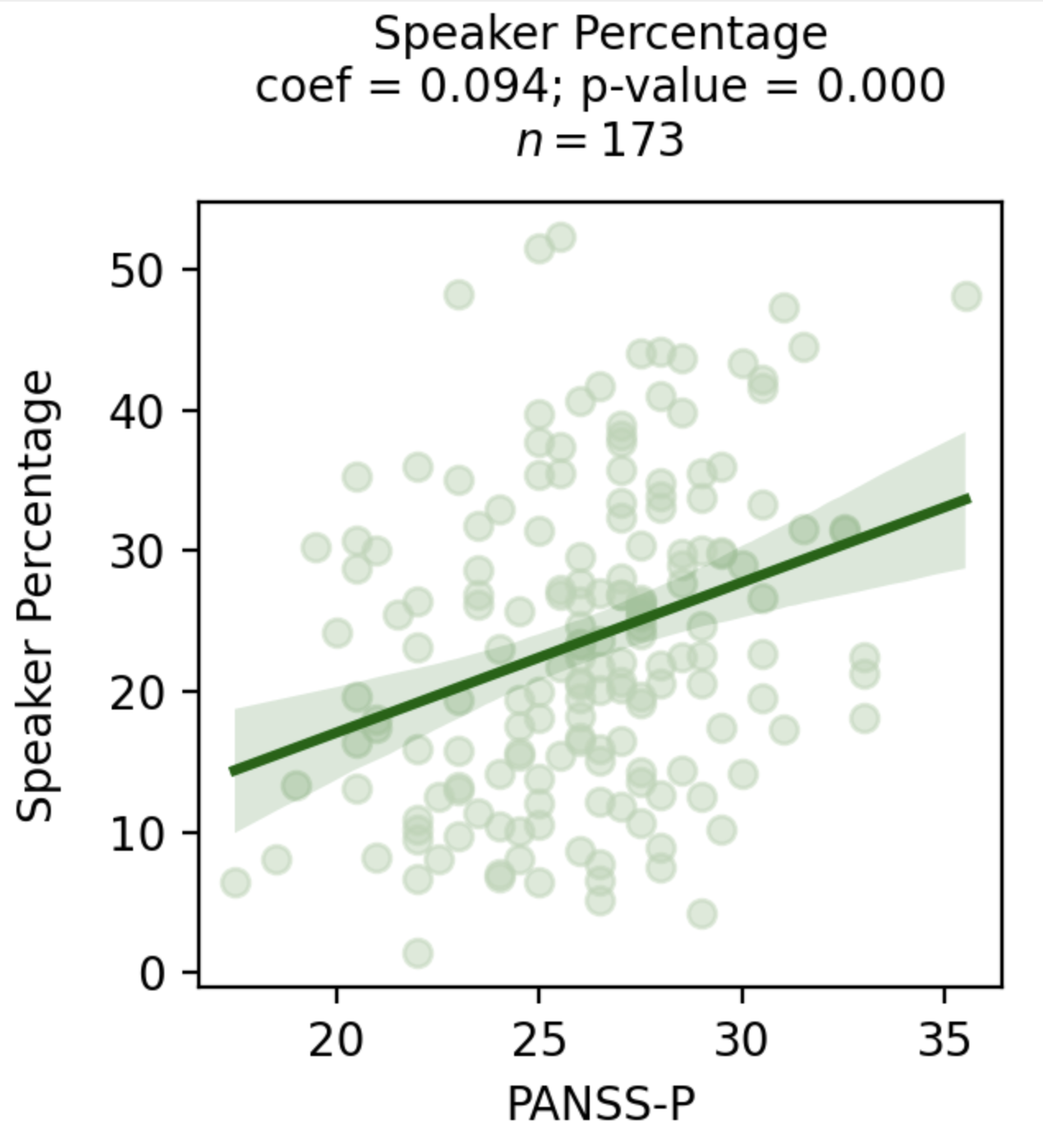 |
| --- | --- | --- | --- |
| 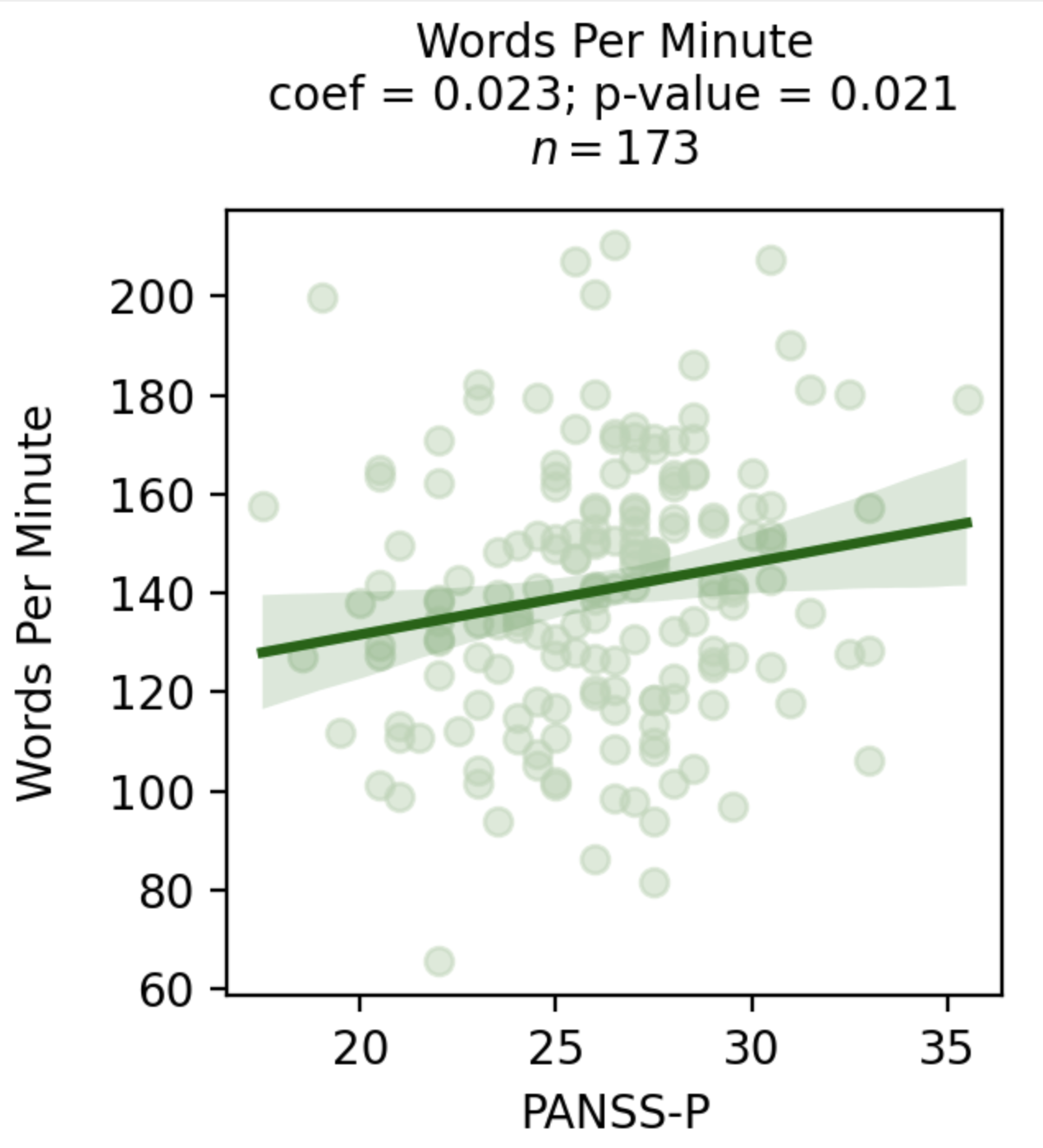 | 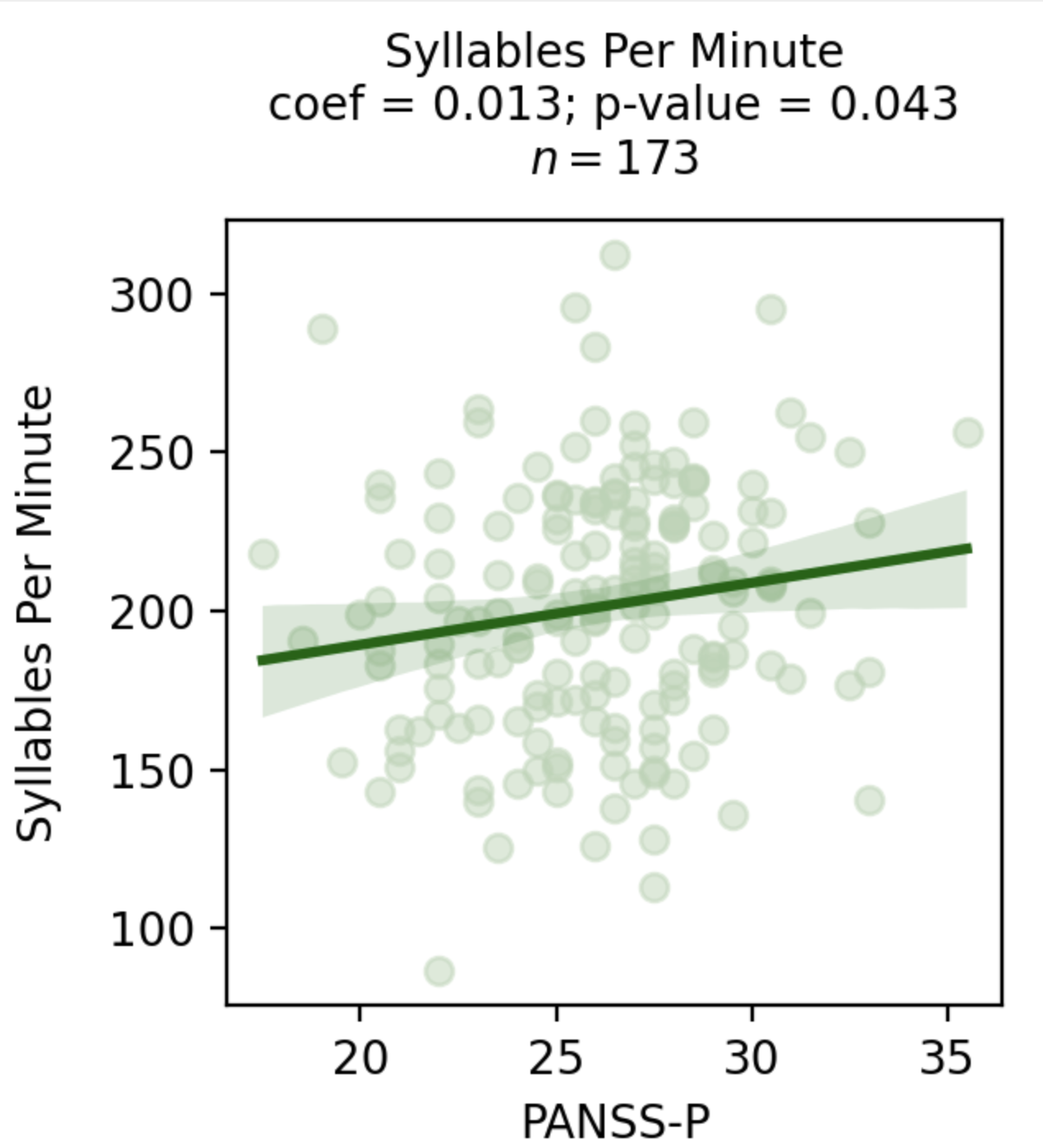 | 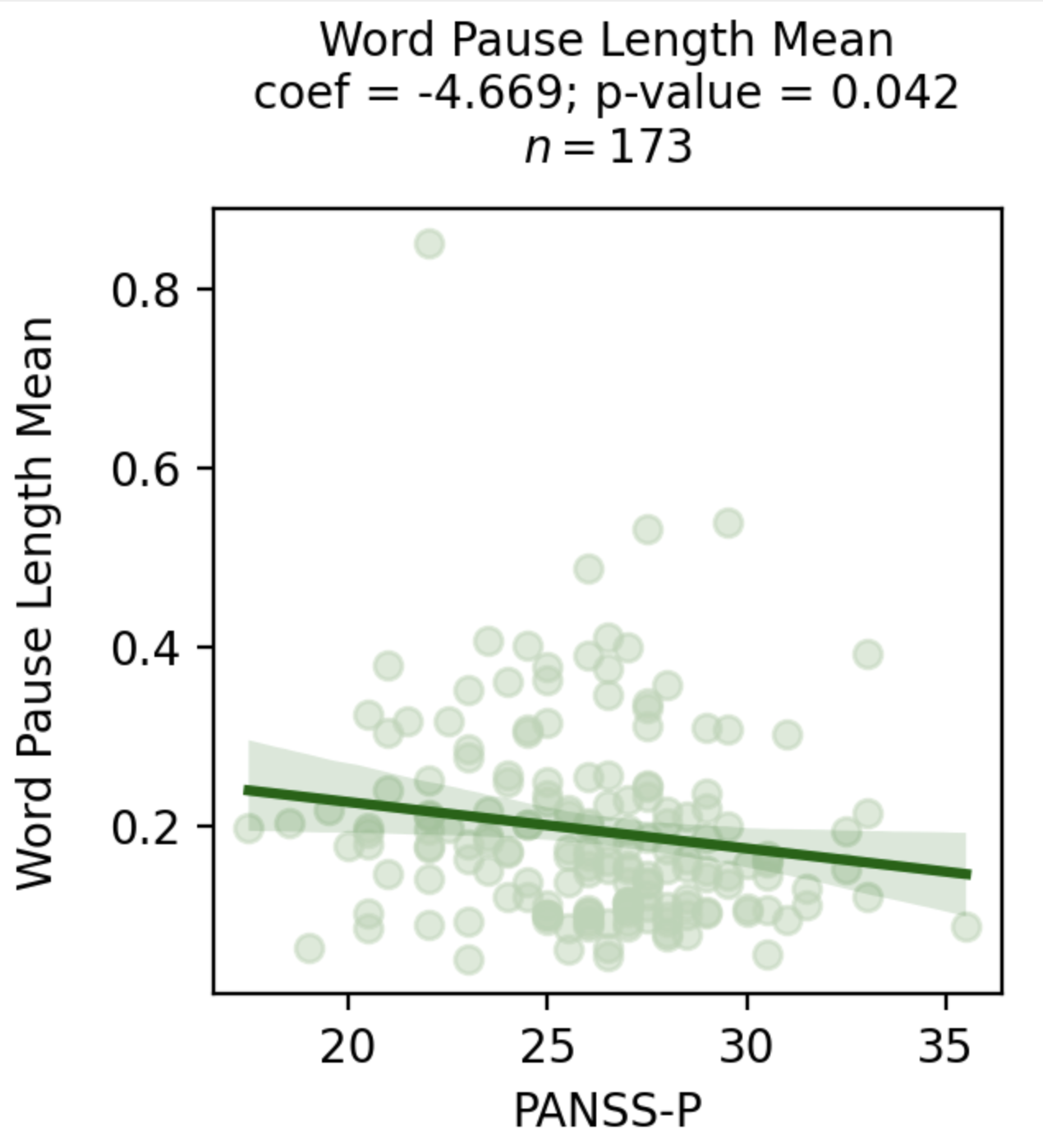 | 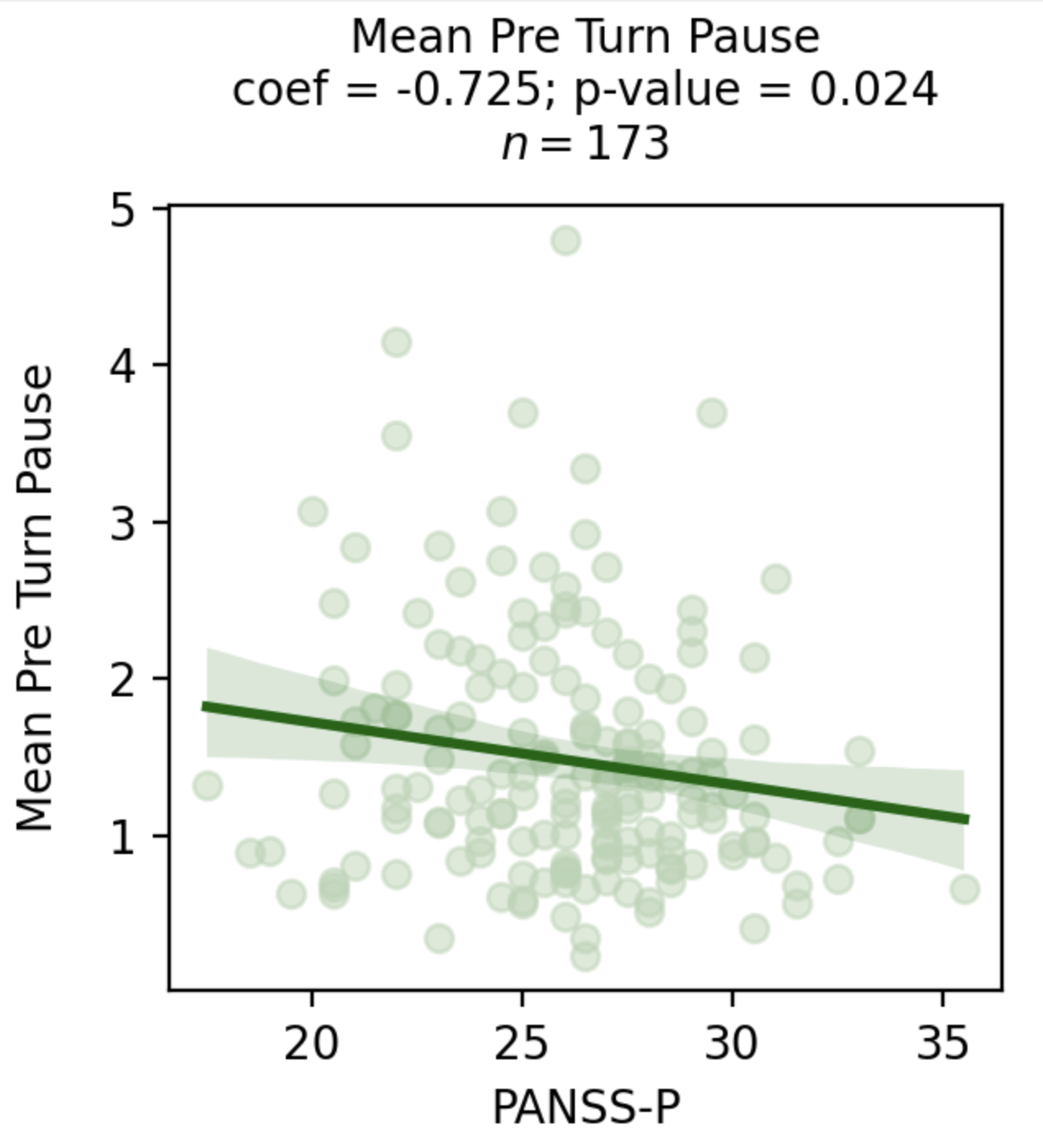 |
| 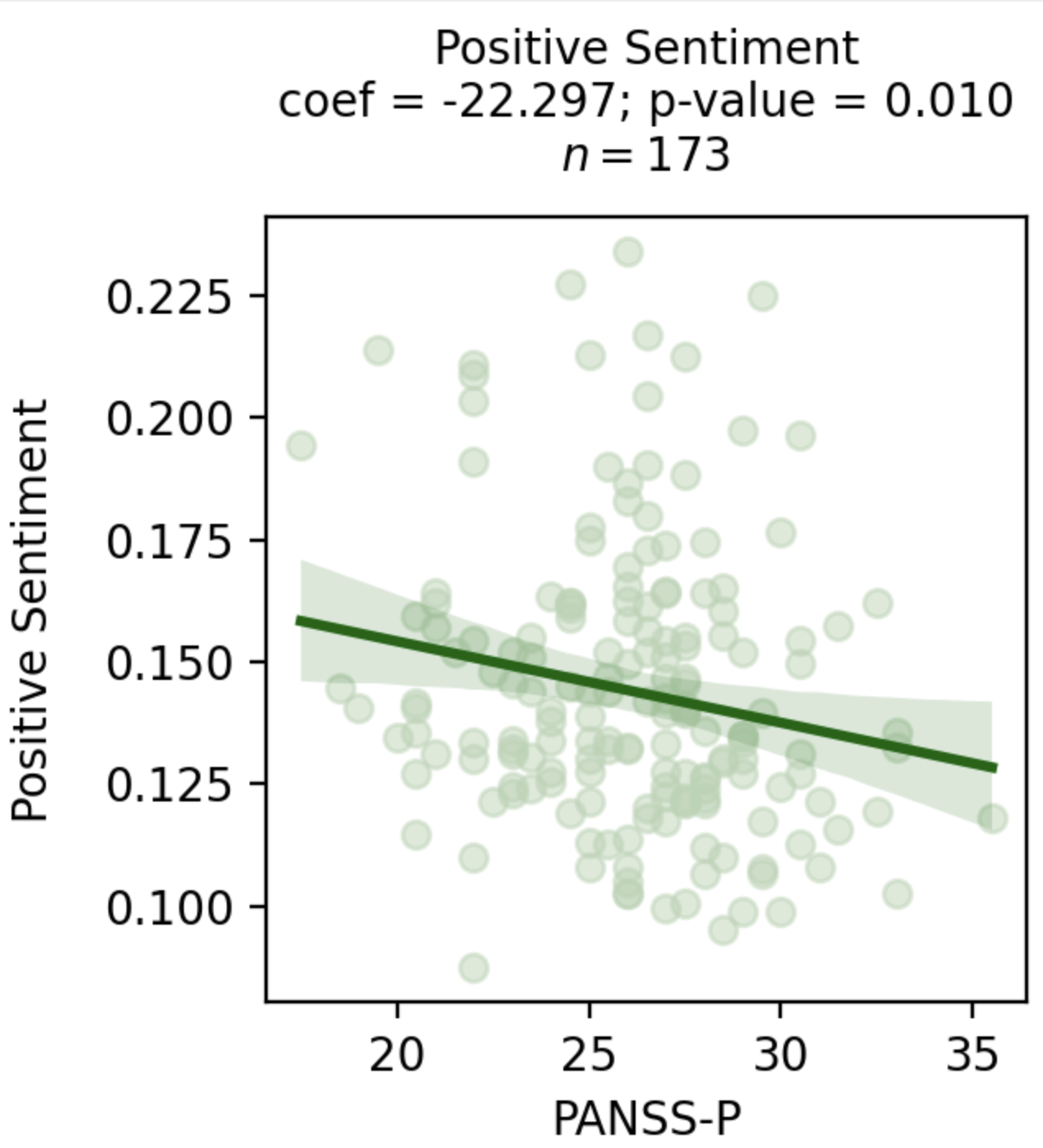 |  |  |  |
|  |  |  |  |

|  |  |  |  |
| --- | --- | --- | --- |
|  |  |  |  |

**Supplementary Figure 4. S**catter plots showing significant relationships between PANSS-N and speech characteristics as assessed by multiple linear regressions. Statistics specifically for the speech characteristics from the multiple linear regressions are shown above the graph, where age, sex, and race were included as covariates. This analysis used averaged values from the screening and baseline visits.

| 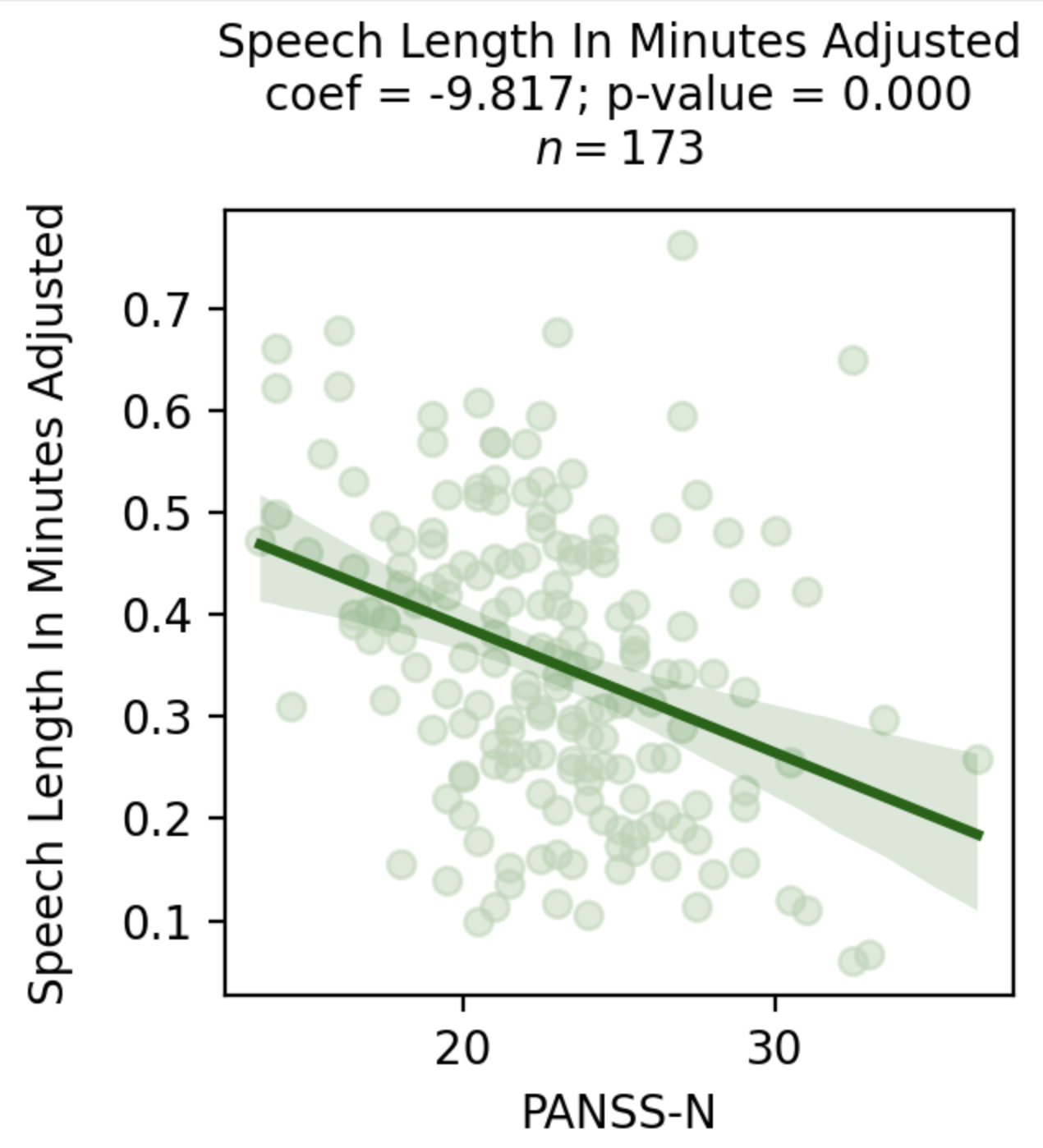 | 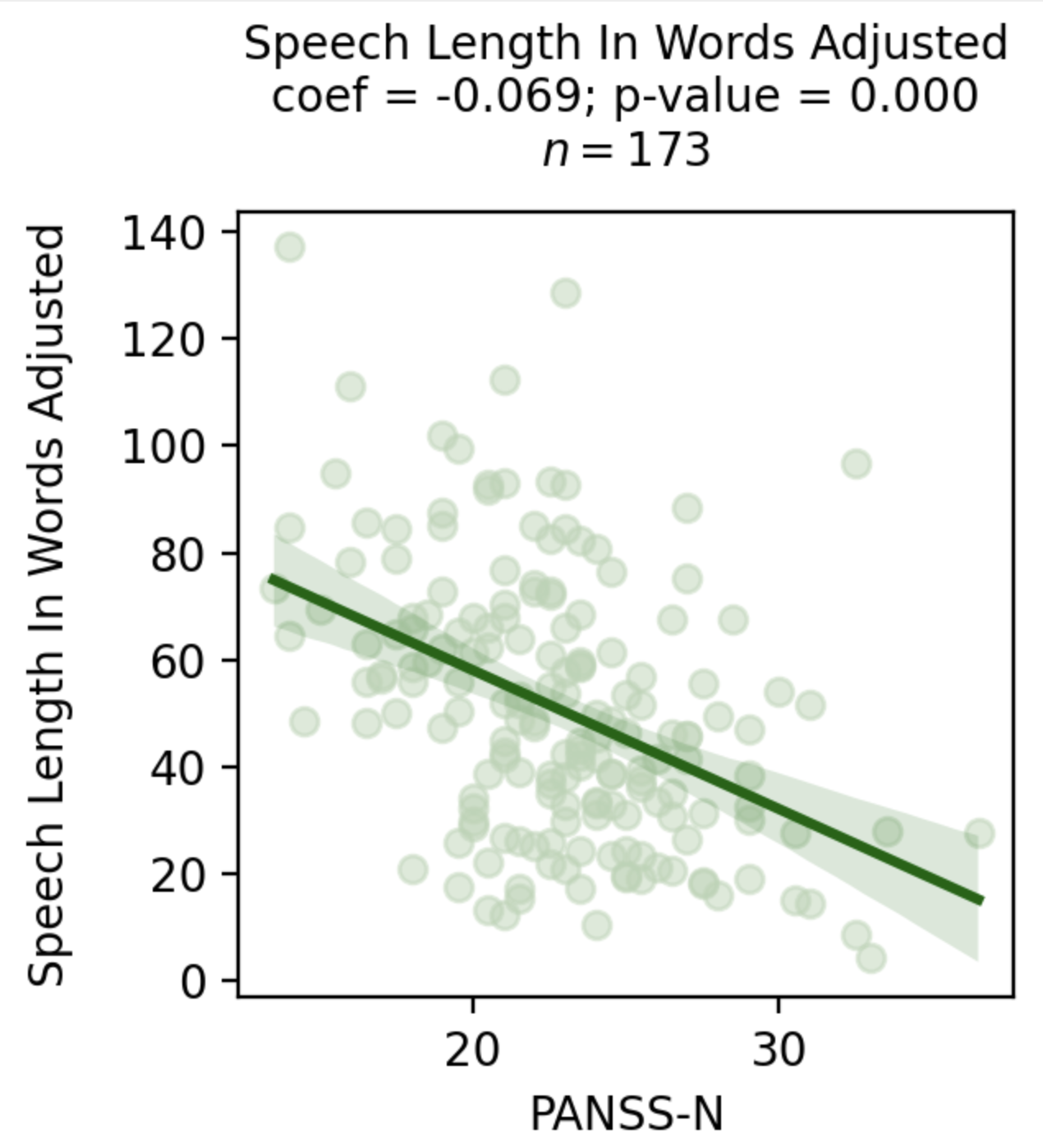 | 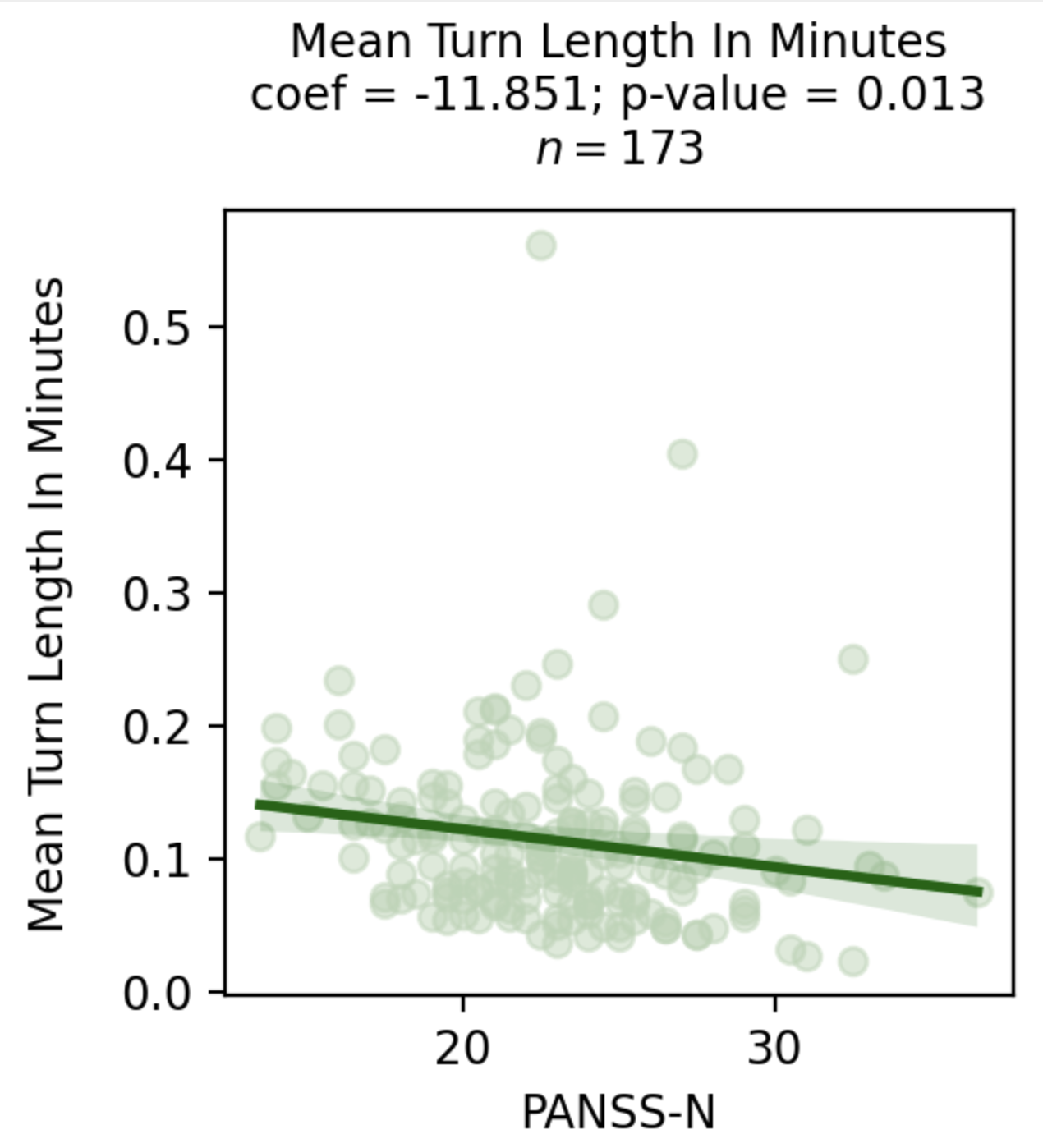 | 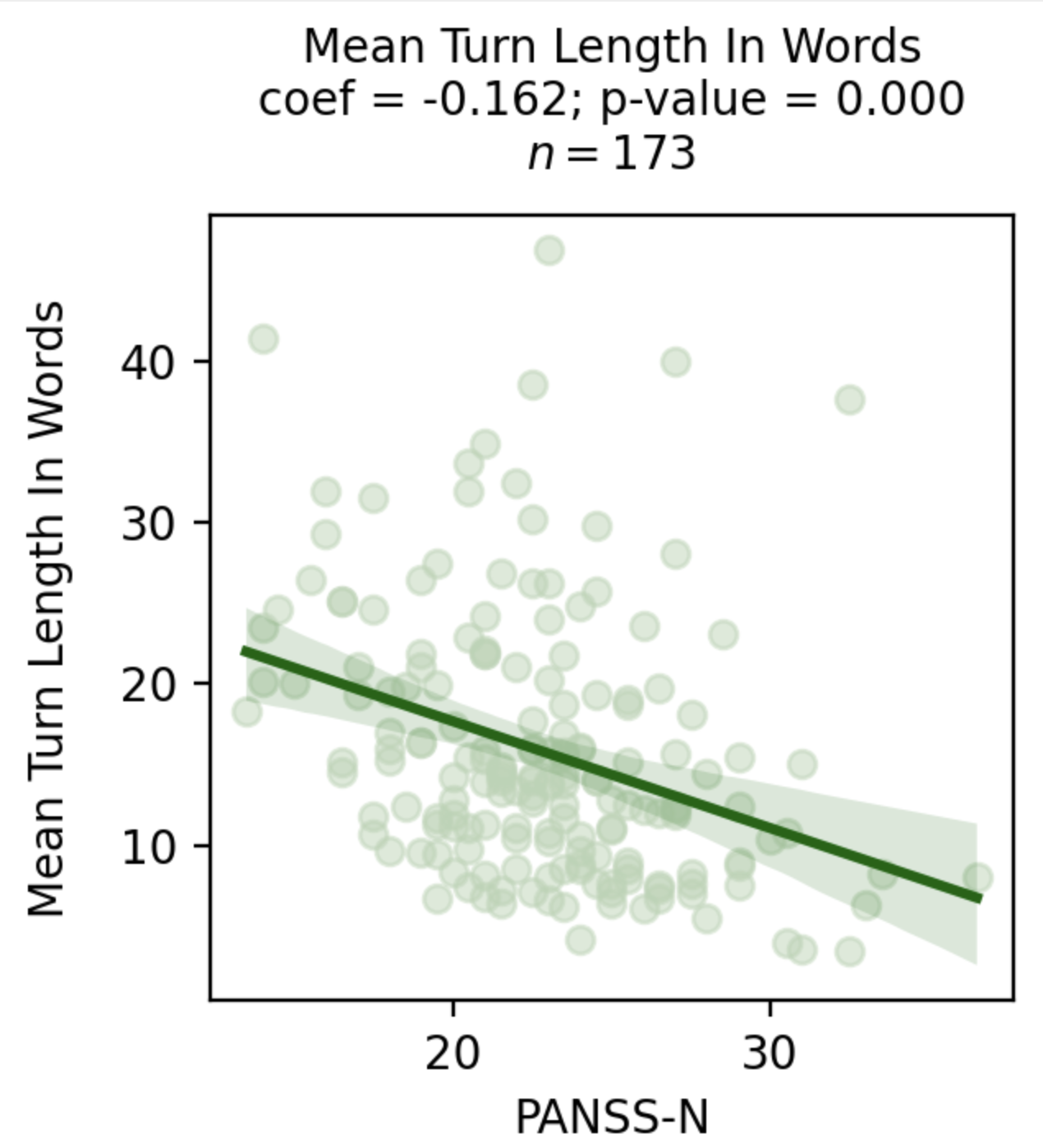 |
| --- | --- | --- | --- |
| 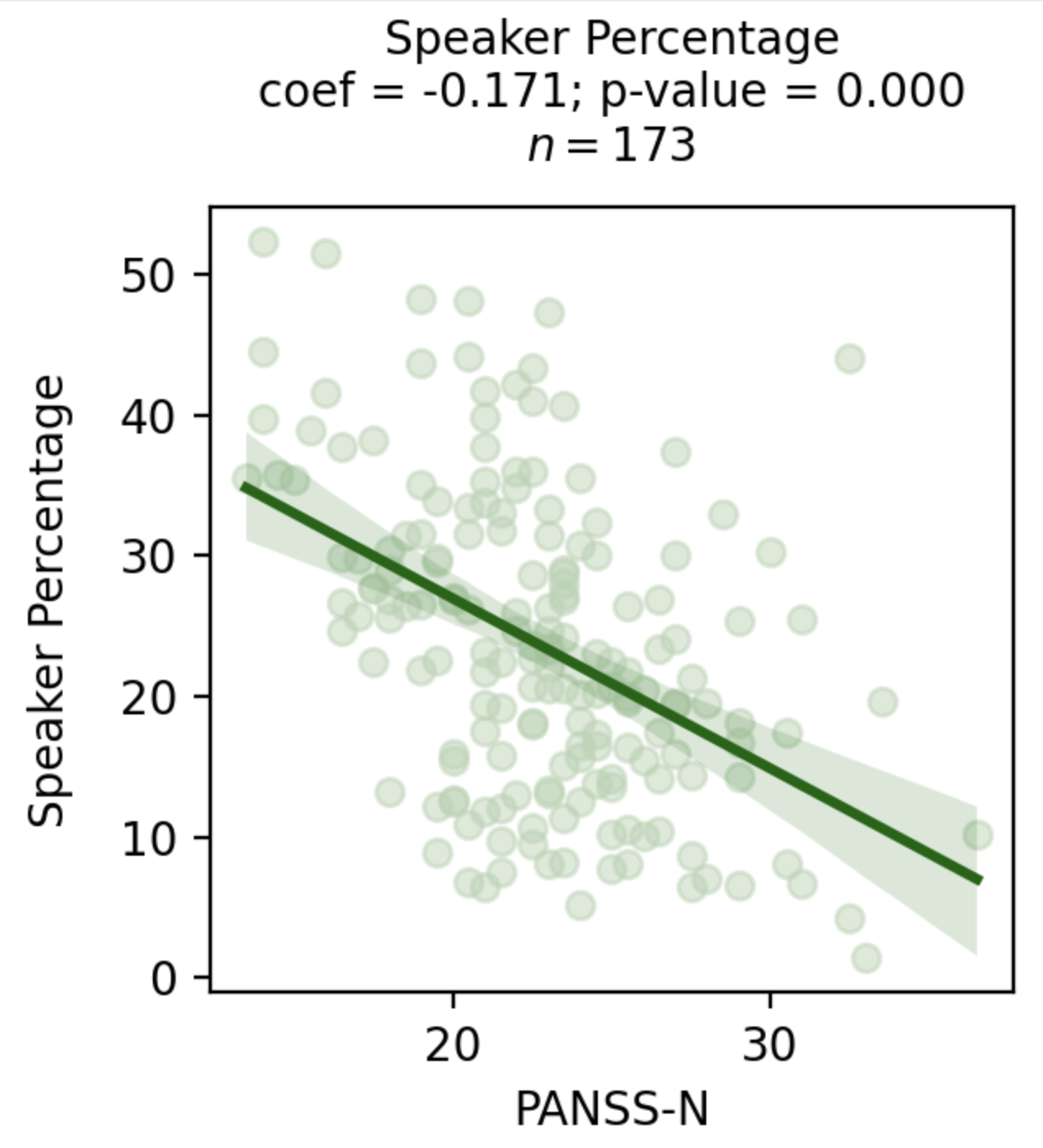 | 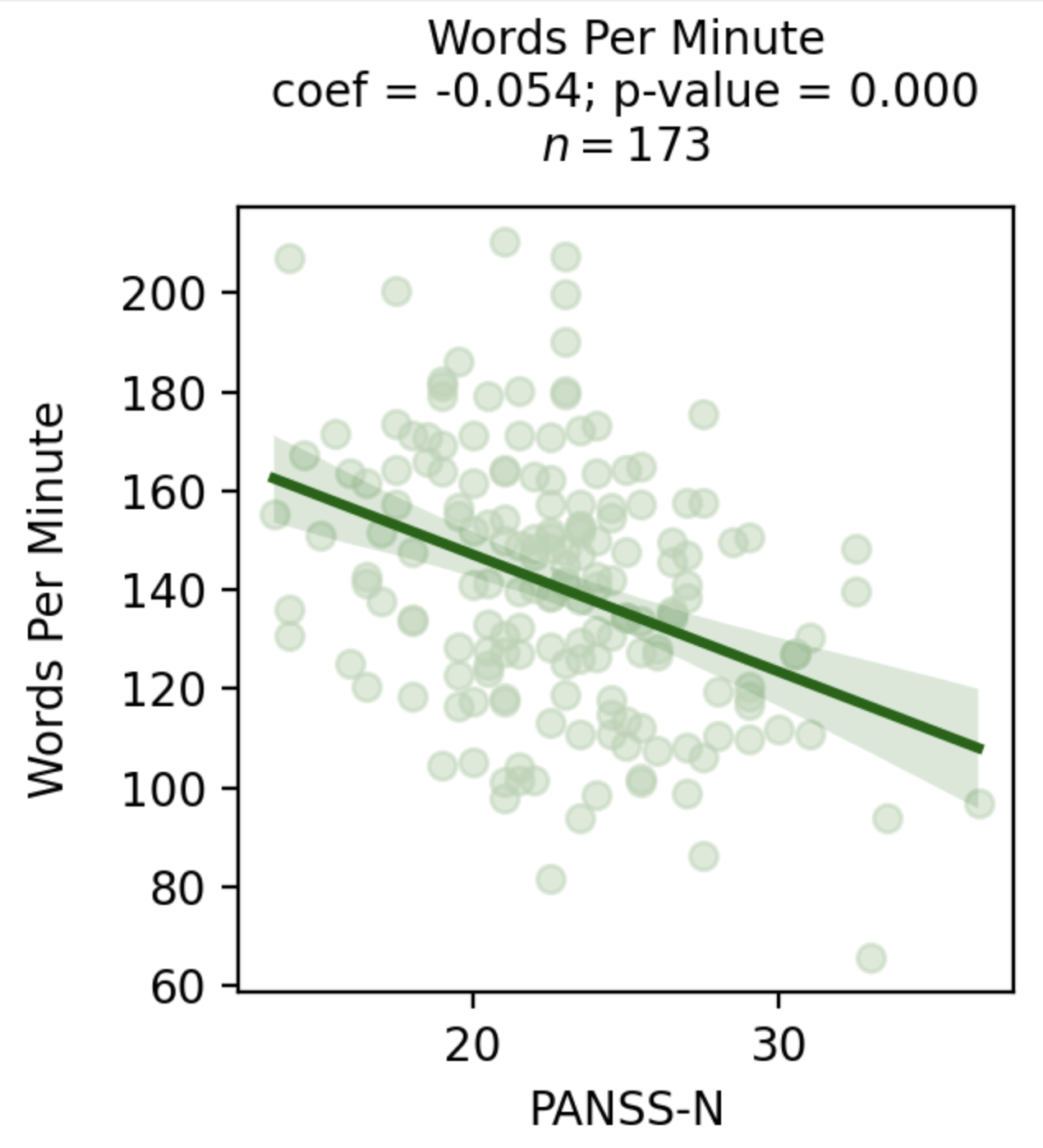 | 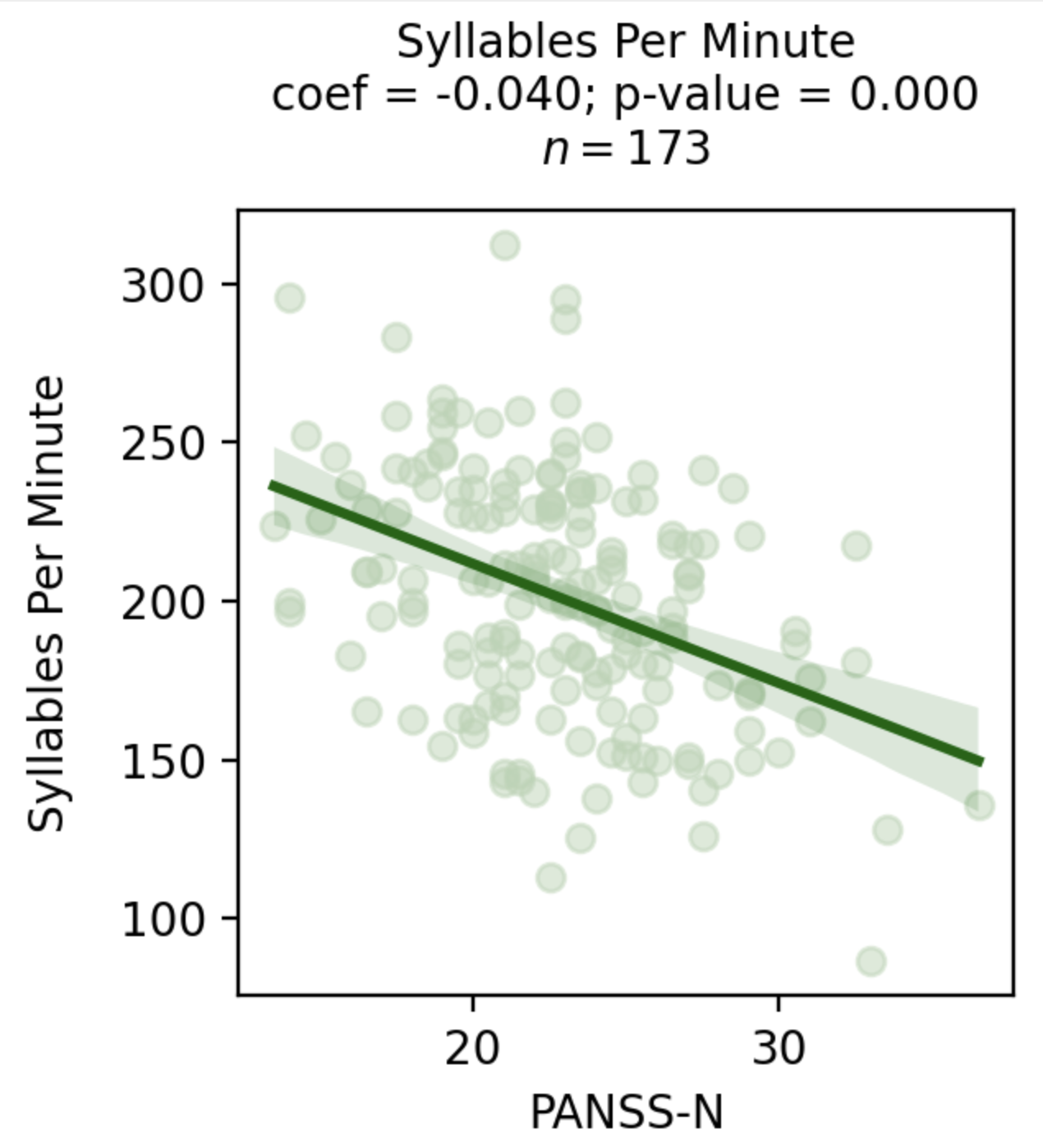 | 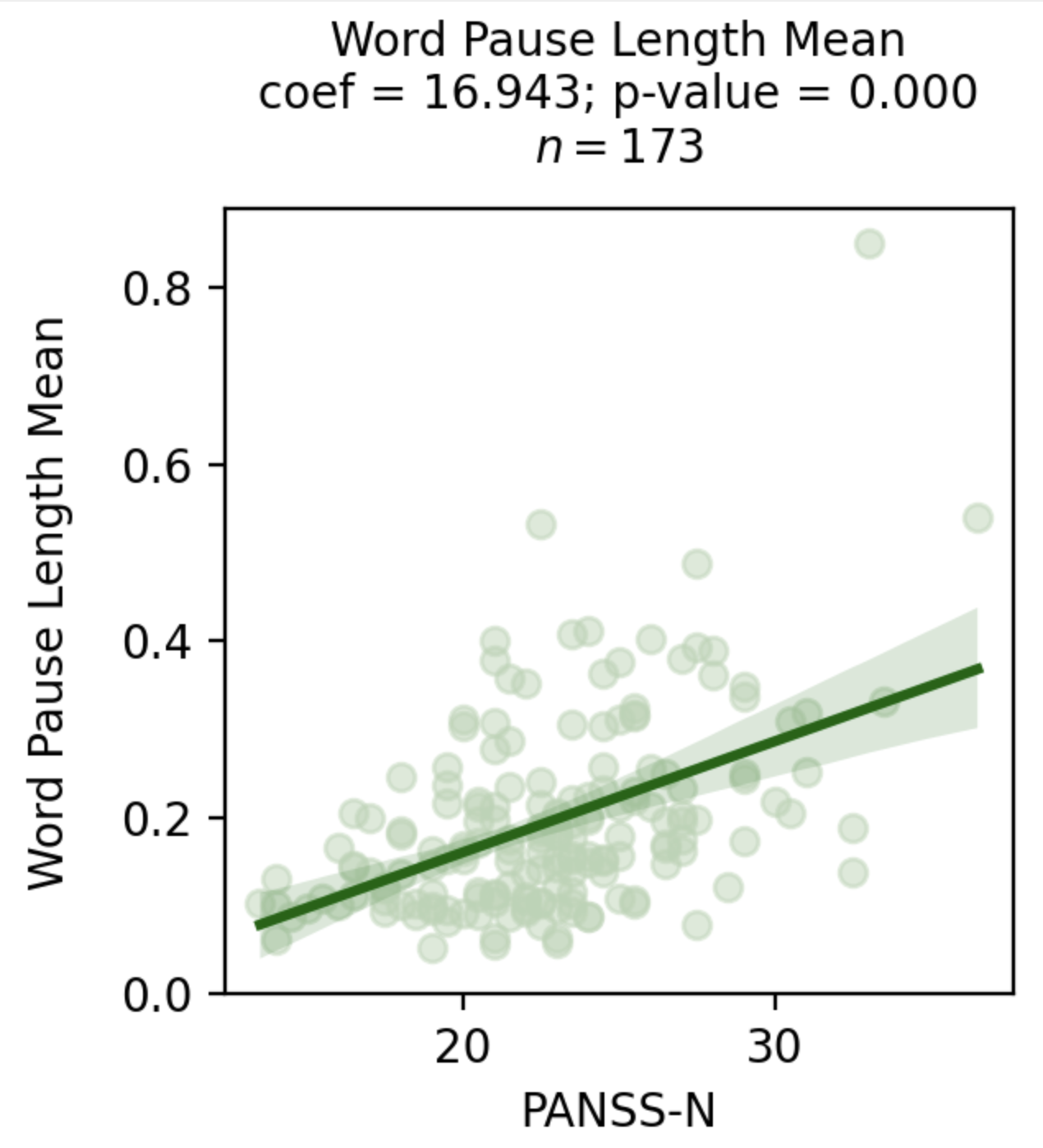 |
| 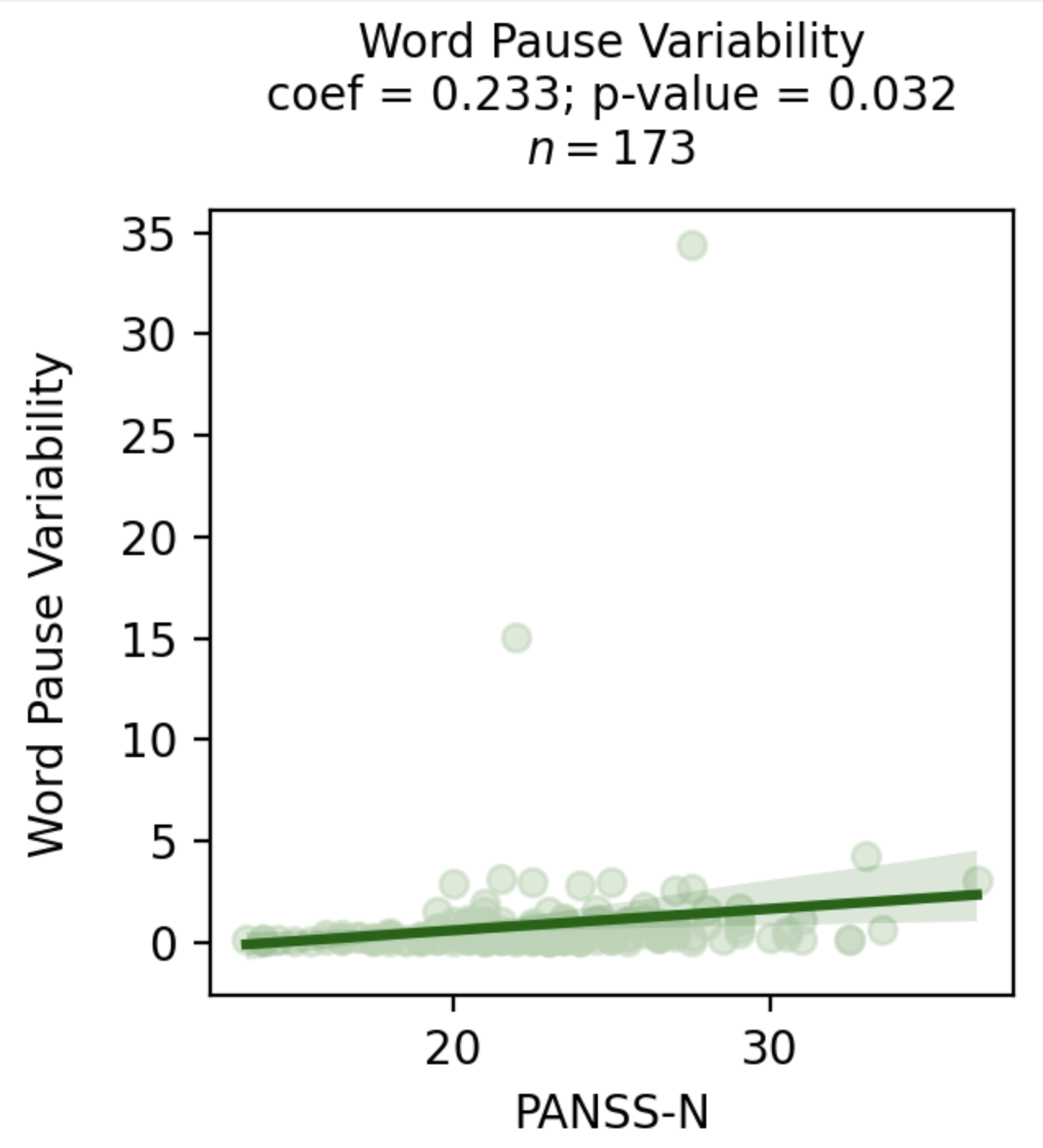 | 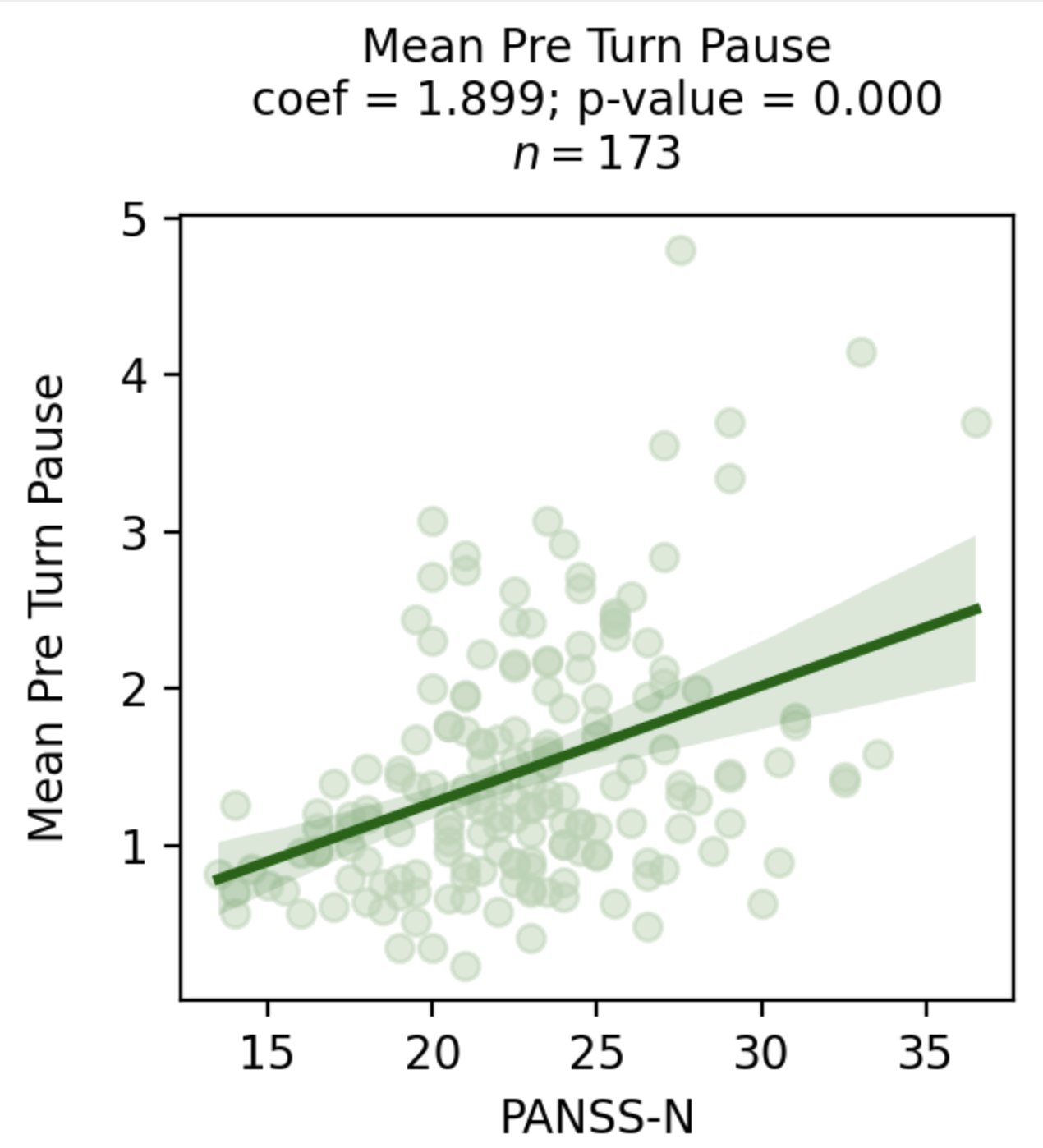 |  |  |
|  |  |  |  |

**Supplementary Figure 5.** Results from comparison of manual transcription and diarization with automated transcription and diarization methods used in this study. In the figures below, the top panel shows the audio signal recorded from the shared microphone. The subsequent two top rows indicate transcription of the clinician and patient using manual transcription (M, S1, S2) while the two bottom rows indicate transcription of the clinician and patient using automatic transcription (A, S1, S2). Discrepancies between the two are highlighted in red. Overall, we found a very high level of alignment between manual and automatic transcription.

**Excerpt from transcript 1:**

|  | 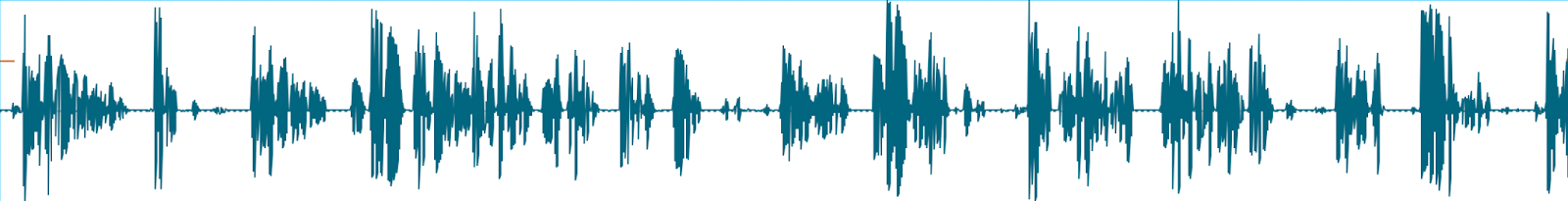 | | | | |  |
| --- | --- | --- | --- | --- | --- | --- |
| S1 | Have you been feeling worried or nervous in the past week? |  | Yeah. What’s been making you feel nervous or worried? |  | Mhm, mhm. | M |
| S2 |  | I have, yes. |  | Mm, I have, um, a lot of uh, responsibilities, I guess, and I’m trying to manage my time, uh, well, so I’m struggling a bit with, uh, making things, uh, go smoothly |  | M |
| S1 | Have you been feeling worried or nervous in the past week? |  | Yeah.What's been making you feel nervous or worried? |  | Mhm mhm. | A |
| S2 |  | I have, yes. |  | Mm, I have, uh, a lot of, uh, responsibilities, I guess, and I'm trying to manage my time, uh, well, so I'm struggling a bit with. Uh, making things. Uh, go smoothly. |  | A |

|  | 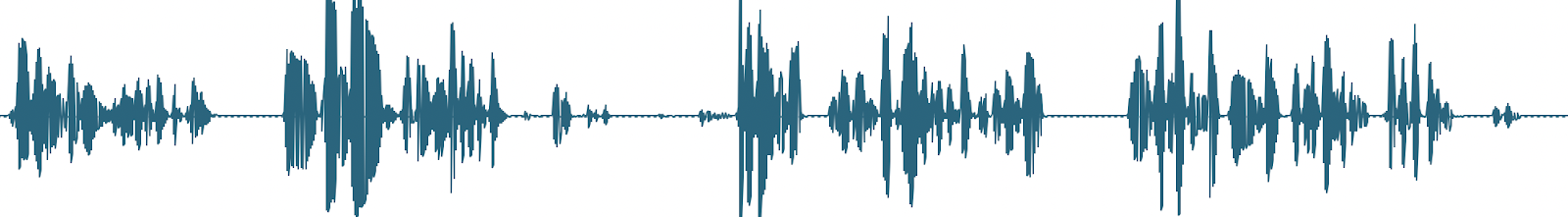 | | | |  |
| --- | --- | --- | --- | --- | --- |
| S1 | How nervous or worried would you say that you’ve been feeling? |  | Mhm. Ok. Have you found yourself shaking at times or that your heart has been racing? |  | M |
| S2 |  | Mm, I’ll say, maybe seven out of ten. |  | Mm, not shaking, but, uh, sure, maybe sometimes I can have my heart racing. | M |
| S1 | How nervous or worried would you say that you've been feeling? |  | Mm, OK. Have you found yourself shaking at times so that your heart has been racing? |  | A |
| S2 |  | Mm, I'll say maybe 7 out of 10. |  | Mm, not shaking, but, uh, sure, maybe sometimes I can have my heart racing. | A |

|  | 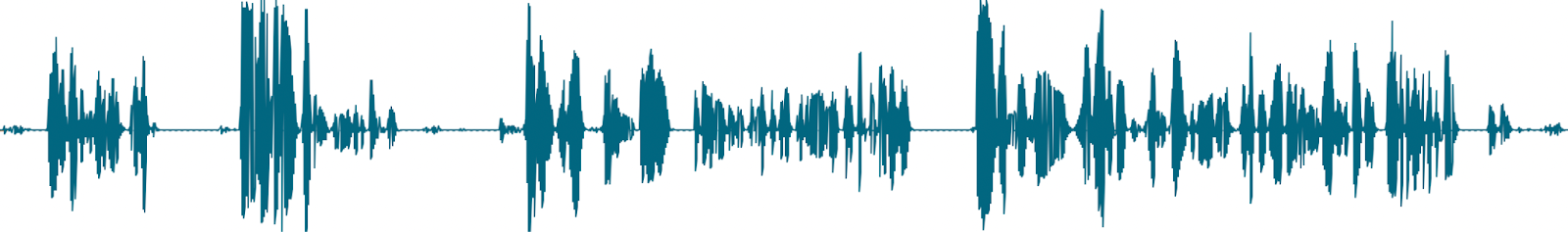 | | | |  |
| --- | --- | --- | --- | --- | --- |
| S1 | Mhm. Do you ever get into a state of panic? |  | Has your sleep, eating, or ability to participate in activities been affected by this? |  | M |
| S2 |  | Uh, no, I’ve never gotten into a state of panic, no. |  | Uh, yes, I’ll say, maybe I’m a bit less inclined to do any extracurricular activities or any sports, uh, that I used to do before. | M |
| S1 | Do you ever get into a state of panic? |  | Has your sleep, eating, or ability to participate in activities been affected by this? |  | A |
| S2 |  | Uh, no, I've never gotten into a state of panic, no. |  | Uh, yes, I'll say maybe I'm a bit less inclined to do any extracurricular activities or any sports, uh, that I used to do before. | A |

|  | 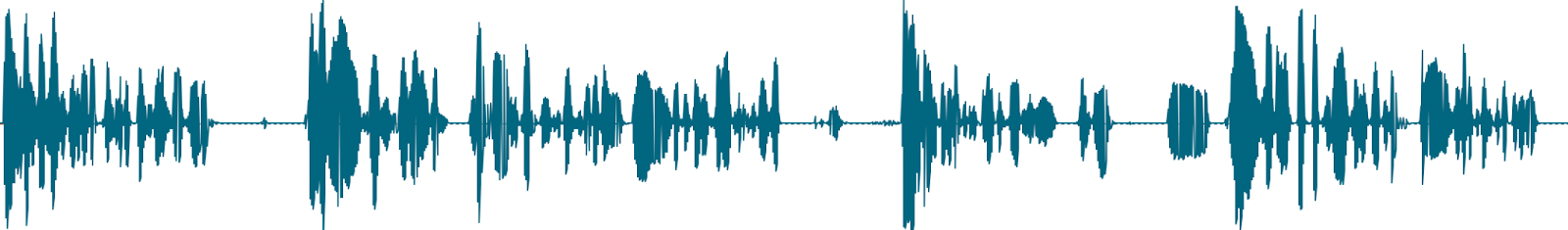 | | | |  |
| --- | --- | --- | --- | --- | --- |
| S1 | Mhm. Ok. Ok. And in general, would you say that things have been going well for you? |  | Okay. And has anything in particular been bothering you lately? |  | M |
| S2 |  | In general, yes, other than some, uh,  stressful situations that I’ve been, uh, I think yes, in general, things have been going well. |  | Mmm, no, nothing in particular, just work stuff, and some education stuff I guess. | M |
| S1 | OK. OK. And in general, would you say that things have been going well for you? |  | OK. And has anything in particular been bothering you lately? |  | A |
| S2 |  | In general, yes, other than some uh stressful situations that I've been, uh, I think yes, in general, things have been going well. |  | Mm, no, nothing in particular, just work stuff and some education stuff, I guess. | A |

|  | 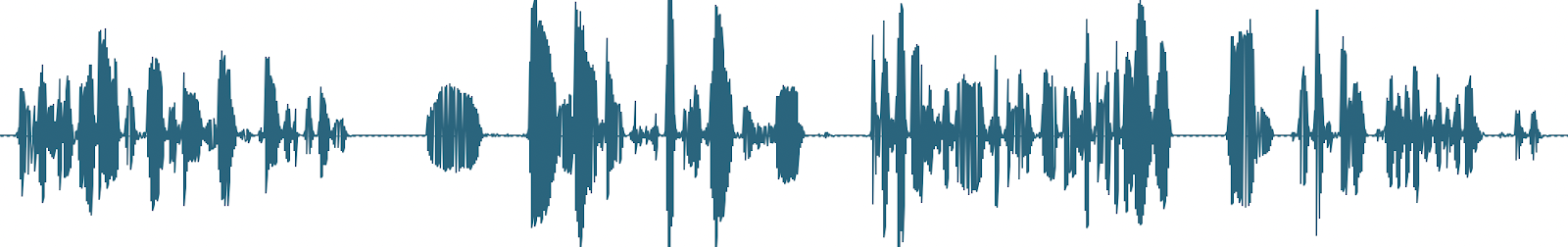 | |  |
| --- | --- | --- | --- |
| S1 | Okay. Can you tell me something about your thoughts on life and its purpose? |  | M |
| S2 |  | Mmm, uh, sure, I think the purpose of life is, uh, to be happy and also do something meaningful for yourself and your people around you, um, I try to follow this I guess as much as I can. | M |
| S1 | OK. Can you tell me something about your thoughts on life and its purpose? |  | A |
| S2 |  | Mm, uh, sure, I think the purpose of life is, uh, to be happy and also do something meaningful for yourself and your people around you, um, and I try to follow this, I guess as much as I can. | A |

**Excerpt from transcript 2:**

|  | 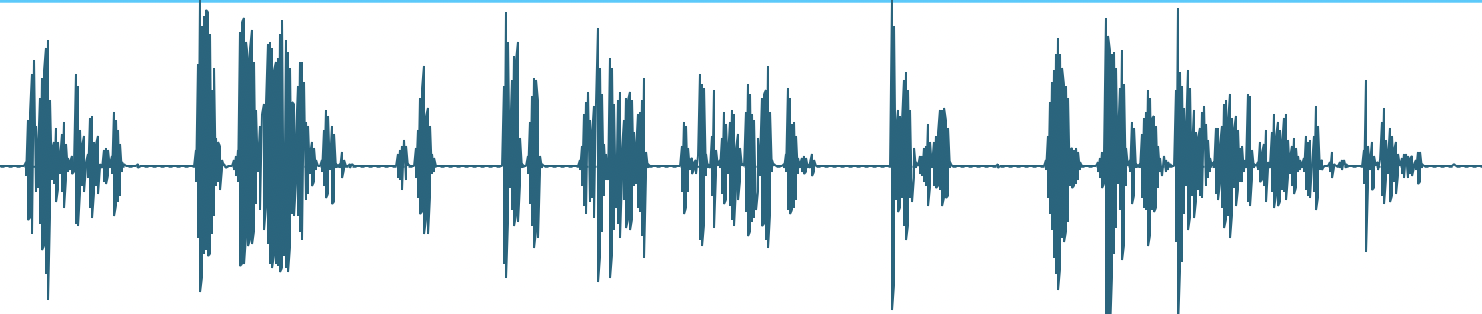 | | | | | |  |
| --- | --- | --- | --- | --- | --- | --- | --- |
| S1 | Do you feel like you can trust most people? |  | Hm, really. Why not? |  | Anyone in particular? |  | M |
| S2 |  | Um, no, I’d say I rarely trust people |  | Yeah, yeah I dunno, I just don’t find people in my experience trustworthy |  | Um, maybe people of, I dunno, strong positions, politicians, these kinds of people, are untrustworthy I mean. | M |
| S1 | Do you feel like you can trust most people? |  | Hm, really? Why not? Yeah, |  | Anyone in particular? |  | A |
| S2 |  | Um, no, I'd say I rarely trust people. |  | yeah, I, I don't know, I just don't find people in my experience trustworthy. |  | Um, maybe people of, I don't know, strong positions being politicians and these kinds of people, I don't trust at all, I mean. | A |

|  | 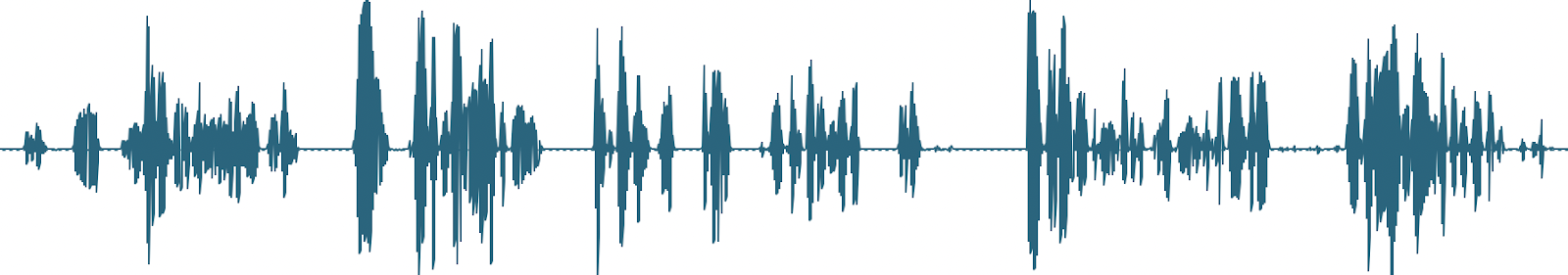 | | | |  |
| --- | --- | --- | --- | --- | --- |
| S1 | Okay. Do you, do you get along well with others, in general, do you think? |  | Okay. And what about, um, do you have, like a short fuse, a quick temper ever? |  | M |
| S2 |  | Um, some people yes, some people, well, it really depends. Uh, I get along with my close friends I guess, yeah. |  | Um, sure, yes I, I do get irritated quite easily. | M |
| S1 | OK. Do you, do you get along well with others in general, do you think? |  | OK. And what about um do you have like a, a short fuse, a quick temper ever? |  | A |
| S2 |  | Um, some people, yes, some people really depends. Uh, I get a lot with my close friends, I guess, yeah. |  | Um, sure, yes. I, I do get irritated pretty easily. | A |

|  | 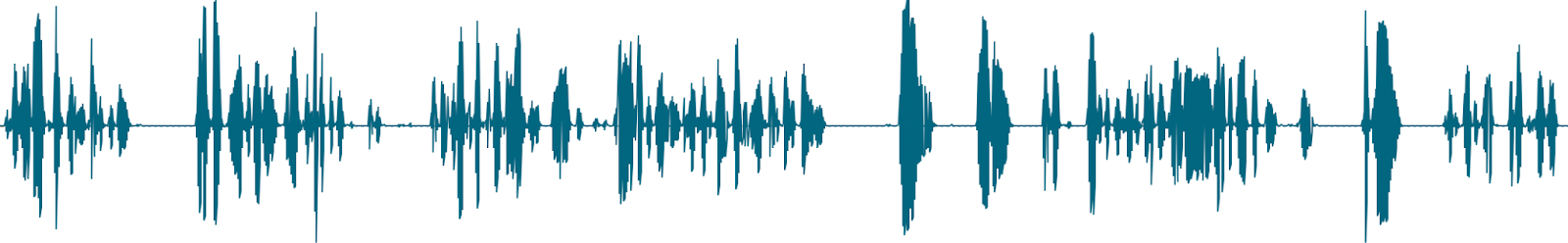 | | | |  |
| --- | --- | --- | --- | --- | --- |
| S1 | Ok, what about fights, do you fight with people? |  | Ok, so if you get provoked , so are you, so, tell me what happens, do you throw the first punch, do they throw the first punch? |  | M |
| S2 |  | Sometimes, I guess, um, if i get provoked, maybe |  | Um, I dunno, maybe the other people throw the first punch, uh, some people irritate me and maybe I push them back and they start the fights | M |
| S1 | OK. What about fights? Do you fight with people? |  | OK, so if you get provoked, so are you, so tell me what happens. Do you, do you throw the first punch? Do they throw the first punch? |  | A |
| S2 |  | Sometimes, I guess, um if I get provoked, I mean. |  | Um, I don't know, maybe the other people throw the first responses, uh, some people irritate me, and. Maybe I push them back and they start the fights. | A |

**Supplementary Table 1.** Regression coefficients and *p*-values of speech characteristics predicting both positive symptoms (PANSS-P) and negative symptoms (PANSS-N) as assessed by multiple linear regression models. Models were conducted on the averaged scores from the Screening and Baseline visits. Significant relationships are bolded and direction of association is shown for significant associations.

| **Category** | **Speech characteristic** | **PANSS-P** | | | **PANSS-N** | | |
| --- | --- | --- | --- | --- | --- | --- | --- |
|  |  | Direction of association | β | *p*-value | Direction of association | β | *p*-value |
| Amount of speech | Speaker percentage | + | 0.09 | **<0.001** | – | -0.17 | **<0.001** |
|  | Speech length, minutes | + | 6.4 | **<0.001** | – | -9.8 | **<0.001** |
|  | Speech length, words | + | 0.04 | **<0.001** | – | -0.07 | **<0.001** |
|  | Mean turn length, minutes |  | 5.8 | 0.12 | – | -11.9 | **0.01** |
|  | Mean turn length, words | + | 0.08 | **0.005** | – | -0.16 | **<0.001** |
| Rate of speech | Words per minute | + | 0.02 | **0.02** | – | -0.05 | **<0.001** |
|  | Syllables per minute | + | 0.01 | **0.04** | – | -0.04 | **<0.001** |
| Pause characteristics | Word pause length, mean | – | -4.7 | **0.04** | + | 16.9 | **<0.001** |
|  | Word pause variability |  | 0.11 | 0.20 | + | 0.23 | **0.03** |
|  | Mean pre-turn pause | – | -0.73 | **0.02** | + | 1.90 | **<0.001** |
| Emotional sentiment | Positive sentiment | – | -22.3 | **0.01** |  | 18.5 | 0.10 |
|  | Negative sentiment |  | -8.70 | 0.50 |  | 9.4 | 0.60 |
